# Prime editing-installed suppressor tRNAs for disease-agnostic genome editing

**DOI:** 10.1038/s41586-025-09732-2

**Published:** 2025-11-19

**Authors:** Sarah E. Pierce, Steven Erwood, Keyede Oye, Meirui An, Nicholas Krasnow, Emily Zhang, Aditya Raguram, Davis Seelig, Mark J. Osborn, David R. Liu

**Affiliations:** 1 Merkin Institute of Transformative Technologies in Healthcare, Broad Institute of Harvard and MIT, Cambridge, MA USA; 2 Department of Chemistry and Chemical Biology, Harvard University, Cambridge, MA USA; 3 Howard Hughes Medical Institute, Harvard University, Cambridge, MA USA; 4 Department of Veterinary Clinical Sciences, University of Minnesota, Minneapolis, MN USA; 5 Department of Pediatrics, University of Minnesota Medical School, Minneapolis, MN USA

**Keywords:** CRISPR-Cas9 genome editing, Gene therapy

## Abstract

Precise genome-editing technologies such as base editing^1,2^ and prime editing^3^ can correct most pathogenic gene variants, but their widespread clinical application is impeded by the need to develop new therapeutic agents for each mutation. For diseases that are caused by premature stop codons, suppressor tRNAs (sup-tRNAs) offer a more general strategy. Existing approaches to use sup-tRNAs therapeutically, however, require lifelong administration^4,5^ or show modest potency, necessitating potentially toxic overexpression. Here we present prime editing-mediated readthrough of premature termination codons (PERT), a strategy to rescue nonsense mutations in a disease-agnostic manner by using prime editing to permanently convert a dispensable endogenous tRNA into an optimized sup-tRNA. Iterative screening of thousands of variants of all 418 human tRNAs identified tRNAs with the strongest sup-tRNA potential. We optimized prime editing agents to install an engineered sup-tRNA at a single genomic locus without overexpression and observed efficient readthrough of premature termination codons and protein rescue in human cell models of Batten disease, Tay–Sachs disease and cystic fibrosis. In vivo delivery of a single prime editor that converts an endogenous mouse tRNA into a sup-tRNA extensively rescued disease pathology in a model of Hurler syndrome. PERT did not induce detected readthrough of natural stop codons or cause significant transcriptomic or proteomic changes. Our findings suggest the potential of disease-agnostic therapeutic genome-editing approaches that require only a single composition of matter to treat diverse genetic diseases.

## Abstract (web summary)

A new strategy that uses prime editing to convert an endogenous tRNA into a suppressor tRNA shows therapeutic potential for multiple genetic diseases that are caused by premature stop codons.

## Main

Therapeutic genome-editing efforts, including more than 70 clinical trials so far, have predominantly used programmable nucleases, base editors or prime editors to disrupt or correct disease-associated genes in an allele-specific manner. These approaches have proven to be effective in patients or in animal models for the treatment of disorders such as sickle-cell disease^6,7^, T cell leukaemia^8^, hypercholesterolaemia^9,10^, alpha-1-antitrypsin deficiency^10^, chronic granulomatous disease^11^, progeria^12^, spinal muscular atrophy^13^, prion disease^14^, alternating hemiplaegia of childhood^15^ and many other genetic diseases.

Although allele-specific therapeutic genome-editing strategies offer treatments for many serious diseases with few treatment options, the breadth of the global genetic disease crisis, in which more than 8,000 genetic diseases collectively affect hundreds of millions of patients, demands new approaches that more rapidly bring the benefits of therapeutic genome editing to large numbers of patients^16^. Allele-specific applications of nucleases, base editors and prime editors require the development of a distinct genome-editing treatment for each of the more than 200,000 known pathogenic mutations, although prime editing also enables corrections of clustered hotspots of mutations. Until substantial streamlining of regulatory, development and manufacturing costs occurs, the development of the thousands of distinct drugs needed to treat any large fraction of patients with genetic disease remains impractical, despite the fact that base editing and prime editing can collectively correct the vast majority of known pathogenic mutations^1–3^.

The versatility of prime editing, which uses a programmable nickase and a reverse transcriptase to replace targeted segments of DNA with new sequences of our choosing^3^, can be used in creative ways that may benefit far larger cross-sections of patients with genetic disease. Nonsense mutations account for 24% of pathogenic alleles in the ClinVar database^16^. The common molecular consequence of nonsense mutations—termination of translation before functional protein can be made—suggests more generalized approaches to their therapeutic rescue that might be applied in an allele-agnostic or even disease-agnostic manner.

Nonsense sup-tRNAs have an anticodon that complements a premature termination codon (PTC) and support the installation of an amino acid rather than termination of translation. Although sup-tRNAs can theoretically read through both PTCs and natural termination codons (NTCs), extensive evidence suggests that sup-tRNAs can be well-tolerated in eukaryotes. Both naturally occurring and exogenous sup-tRNAs can be expressed without apparent toxicity^4,5^. Multiple biological mechanisms explain the observation of surprisingly low levels of sup-tRNA-mediated readthrough at NTCs. First, the distribution of stop codons for PTCs is distinct from the distribution for NTCs, particularly increasing the safety profile of sup-tRNAs corresponding to the amber stop codon (TAG)^17^. Second, the frequent presence of redundant and diverse in-frame stop codons following NTCs reduces the likelihood of extending a protein past a suppressed stop codon by more than a few amino acids^18^. Third, recruitment of polypeptide chain release factors to the 3′ untranslated region (3′ UTR) near NTCs can outcompete sup-tRNAs^19^. Fourth, if the ribosome continues past the NTC, the RNA and protein are targeted for degradation through the non-stop decay pathway^20^, and proteins that are translated into the 3′ UTR are further recognized and targeted for degradation^21^. Finally, sup-tRNAs mediate nonsense suppression only in transcripts that are being expressed in a given cell, minimizing the risk of toxicity from ectopic overexpression.

Despite the promise of sup-tRNAs as a potential therapeutic strategy, current approaches such as lipid nanoparticle (LNP)-based or adeno-associated virus (AAV)-mediated delivery of sup-tRNAs^4,5^ face the challenge of supporting sup-tRNA production throughout the lifetime of the patient. AAV-based methods, in particular, may be limited to a single dose, owing to the generation of high levels of neutralizing antibodies following dosing, which may not be sufficient to treat genetic diseases that require restoration of protein expression for the lifetime of the patient. In addition, efforts to apply sup-tRNAs as therapeutic agents are stymied by a lack of complete understanding of the determinants of sup-tRNA potency. As a result, recent efforts to rescue nonsense mutations have relied on the overexpression or delivery of high levels of sup-tRNAs with suboptimal suppression efficiency^4,5^, even though tRNA overexpression can alter global translation^22–24^ and raise the possibility of toxicity. A one-time treatment that would enable permanent expression of endogenous levels of a sup-tRNA optimized for suppression efficiency without apparent toxicity has the potential to transform the treatment of diseases caused by PTCs. Because such a treatment may ameliorate a variety of diseases arising from the targeted type of stop codon, it also has the potential to benefit a large number of patients with multiple disorders caused by many pathogenic PTCs.

Here we evaluated tens of thousands of variants of all 418 high-confidence human tRNAs to engineer highly active sup-tRNAs with sufficient potency to mediate efficient nonsense mutation suppression even when expressed from a single genomic copy with endogenous regulatory elements. By iteratively optimizing: (1) the 40-bp leader sequence of the tRNAs; (2) the tRNA sequence via saturation mutagenesis; and (3) the terminator sequence of the tRNAs, we developed a highly active TAG-targeting sup-tRNA despite its expression remaining at sub-endogenous levels from a single genomic locus. We then used the versatility of prime editing to permanently convert a redundant endogenous human tRNA to these optimized sup-tRNAs. We call this strategy PERT (prime editing-mediated readthrough of premature termination codons).

In human cell models of nonsense mutations in genes known to cause Batten disease (*TPP1 *p.L211X and *TPP1 *p.L527X), Tay–Sachs disease (*HEXA *p.L273X and *HEXA *p.L274X) and Niemann–Pick disease type C1 (*NPC1 *p.Q421X and *NPC1 *p.Y423X), treatment with the same prime editor programmed to install an optimized sup-tRNA resulted in restoration of 20–70% of normal enzyme activity. We experimentally quantified the effectiveness of our prime editing-installed sup-tRNA for reading through all clinically relevant TAG PTCs in the ClinVar database, observing PTC readthrough for the vast majority of sequences tested. Finally, we applied PERT in vivo in a mouse with a co-delivered reporter construct containing a nonsense mutation in GFP, as well as in a mouse model of Hurler syndrome, a severe lysosomal storage disease caused by a premature stop codon (*IDUA *p.W392X). Treatment with a single composition of matter mediated around 25% production of full-length GFP in reporter mice, and therapeutic levels of approximately 6% IDUA enzyme activity restoration, resulting in nearly complete rescue of disease pathology in Hurler syndrome mice. These findings provide a foundation for the development of disease-agnostic therapeutic genome-editing strategies that have the potential to substantially increase the number of patients that can benefit from a single genome-editing drug.

## Low copy number limits sup-tRNA efficiency

The human genome encodes 47 isodecoder (anticodon) tRNA families comprising 418 high-confidence genes (Supplementary Table 1). This redundancy, coupled with transcription-induced mutagenesis, yields many tolerated tRNA mutations in humans, including anticodon mutations that create natural suppressor or mis-charged tRNAs^25,26^. Fourteen isodecoder families have no corresponding tRNA gene and are decoded by pseudocognate tRNAs^27^, and entire tRNA families have been deleted without consequence^28^. Therefore, we hypothesized that converting a single endogenous tRNA to a sup-tRNA with prime editing could be well-tolerated and enable readthrough of nonsense mutations regardless of the gene in which they originate. This approach avoids tRNA overexpression that can perturb global translation^22–24^ and preserves native tRNA regulation.

**Extended Data Fig. 1:**
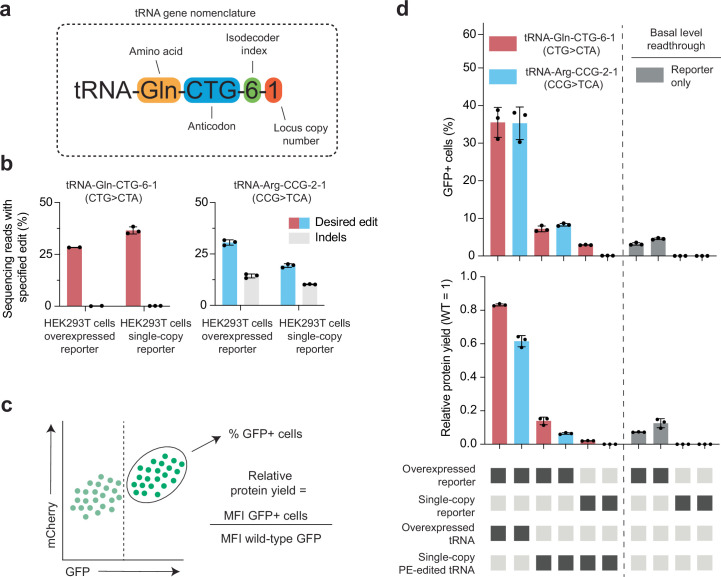
Prime editing-mediated conversion of two endogenous tRNA genes into sup-tRNAs. (**a**) Schematic of standard tRNA nomenclature. For example, in indicating “tRNA-Gln-CTG-6-1”: Gln indicates the amino acid that the tRNA normally carries (here, glutamine); CTG is the anticodon sequence that recognizes the corresponding codon in mRNA; 6 is the isodecoder index, which distinguishes different tRNA genes that share the same amino acid and anticodon but differ slightly in sequence elsewhere; and 1 is the locus copy number, identifying which genomic copy of this isodecoder is being referenced. (**b**) Sequencing reads with the specified edit for two endogenous tRNAs that were converted to sup-tRNAs with prime editing. (**c**) Schematic of readthrough quantification from flow cytometry data. MFI, mean fluorescence intensity. Values and error bars reflect mean ± s.d. of n = 3 independent biological replicates. (**d**) % GFP+ cells (*top*) and relative protein yield (*bottom*) for two sup-tRNAs delivered via transfection or as a single-copy PE-edited tRNA, paired with either an overexpressed reporter or a single copy reporter.

To screen endogenous human tRNAs for their ability to become sup-tRNAs, we designed mCherry-STOP-GFP reporters in which GFP expression occurs only after PTC readthrough (Supplementary Fig. 1a–e and Methods). Next, we designed prime editing reagents to convert the anticodon loops of two endogenous tRNAs (tRNA-Gln-CTG-6-1 and tRNA-Arg-CCG-2-1) into sup-tRNAs. For clarity, we follow standard tRNA nomenclature throughout this work. For example, in tRNA-Gln-CTG-6-1: Gln indicates the associated amino acid; CTG indicates the anticodon; 6 is the isodecoder index that distinguishes tRNA genes in the same family with minor sequence differences; and 1 is the locus copy number (Extended Data Fig. 1a).

We installed these sup-tRNAs at the endogenous loci of tRNA-Gln-CTG-6-1 and tRNA-Arg-CCG-2-1 using prime editing in HEK293T cells. We observed on average 29% conversion of each endogenous tRNA into a sup-tRNA (range: 19%–37%) (Extended Data Fig. 1b). We then overexpressed the reporter construct via plasmid transfection, or expressed it from a single gene copy using lentiviral transduction. We characterized PTC readthrough using two metrics: the percentage of GFP-positive cells (% GFP) and the GFP mean fluorescence intensity relative to a wild-type GFP control (relative protein yield) (Extended Data Fig. 1c). Although both sup-tRNAs led to GFP rescue with an overexpressed reporter, neither led to significant PTC readthrough with a single-copy reporter (Extended Data Fig. 1d). Even with an overexpressed reporter, the per cent rescue of GFP relative to a wild-type control was modest, with on average 7.8% GFP-positive cells and a relative GFP protein yield of 10%, and overexpression of the reporter alone without any sup-tRNA yielded 3.9% GFP-positive cells, presumably from pseudocognate tRNA-mediated PTC readthrough.

Previous sup-tRNA studies have heavily relied on high tRNA levels in transfection, LNP or viral settings, often using elevated copy numbers of the sup-tRNA and/or the PTC-containing mRNA^4,5^. These findings revealed the need to identify sup-tRNAs that are sufficiently potent to mediate endogenous genomic PTC readthrough at low sup-tRNA levels that are compatible with genomic installation and minimize the risk of perturbing global translation.

## Screening prime editing-installed sup-tRNAs

**Fig. 1:**
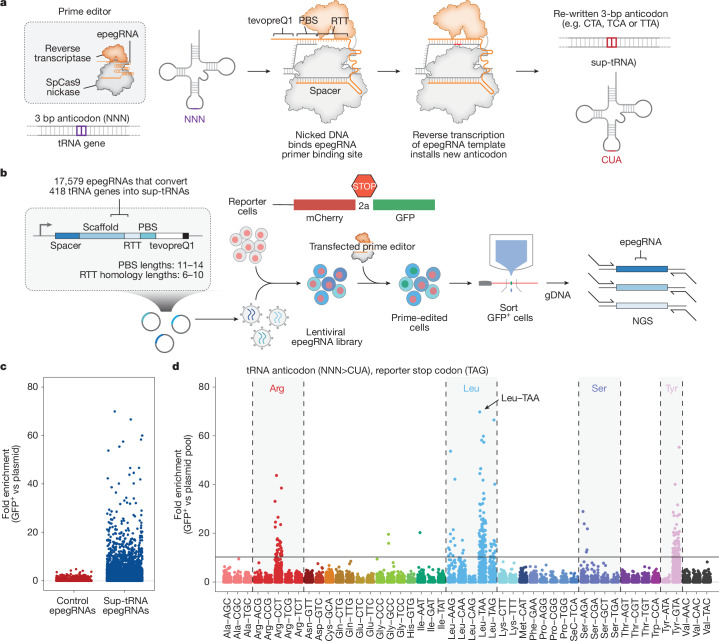
Prime editing-mediated conversion of endogenous tRNAs to sup-tRNAs in mammalian cells. **a**, Schematic of prime editing strategy to generate sup-tRNAs. **b**, Lentiviral epegRNA design and screening strategy. gDNA, genomic DNA; NGS, next-generation sequencing. **c**, Fold enrichment of epegRNAs, designed as controls (red) or to generate sup-tRNA (blue), in GFP-sorted cells, compared to the plasmid pool in the TAG screen. **d**, Fold enrichment of epegRNAs, colour-coded by the amino acids of the isoacceptor tRNA family that they target.

To comprehensively identify sup-tRNAs that promote stop codon readthrough with low copy number expression, we designed three prime editing screens to convert the anticodon of each endogenous human tRNA (*n* = 418 genes) to CUA, UCA, or UUA anticodons, thereby generating sup-tRNAs that recognize TAG, TGA or TAA stop codons, respectively (Fig. 1a and Supplementary Fig. 2). For each tRNA gene, we identified the two nearest 20-bp spacers flanking the anticodon with an NGG protospacer-adjacent motif and varied primer-binding site (PBS) lengths (11–14 bp) and reverse transcription template (RTT) homology lengths (6–10 bp), where feasible. To enhance editing efficiency, we appended the structured RNA motif tevopreQ1 to each prime editing guide RNA (pegRNA), creating engineered pegRNAs (epegRNAs)^29^. In total, we designed 17,579 epegRNAs for each of the three sup-tRNA anticodons plus 420 negative-control epegRNAs (Fig. 1b, Supplementary Fig. 3a and Supplementary Table 2).

**Extended Data Fig. 2:**
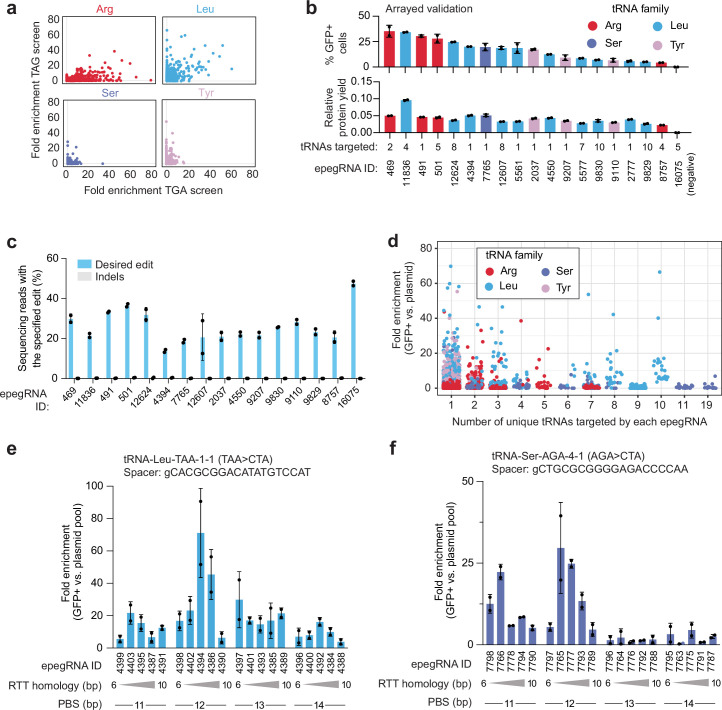
Endogenous tRNA genes can be converted to TAG sup-tRNAs with prime editing. (**a**) Comparison of fold enrichment between TAG and TGA stop codon reporters. Values and error bars reflect mean ± s.d. of n = 2 independent biological replicates. (**b**) Validation of top-performing epegRNAs in an arrayed transfection format. (**c**) Sequencing reads with the specified edit for 16 epegRNAs validated individually from the TAG PE2 screen. (**d**) Fold enrichment in GFP-sorted cells compared to plasmid pool for arginine, serine, leucine, and tyrosine tRNA isoacceptors. (**e**,**f**) Fold enrichment in GFP-sorted cells compared to plasmid pool for epegRNAs targeting the endogenous tRNA-Leu-TAA-1-1 (e) and tRNA-Ser-AGA-4-1 (f) genes with the indicated spacers, RTT homology lengths, and PBS lengths. Values and error bars reflect mean±s.d. of n = 2 independent biological replicates.

**Extended Data Fig. 3:**
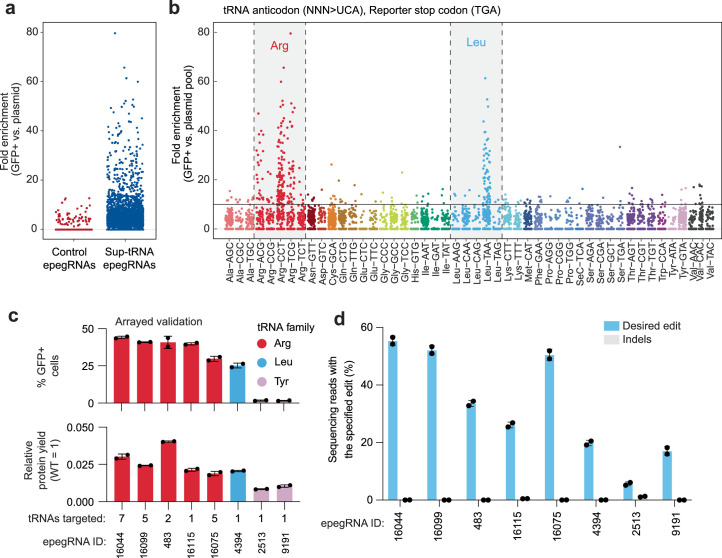
Endogenous tRNA genes can be converted to TGA sup-tRNAs with prime editing. (**a**) Fold enrichment of epegRNAs generating sup-tRNAs compared to controls in GFP sorted cells compared to the plasmid pool in the TGA screen. (**b**) Fold enrichment of epegRNAs, color-coded by the amino acids of the isoacceptor tRNA family that they target in the TGA screen. (**c**) Validation of top-performing epegRNAs in an arrayed transfection format in the TGA screen. (**d**) Sequencing reads with the specified edit for 8 epegRNAs validated individually from the TGA PE2 screen. Values and error bars reflect mean±s.d. of n = 2 independent biological replicates.

We cloned the epegRNA libraries into a lentiviral backbone and transduced them into a corresponding HEK293T cell line containing the mCherry-STOP-GFP reporter. Fluorescence-activated cell sorting (FACS) isolation of GFP-positive cells enriched cells expressing epegRNAs that generate functional sup-tRNAs (Fig. 1b,c and Supplementary Figs. 3 and 4). We identified arginine, leucine, tyrosine and serine tRNA backbones capable of reading through TAG stop codons (Fig. 1d and Extended Data Fig. 2a–f) and leucine and arginine tRNA backbones that read through TGA stop codons (Extended Data Fig. 3a–d and Supplementary Fig. 5). A similar screen for TAA stop codons did not yield effective sup-tRNAs (Supplementary Fig. 6), consistent with previous observations that TAA is the most stringent stop codon and is more resistant to sup-tRNA-mediated readthrough^30^.

Whereas epegRNAs that simultaneously targeted multiple tRNA sequences generally performed best, half of the most enriched positive hits in the TAG screen were epegRNAs targeting a single tRNA (Extended Data Fig. 2b–d). Although the epegRNA screen successfully identified endogenous tRNAs that could be converted into effective sup-tRNAs, even the best-performing sup-tRNA from this screen achieved less than 5% protein yield relative to a wild-type GFP control (Extended Data Fig. 2b). To address this limitation, we next engineered all major features of each sup-tRNA to enhance readthrough efficiencies.

## Exogenous promoter screen

**Extended Data Fig. 4:**
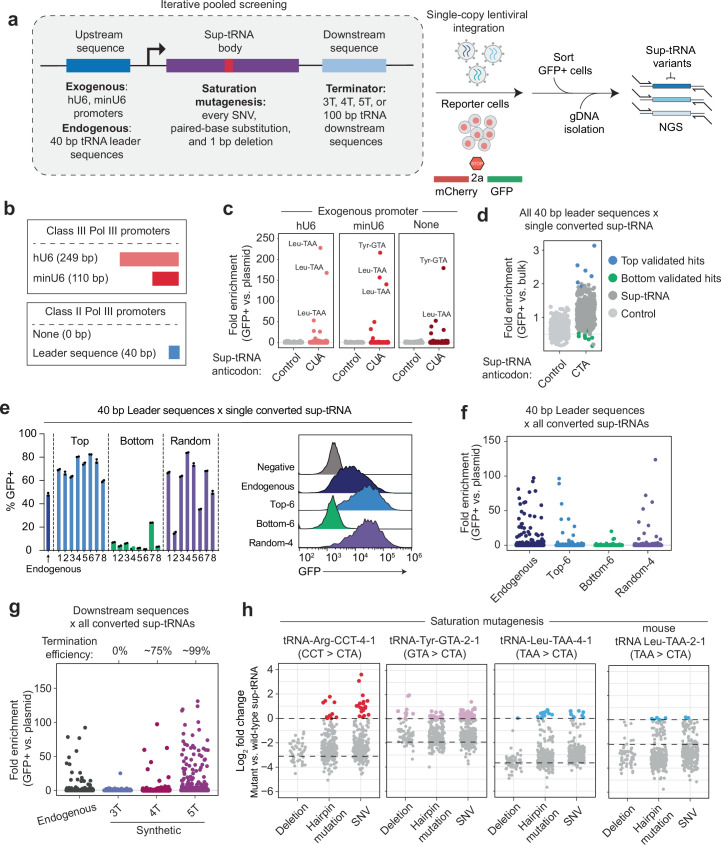
Engineering highly active sup-tRNA sequences. (**a**) Iterative pooled screening pipeline for lentiviral sup-tRNA optimization. (**b**) Comparison of Pol III promoters and their respective sizes for sup-tRNA expression. (**c**) Fold enrichment of sup-tRNA variants in GFP sorted cells compared to the plasmid pool representation when driven by a hU6, minU6, or endogenous promoter with no specified leader sequence (no exogenous promoter). Variants are separated by controls (with a non-sup-tRNA anticodon) or sup-tRNAs with a CUA anticodon. (**d**) Fold enrichment in GFP sorted cells compared to bulk transduced cells for a single sup-tRNA (tRNA-Leu-TAA-3-1, UAA > CUA) downstream of 418 distinct tRNA leader sequences. Top and bottom hits that were validated independently are indicated in blue and green. (**e**) Arrayed lentiviral validation of a single sup-tRNA (tRNA-Leu-TAA-4-1, UAA > CUA) downstream of the top and bottom hits from the screen in (d) compared to the endogenous leader sequence for the indicated tRNA as well as randomly designed synthetic 40-bp sequences. (**f-g**) Fold enrichment of sup-tRNA variants in GFP sorted cells compared to plasmid pool separated based on upstream (f) or downstream (g) sequence. (**h**) Log_2_ fold change of sup-tRNA saturation mutagenesis variants compared to an anticodon-only sup-tRNA for tRNA-Arg-CCT-4-1 (CCU > CUA), tRNA-Tyr-GTA-2-1 (GUA > CUA), tRNA-Leu-TAA-4-1 (UAA > CUA), or mouse tRNA-Leu-TAA-2-1 (UAA > CUA). Values and error bars reflect mean ± s.d. of n = 2 independent biological replicates.

A eukaryotic tRNA gene contains three distinct elements: the leader sequence, the tRNA gene body and the termination sequence (Extended Data Fig. 4a). Whereas tRNA expression is typically driven by an internal Pol III promoter, the leader sequence is thought to regulate gene expression^31^. Thus far, most sup-tRNA studies have relied on exogenous promoters for expression^5^, although the role of internal tRNA promoters is being investigated^32^.

To test tRNA promoter variants, we designed single-copy lentiviral constructs with: (1) an exogenous human U6 promoter (hU6); (2) a minimal human U6 promoter lacking the nucleosome positioning element (minU6)^33^; or (3) no exogenous promoter (Extended Data Fig. 4b). We performed three screens using a pool of all 418 mature tRNA sequences, modifying their anticodons to CUA, UCA or UUA to target TAG, TGA or TAA stop codons, respectively. The pooled sup-tRNA libraries were cloned into lentiviral constructs with each promoter variant and transduced into the mCherry-STOP-GFP reporter lines (Supplementary Fig. 2 and Supplementary Table 3).

Unlike the prime editing screen, lentiviral expression of sup-tRNAs with exogenous promoters yielded fewer TAG sup-tRNAs and activity depended on promoter choice (Extended Data Fig. 4c and Supplementary Fig. 7a). Leu-TAA sup-tRNAs were strongest with hU6 (tRNA-Leu-TAA-4-1, tRNA-Leu-TAA-3-1, tRNA-Leu-TAA-1-1 and tRNA-Leu-TAA-2-1, in descending order of activity), whereas Tyr-GTA (tRNA-Tyr-GTA-7-1) performed best with minU6 or no promoter, suggesting that this tRNA may have stronger internal promoter elements (Extended Data Fig. 4c). No effective TGA sup-tRNAs were identified when expressing the sup-tRNAs from a lentiviral construct (Supplementary Fig. 7b). Since exogenous promoters seem to hinder the proper expression of many sup-tRNAs identified in the prime editing screen, we next sought to investigate how endogenous leader sequences and terminator sequences affect sup-tRNA function.

## Sup-tRNA leader and terminator sequences

To further evaluate the effect of leader sequences, we analysed the 40 bp upstream of all 418 high-confidence human tRNAs. These sequences are less conserved than the tRNA gene bodies and may influence their expression^31,32^. We cloned each leader sequence upstream of a sup-tRNA candidate (tRNA-Leu-TAA-3-1) (Supplementary Fig. 2 and Supplementary Table 4), transduced this pool of constructs into the mCherry-STOP-GFP reporter cell line, and isolated GFP-positive cells via FACS. Eighty-six per cent of the cells expressing a sup-tRNA in this screen were GFP-positive, indicating that most leader sequences support sup-tRNA expression (Extended Data Fig. 4d). We individually tested leader sequences with the highest and lowest enrichment among GFP-positive cells, as well as eight randomly generated control sequences. The top hits performed well (averaging 72% GFP-positive cells) and the bottom hits performed poorly (averaging 6.1% GFP-positive cells), but the random leader controls also performed reasonably well (averaging 57% GFP-positive cells) (Extended Data Fig. 4e). Thus, whereas many diverse leader sequences can support robust sup-tRNA activity, others can nearly abolish activity. No apparent leader consensus sequences explained the observed variation in sup-tRNA activity.

Next, we investigated whether leader sequences could modulate PTC suppression levels across different sup-tRNAs. We selected six types of leader sequences: (1) each tRNA’s own endogenous leader; (2) a top-performing leader (Top-6); (3) a bottom-performing leader (Bottom-6); (4) a random leader (Random-4) (Extended Data Fig. 4e); and two leaders upstream of highly expressed tRNAs: (5) tRNA-Cys-GCA-3-1; and (6) tRNA-Ser-GCT-3-1, which could in principle be overwritten with alternative sup-tRNA sequences using prime editing^34^. In parallel, we explored how downstream termination sequences might affect tRNA expression levels. We identified four potential downstream sequences to test: the endogenous 100-bp sequence downstream of each mature tRNA sequence, and terminators consisting of TTT (3T), TTTT (4T) or TTTTT (5T), which are predicted to terminate transcription from a Pol III promoter with efficiencies of approximately 0%, 75% or 99%, respectively^35^.

We generated a lentiviral library containing all 6 leader sequences paired with 418 human tRNAs with their anticodons switched to CUA, UCA or UUA. We also assessed the effect of each of the 4 tested terminators placed downstream of sup-tRNAs paired with their endogenous leader sequences, with a total of 11,543 combinations of leader, sup-tRNA and terminator sequences (Supplementary Fig. 2 and Supplementary Table 5). We transduced each reporter cell line with the lentiviral library, isolated GFP-positive cells and sequenced the enriched tRNA variants. Unlike the lentiviral screening with exogenous promoters, this screen successfully identified at least one variant of all TAG sup-tRNAs that had been identified in the prime editing screen. The most effective sup-tRNAs performed best when paired with their own endogenous leader sequence (Extended Data Fig. 4f). Thus leader sequences have a critical regulatory role in tRNA activity.

The screen results also indicated that a terminator is essential for sup-tRNA activity, with a directly adjacent 5T terminator outperforming most endogenous terminator sequences (Extended Data Fig. 4g). We hypothesize that the absence of a terminator may lead to run-off of Pol III, or weakened expression due to a lack of the traditional Pol III recycling mechanism in which Pol III is rapidly reused for a new round of transcription after completing a transcript^36^. Notably, only 93% and 48% of natural downstream sequences have a 4T or 5T termination sequence, respectively, within 100 bp of their mature tRNA sequence^37^ (Supplementary Fig. 8a,b).

## Saturation mutagenesis of sup-tRNAs

We next hypothesized that introducing mutations within the tRNA itself could further enhance sup-tRNA activity. We also sought to identify silent mutations that could improve prime editing outcomes by evading cellular mismatch repair (MMR)^38^ and by preventing rebinding of the prime editor to the target tRNA locus following repair. We selected three candidate sup-tRNAs (tRNA-Arg-CCT-4-1, tRNA-Tyr-GTA-2-1 and tRNA-Leu-TAA-4-1) and a corresponding mouse sup-tRNA (tRNA-Leu-TAA-2-1) and generated lentiviral libraries that include every possible 1-bp deletion, single-nucleotide substitution variant (SNV), and simultaneous base pair substitution in each tRNA’s paired bases (Supplementary Fig. 2 and Supplementary Table 6).

**Extended Data Fig. 5:**
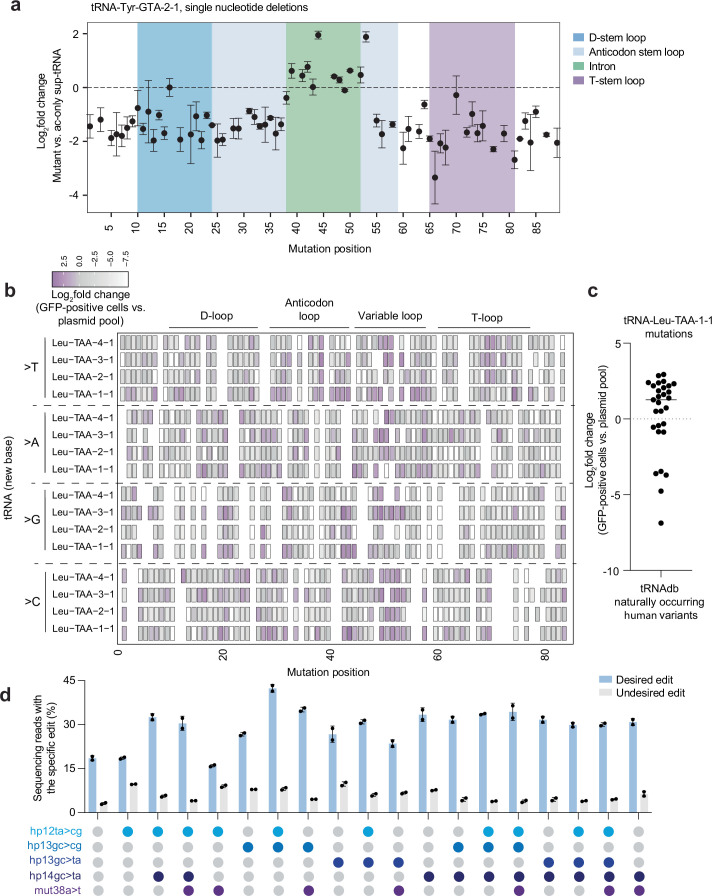
Engineering the sequence of sup-tRNAs. (**a**) Log_2_ fold change in abundance among GFP-positive cells of mutant sup-tRNAs compared to an ac-only sup-tRNA for tRNA-Tyr-GTA-2-1 with its anticodon changed to CUA. Mutation position is indicated on the x-axis. (**b**) Heatmap representing log_2_ fold change in abundance of mutant sup-tRNAs among GFP-positive cells compared to the plasmid pool for each single-nucleotide variant (SNV) in the four Leu-TAA sup-tRNA genes. Mutation position is indicated on the x-axis. (**c**) Log_2_ fold change in abundance of mutant sup-tRNAs among GFP-positive cells compared to the plasmid pool for all naturally occurring human variants of tRNA-Leu-TAA-1-1 documented in tRNAdb. (**d**) Sequencing reads with the specified edit (%) for epegRNAs used to install each of the indicated combinations of mutations in the endogenous tRNA-Leu-TAA-1-1 gene alongside changing its anticodon to CUA. Values and error bars reflect mean±s.d. of n = 2 independent biological replicates.

We transduced the reporter cell line with these lentiviral libraries and compared their performance to that of the corresponding anticodon-only (ac-only) sup-tRNA sequences. Most mutations reduced sup-tRNA performance (Extended Data Fig. 4h). Deletions were poorly tolerated across sup-tRNAs (Extended Data Fig. 5a). By contrast, for each sup-tRNA, a subset of SNVs and paired-base substitutions improved sup-tRNA performance (Extended Data Fig. 4h). We also found that beneficial mutations in tRNA-Leu-TAA-4-1 could apply to the related family members tRNA-Leu-TAA-1-1, tRNA-Leu-TAA-2-1, and tRNA-Leu-TAA-3-1 (Extended Data Fig. 5b, Supplementary Fig. 9 and Supplementary Table 7).

## Engineering an optimal Leu-TAA sup-tRNA

**Fig. 2:**
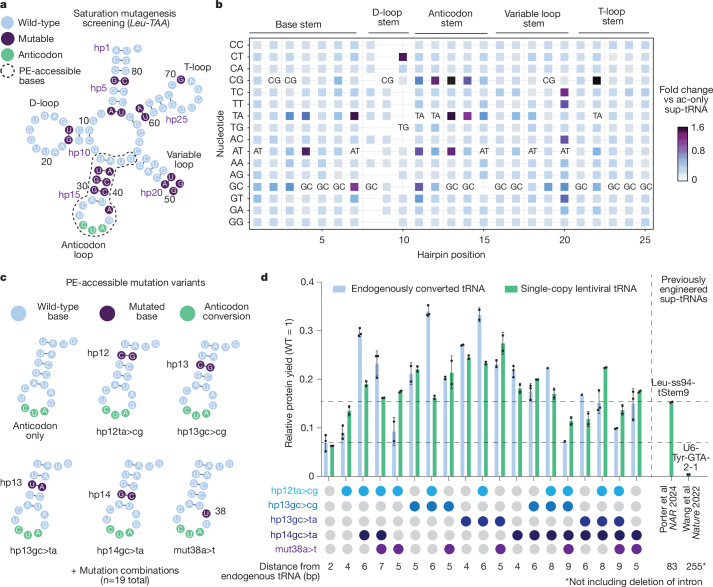
Identifying prime editing-accessible sup-tRNA mutation combinations. **a**, Results of saturation mutagenesis of tRNA-Leu-TAA-4-1, with mutations that are comparable or better than an ac-only sup-tRNA shown in purple. Bases that would easily be accessible with prime editing (PE-accessible bases) at the same time as changing the anticodon are circled with a dashed line. In this representation, each hairpin dinucleotide is represented by a number (hp1–hp25). **b**, Heat map showing the relative fold change in activity of hairpin mutations compared with the ac-only sup-tRNA. Hairpin positions correspond with hp1–hp25 in **a**. **c**, Sequences of the anticodon stem loops of five prime editing-accessible mutation variants and an ac-only control, from which 19 variant combinations were generated. **d**, Protein yield relative to wild type (WT) after readthrough with an endogenously converted tRNA-Leu-TAA-1-1 sup-tRNA, a single-copy lentiviral tRNA-Leu-TAA-1-1 sup-tRNA with the indicated mutations, or with alternative engineered sup-tRNAs (described in Porter et al.^32^ and Wang et al.^5^). The Leu-ss94-tStem9 variant is the engineered mature tRNA sequence converted to a TAG suppressor, flanked by the immediate 55-bp leader sequence of the tRNA-Cys-GCA-12-1 gene and the 35-bp trailer sequence of the tRNA-Ile-TAT-1-1 gene. The number of bases from the nearest endogenous tRNA are indicated. Data are mean ± s.d. of *n* = 2 independent biological replicates.

Since the tRNA-Leu-TAA family exhibited the highest baseline suppression activity, we selected this sup-tRNA backbone for further engineering (Fig. 2a,b). We focused on the mutations that were capable of being introduced at the same time as anticodon conversion with prime editing. Since mutations in sup-tRNAs can have additive effects^4,32^, we generated 19 combinations of the 5 anticodon-proximal mutations and introduced them into the top-performing tRNA-Leu-TAA-1-1 sup-tRNA using either prime editing at the endogenous locus or by expressing the variant from a single-copy lentiviral construct (Fig. 2c). For clarity, we refer to tRNAs whose anticodon alone was replaced as ac-only sup-tRNAs. When additional mutations were introduced to enhance readthrough, we designate them engineered sup-tRNAs and specify the type of mutation introduced; for example, engineered sup-tRNA (tRNA-Leu-TAA-1-1+hp12ta>cg+hp13gc>cg).

Of note, we observed that sup-tRNA mutations can be additively combined, supporting the production of up to fivefold higher levels of full-length GFP protein than the ac-only sup-tRNA, yielding up to 35% of the amount of GFP as control cells expressing an uninterrupted GFP gene (Fig. 2d, Extended Data Fig. 5d and Supplementary Table 8). Engineered sup-tRNAs developed in this study substantially outperform previously engineered sup-tRNAs, including a recently reported variant that yielded 16% GFP protein yield (Fig. 2d) but would require changing 83 bp of a native tRNA sequence for genomic installation^32^. By contrast, the best-performing variants we engineered require the substitution of only 6 bp from the wild-type tRNA-Leu-TAA-1-1 gene sequence.

**Extended Data Fig. 6:**
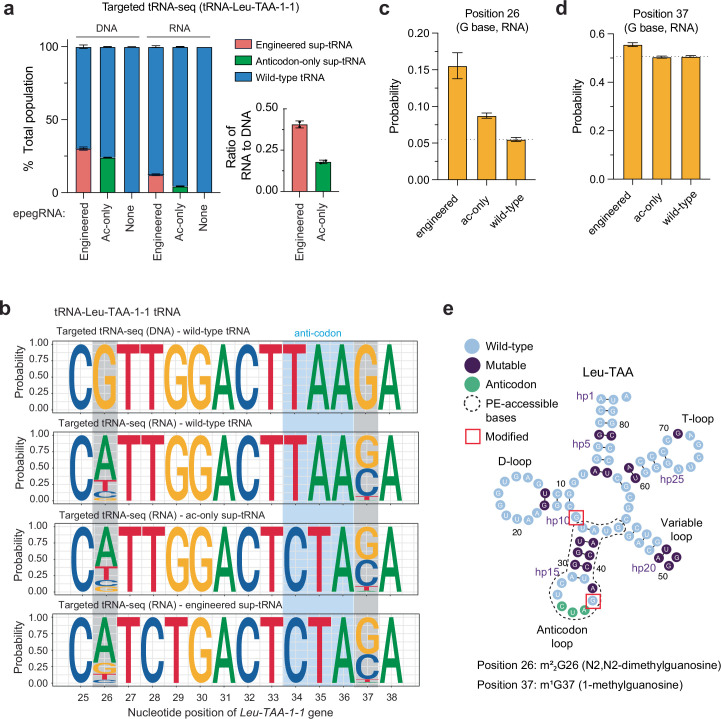
Abundance levels of PE-installed sup-tRNAs compared to wild-type tRNAs. (**a**) Targeted tRNA-seq of tRNA-Leu-TAA-1-1 measures the abundance of engineered sup-tRNA, ac-only sup-tRNA, or wild-type tRNA at the DNA and RNA levels. (**b**) Sequence logo of targeted tRNA-seq for reads corresponding to the wild-type tRNA, ac-only sup-tRNA, and engineered sup-tRNA at the RNA and DNA levels. (**c-d**) Probability of a G base at position 26 (c) or a G base at position 37 (d) for reads corresponding to the engineered sup-tRNA, ac-only sup-tRNA, or wild-type tRNA. (**e**) Schematic of a Leu-TAA tRNA and the position of its modified bases. The Induro RT used for cDNA generation will result in an “N” nucleotide at any position that is modified in the RNA.

To evaluate the cellular abundance of prime editing-installed sup-tRNAs compared with a wild-type endogenous tRNA, we performed targeted tRNA sequencing for tRNA-Leu-TAA-1-1 on a polyclonal edited population. By comparing the installation efficiency of each sup-tRNA at the DNA level with the frequency of corresponding tRNA-sequencing reads at the RNA level, we inferred the relative abundance of prime editing-installed sup-tRNAs versus the wild-type tRNA at the same genomic locus. We observed that the ac-only sup-tRNA and the engineered sup-tRNA are 5.6-fold and 2.5-fold less abundant, respectively, than the endogenous tRNA-Leu-TAA-1-1 that they replace (Extended Data Fig. 6a). Introducing mutations in the anticodon stem thus increased the abundance of the engineered sup-tRNA compared with the ac-only sup-tRNA (Extended Data Fig. 6a). Both sup-tRNAs were modified at positions 26 and 37, as expected^39^ (Extended Data Fig. 6b–e). These findings suggest that mutations in the anticodon stem can increase engineered sup-tRNA performance in part by increasing its cellular abundance.

## Optimization of sup-tRNA installation

**Fig. 3:**
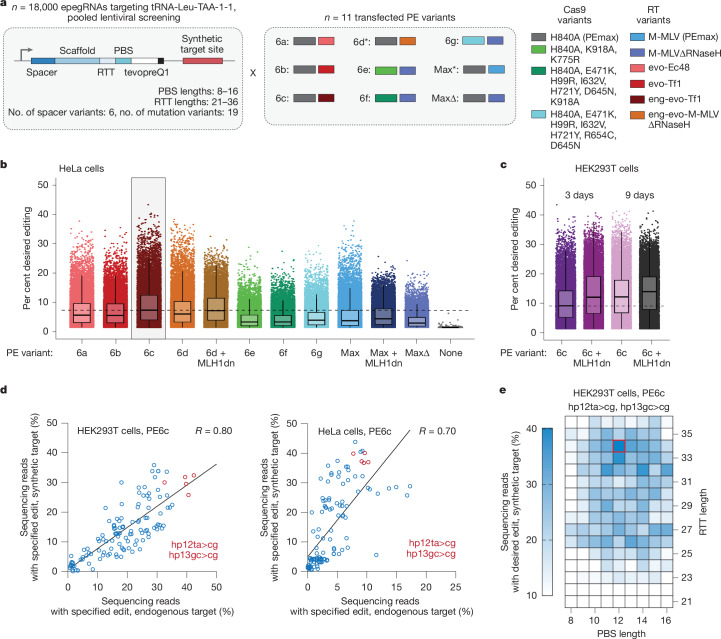
Optimization of a prime editing strategy for engineered sup-tRNA installation. **a**, Pooled screening strategy and prime editing system variants. Asterisk indicates presence of MLH1dn. **b**,**c**, Per cent desired editing at a synthetic target site for each of the indicated prime editors in HeLa cells (**b**) and HEK293T cells (**c**). **d**, Per cent editing at a synthetic target site in the pooled screen plotted against the per cent editing at the endogenous locus in arrayed validation in HEK293T cells (left) and HeLa cells (right) for the top 3 and bottom 3 epegRNA architectures per mutation variant. Pearson correlation is indicated in the top right. **e**, Example of prime editing efficiencies at a synthetic target site across PBS length and RTT length combinations for installing the hp12ta>cg+hp13gc>cg engineered sup-tRNA.

Encouraged by the greatly enhanced suppression efficiency achieved through iterative engineering of the mature tRNA sequence, we next optimized prime editing strategies to convert the mammalian endogenous tRNA-Leu-TAA-1-1 tRNA gene into the ac-only sup-tRNA or engineered sup-tRNA. We designed a lentiviral epegRNA library of 17,280 epegRNAs that exhaustively tested spacer variants, PBS lengths from 8 to 16 bp, RTT lengths from 21 to 36 bp, and combinations of each of the 19 mutation variants of interest alongside 720 control epegRNAs (Fig. 3a). We encoded a tRNA-Leu-TAA-1-1 synthetic target site directly adjacent to the lentiviral epegRNA to read out both the epegRNA and the outcome of editing in the same sequencing read, transfected cells transduced with this library with a panel of plasmids encoding prime editor proteins^40^ (PE6a, PE6b, PE6c, PE6d, PE6e, PE6f, PE6g, PEmax and PEmaxΔRNaseH) (Fig. 3a,b and Supplementary Tables 9 and 10), and evaluated editing at the synthetic target site using sequencing. Furthermore, we performed experiments in both MMR-deficient (HEK293T) cells and MMR-proficient (HeLa) cells and included conditions in which MMR was transiently inhibited by co-transfection of a dominant-negative MMR protein (MLH1dn)^38^.

**Extended Data Fig. 7:**
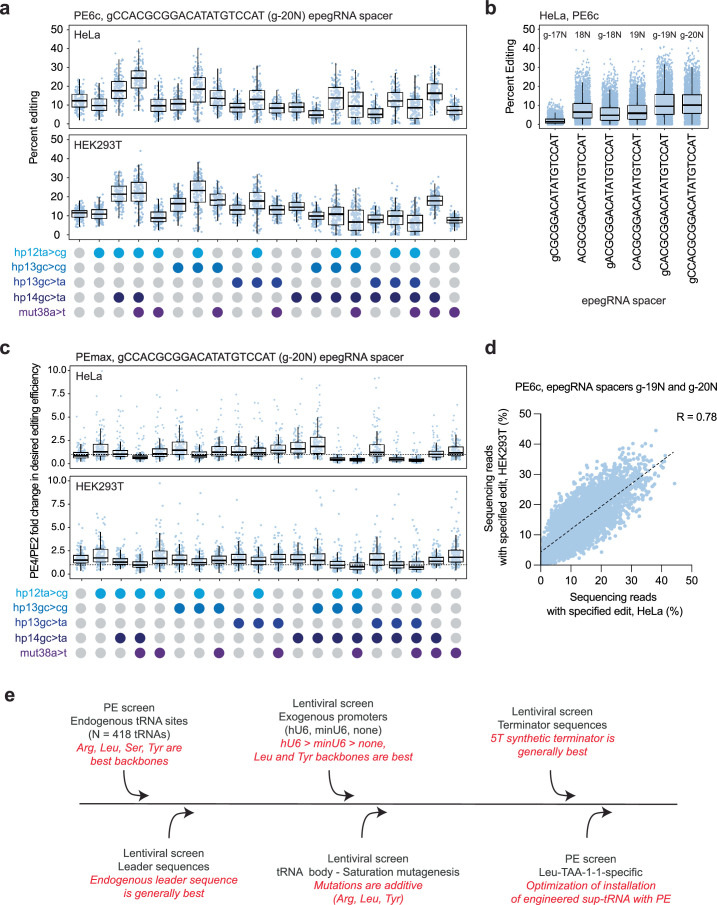
Optimization of a prime editing strategy for engineered sup-tRNA installation. (**a**) Percent editing efficiency across all epegRNAs with the indicated spacer from the PE6c screen in HeLa (top) and HEK293T (bottom) cells. Trends across the given mutation combinations are shown. (**b**) epegRNA spacer-dependent percent editing in HeLa cells transfected with the PE6c prime editor. Each point indicates a unique epegRNA sequence. (**c**) Fold change in desired editing efficiency when cells were transfected with MLH1dn and PEmax (PE4) compared to being transfected with PEmax alone (PE2) in HeLa (top) and HEK293T (bottom) cells. The tRNA mutations introduced during prime editing are shown below the graphs. (**d**) Sequencing reads with specified edit in HEK293Ts (y-axis) and HeLas (x-axis) transfected with the PE6c prime editor with the indicated epegRNA spacers. Pearson correlation is shown in the top right. (**e**) Schematic of improvements made across PE-installed sup-tRNAs, with a summary of the outcomes of each step shown in red.

Across all conditions tested, PE6c yielded the highest mean editing efficiency (Fig. 3b). The mutation combination of hp12ta>cg+hp14gc>ta+mut38a>t yielded the highest mean editing of 24% (Extended Data Fig. 7a) and the inclusion of MLH1dn resulted in greater mean editing efficiencies for most tested variants, suggesting that MMR evasion strategies can enhance editing efficiency for these edits (Fig. 3b and Extended Data Fig. 7c). We observed a high correlation (*R* = 0.78) between the results from the HeLa and HEK293T datasets (Extended Data Fig. 7d and Supplementary Discussion).

To validate that editing of the lentivirally integrated target site used in the screen reflects editing of the endogenous tRNA-Leu-TAA-1-1 locus, we performed arrayed validation on 122 high-performing and low-performing epegRNAs in HEK293T and HeLa cells. Editing efficiency at the endogenous locus and at the lentiviral target site in both cell types were correlated (Fig. 3d,e, *R* = 0.80 in HEK293T cells and *R* = 0.70 in HeLa cells), indicating that the lentiviral target-matched screen reflects prime editing outcomes at the endogenous locus.

On the basis of the optimized editing results and the readthrough efficacy data, we selected two variant combinations to advance: hp13gc>ta+hp12ta>cg and hp13gc>cg+hp12ta>cg. To finalize our editing strategy, we tested whether a PE3 or PE3b strategy—both of which use a nicking guide RNA (ngRNA) to nick the unedited strand of the heteroduplex editing intermediate in order to favour the use of the edited strand as a template for DNA repair—could further improve editing outcomes^3^. Top-performing PE3 strategies yielded mean editing efficiencies of ~60–80% in HEK293T cells, substantially outperforming earlier designs that yielded <8% editing and completing the optimization of the proof-of-concept editing strategy for PERT (Supplementary Fig. 8c and Extended Data Fig. 7e).

## Off-target editing analysis

**Extended Data Fig. 8:**
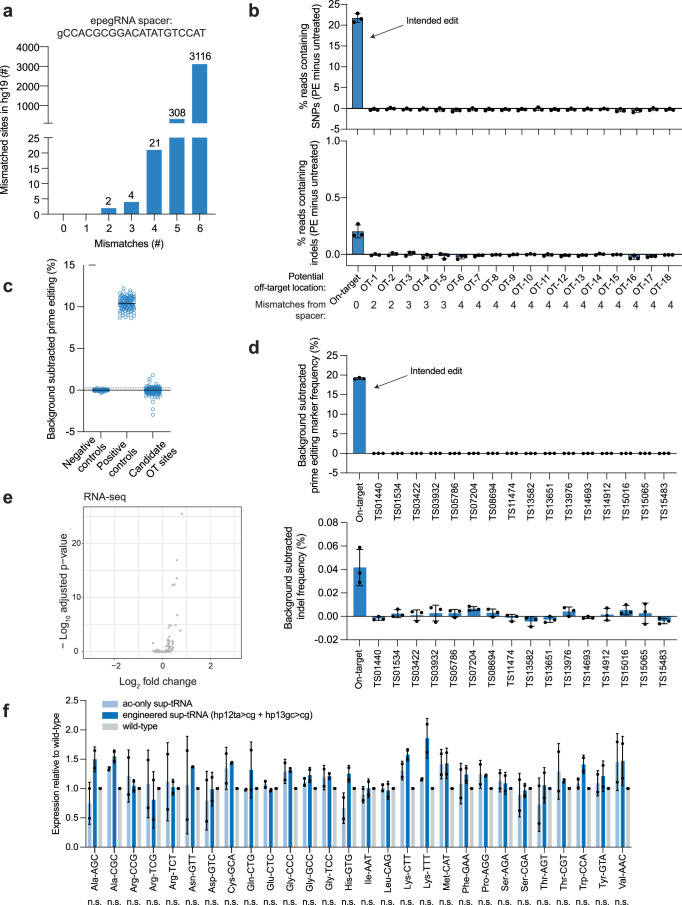
Off-target analysis for optimized epegRNA. (**a**) Computational analysis of number of sites in the hg19 genome with the indicated number of mismatches from our epegRNA spacer. (**b**) Percent of sequencing reads containing single nucleotide polymorphisms (SNPs) (top) or insertions or deletions (indels) (bottom) at each of the indicated locations in the genome, either on-target at the tRNA-Leu-TAA-1-1 locus or at 18 potential off-target sites. The number of mismatches from the spacer is indicated below. (**c**) Percent prime editing at each of the indicated target site types in the off-target screen after subtracting background frequencies observed in cells transfected with epegRNA only and no prime editor. (**d**) Percent prime editing marker frequency (top) or percent indel frequency after background subtraction (bottom) for individual validation of nominated off-targets from the off-target screen. (**e**) RNA-seq comparing cells transfected with PE6c and an epegRNA/ngRNA designed to convert tRNA-Leu-TAA-1-1 into a sup-tRNA compared to an unrelated epegRNA/ngRNA pair designed to edit the *HEK3* locus. Each data point represents the expression of a gene. No genes had an adjusted p-value < 0.05 and a |log_2_ fold change |>1. (**f**) Quantitative PCR analysis of the expression levels of 28 different tRNA families in cells expressing a PE-installed ac-only sup-tRNA, engineered sup-tRNA, or wild-type cells. n.s. = not significant.

We assessed off-target editing from PERT using two independent methods. First, we identified all sites in the human genome with up to four mismatches from the protospacer of the best-performing engineered sup-tRNA (Extended Data Fig. 8a). We sequenced at depth each of these sites following prime editing to install an engineered sup-tRNA (tRNA-Leu-TAA-1-1+hp12ta>cg+hp13gc>cg) and no significant off-target editing was observed above that of an untreated control (Extended Data Fig. 8b).

Second, as a genome-wide unbiased method for detecting potential off-target edits, we performed a screen in mammalian cells with a large pool of lentiviral integrated putative off-target sites. We computationally identified off-target sites in the genome with up to 6 mismatches from the optimized epegRNA’s protospacer or with up to 4 mismatches and up to two bulges, identifying 17,206 candidates (Supplementary Table 11). This coverage is identical to the computational filtering step of circularization for in vitro reporting of cleavage effects by sequencing (CIRCLE-seq)^41^, a commonly used genome-wide off-target detection method for RNA-guided nucleases. Following lentiviral integration of the pool of off-target candidate sites and transfection to install an engineered sup-tRNA (tRNA-Leu-TAA-1-1+hp12ta>cg+hp13gc>cg) with prime editing, we sequenced the lentiviral cassette and analysed editing at each off-target site.

This analysis nominated 16 candidate off-target sites with potential prime editing events occurring above the level of the untreated control sample in prime-edited cells (Extended Data Fig. 8c). To further investigate these potential off-target sites, we individually amplified the genomic region corresponding to each of the 16 nominated off-target sites in cells transfected with prime editing reagents to install an engineered sup-tRNA (tRNA-Leu-TAA-1-1+hp12ta>cg+hp13gc>cg). We observed no off-target prime editing or indel formation above background for all amplified genomic loci (Extended Data Fig. 8d). These data are consistent with previous reports of the high sequence specificity of prime editing, which probably arises from its mechanism requiring multiple independent DNA or RNA hybridization events in order to make a productive edit^3,42,43^.

## Effect of PERT on the transcriptome

We evaluated the potential for several types of abnormalities in human cells following conversion of an endogenous tRNA into a sup-tRNA. To detect transcriptome perturbations, we performed RNA-seq after converting the endogenous tRNA-Leu-TAA-1-1 gene into an engineered sup-tRNA (Leu-TAA-1-1+hp12ta>cg+hp13gc>cg) with prime editing. We observed no differentially expressed genes (FDR < 0.05 and |log_2_(fold change)| > 1), suggesting no evident stress response from converting the tRNA-Leu-TAA-1-1 tRNA gene into a sup-tRNA gene in a human cell line (Extended Data Fig. 8e).

Next, we evaluated the effect of repurposing the tRNA-Leu-TAA-1-1 tRNA gene on global tRNA homeostasis by performing quantitative PCR to measure the pooled expression of 28 different tRNA gene families following prime editing-mediated installation of an ac-only or engineered sup-tRNA (Leu-TAA-1-1+hp12ta>cg+hp13gc>cg). We observed no significant difference (adjusted *P *value > 0.05) in the expression of any of these 28 tRNA gene families in either treatment condition relative to wild-type cells (Extended Data Fig. 8f), suggesting that the tRNA-Leu-TAA-1-1 tRNA gene can be repurposed without significant perturbation to general tRNA homeostasis.

## Effect of PERT on the proteome

**Extended Data Fig. 9:**
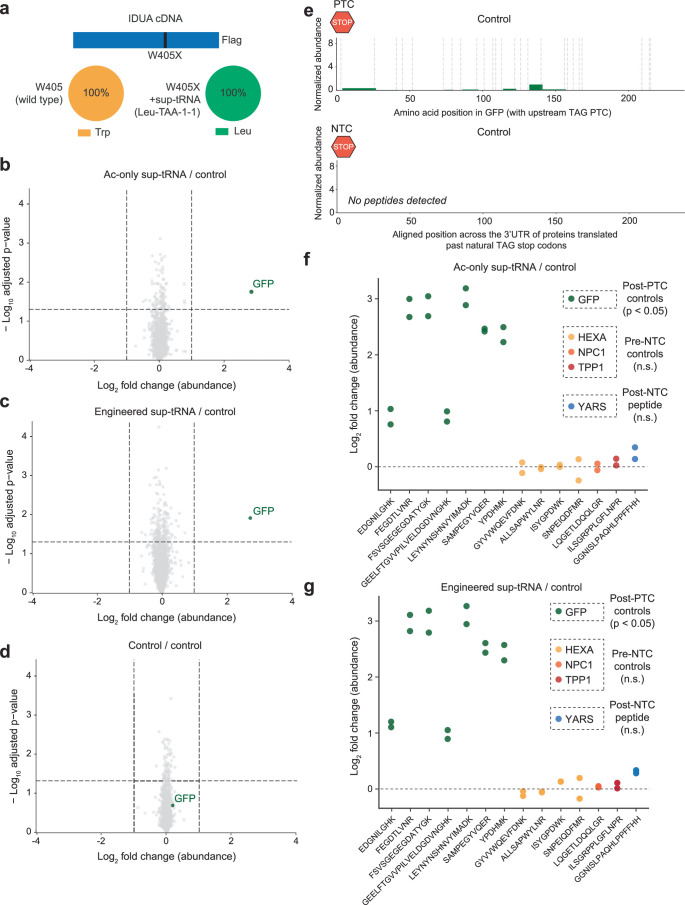
Mass spectrometry on PE-installed sup-tRNAs to evaluate readthrough of PTCs and NTCs. (**a**) Evaluation by mass spectrometry of the frequency of amino acid installation in a Flag-tagged murine IDUA cDNA at position 401 with a wild-type cDNA sequence (W401) or with a cDNA sequence containing a PTC (W401X) and with a PE-installed sup-tRNA sequence. (**b-d**) Protein-level analysis of whole proteome mass spectrometry data from cells prime edited to install an ac-only sup-tRNA (b), engineered sup-tRNA (c), or unedited control cells (d). Log_2_ fold change in protein abundance compared to unedited control cells. Dashed lines indicate an adjusted p-value of 0.05 on the y-axis and log_2_ fold change in abundance of ±1 compared to unedited control cells. (**e**) Normalized abundance of peptides identified past a premature termination codon (PTC) (top) or past natural TAG termination codons (NTCs) (bottom) for non-prime edited control HEK293T cells. Dotted lines correspond to trypsin cleavage sites. (**f-g**) Target-ion mass spectrometry showing change in abundance of peptides predicted to result from NTC readthrough of the 69 most abundant proteins that use a TAG stop codon in HEK293T cells expressing an ac-only sup-tRNA (f) or an engineered sup-tRNA (g) compared to non-prime-edited cells. All peptides identified post-PTC (GFP) are significant (adjusted p < 0.05). Peptides for pre-NTC controls and for one post-NTC peptide identified are not significant (adjusted p > 0.05). The data shown in f and g are only a subset of the complete data set, which is provided in Supplementary Table 14.

To evaluate potential readthrough of NTCs, we performed mass spectrometry to detect peptides that are translated into the 3′ UTR for protein-coding genes terminating with a TAG stop codon. After confirming that Leu-TAA sup-tRNAs retain 100% fidelity of leucine charging (Extended Data Fig. 9a), we computationally predicted all peptides that could be generated by reading through the 4,036 natural TAG termination codons and extending translation to the second in-frame stop codon. We then examined whether these predicted peptides were present by performing label-free quantitative mass spectrometry on trypsin-digested total protein lysates from both control and prime-edited HEK293T cells expressing and mCherry-STOP-GFP reporter and either the ac-only sup-tRNA or the engineered sup-tRNA (Leu-TAA-1-1+hp12ta>cg+hp13gc>cg).

**Fig. 4:**
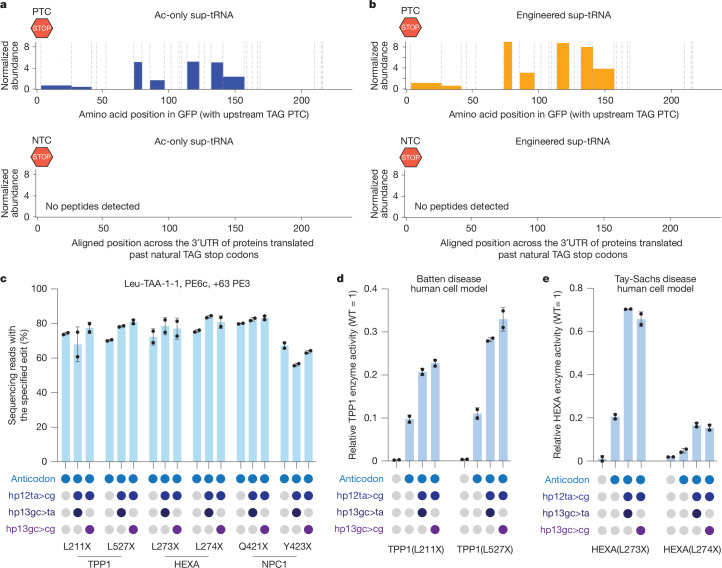
Prime editing-installed sup-tRNAs can rescue protein expression across diverse disease contexts. **a**,**b**, Normalized abundance of peptides identified past a PTC (top) or past natural TAG termination codons (NTCs) (bottom) for cells prime edited to express an ac-only sup-tRNA (**a**) or an engineered sup-tRNA (**b**). Dashed lines correspond to predicted potential trypsin cleavage sites. **c**, Desired editing outcomes in six HEK293T cell models of disease for each specified edit. **d**, TPP1 enzyme activity in treated and untreated human cell models of Batten disease relative to wild-type controls. **e**, HEXA enzyme activity in treated and untreated human cell models of Tay–Sachs disease relative to wild-type controls.

At the global protein abundance level, the only protein that was significantly (adjusted *P* value < 0.05, more than 2-fold change) different in abundance between control samples and prime-edited samples was the reporter GFP, which was 8.1-fold and 7.3-fold more abundant in cells edited with the ac-only sup-tRNA and the engineered sup-tRNA, respectively (Extended Data Fig. 9b–d and Supplementary Tables 12 and 13). Although we observed robust readthrough of the PTC and peptides generated for GFP, we detected no peptides from translation past the NTC for any TAG-terminated protein (Fig. 4a,b and Extended Data Fig. 9e).

To test with enhanced sensitivity the ability of a prime editing-installed sup-tRNA to induce NTC readthrough, we identified the 69 most abundant proteins that use a TAG stop codon from the above dataset and performed target-ion mass spectrometry to detect each of 111 predicted peptides that would be generated from reading past the NTC for these highly expressed proteins. We observed a single peptide corresponding to potential readthrough of an NTC (encoding 18 amino acids after the end of the tyrosyl-tRNA synthetase protein), but the abundance of this peptide was not significantly different (adjusted *P* value > 0.05) between untreated cells and cells that were prime edited to express a sup-tRNA (Extended Data Fig. 9f,g and Supplementary Table 14). Thus both proteome-wide and target-ion protein mass spectroscopy indicate that prime editing-installed sup-tRNAs do not broadly perturb the proteome or induce detected NTC readthrough under the conditions tested, probably owing to cellular mechanisms that safeguard against NTC readthrough.

## Rescue of therapeutically relevant PTCs

Several factors influence readthrough efficiency at a given PTC with a sup-tRNA, including the expression level of the PTC-containing transcript and the nucleotide context surrounding the PTC^44^. To assess the potential of PERT to rescue the effects of genomic PTCs with disease relevance, we evaluated readthrough across diverse, endogenously encoded PTCs. We generated models in HEK293T cells of known human pathogenic PTCs corresponding to a TAG stop codon in *TPP1*, *HEXA* and *NPC1* genes that cause Batten disease, Tay–Sachs disease and Niemann–Pick disease type C1, respectively. We chose six mutations that either originally encoded a leucine amino acid or are known to tolerate a leucine at the indicated position (*TPP1 *p.L211X, *TPP1 *p.L527X, *HEXA *p.L273X, *HEXA *p.L274X, *NPC1 *p.Q421X and *NPC1 *p.Y423X)^45^. As with many loss-of-function genetic diseases, previous studies suggest that even low levels of protein activity for these disorders can provide therapeutic benefits^46–48^.

**Extended Data Fig. 10:**
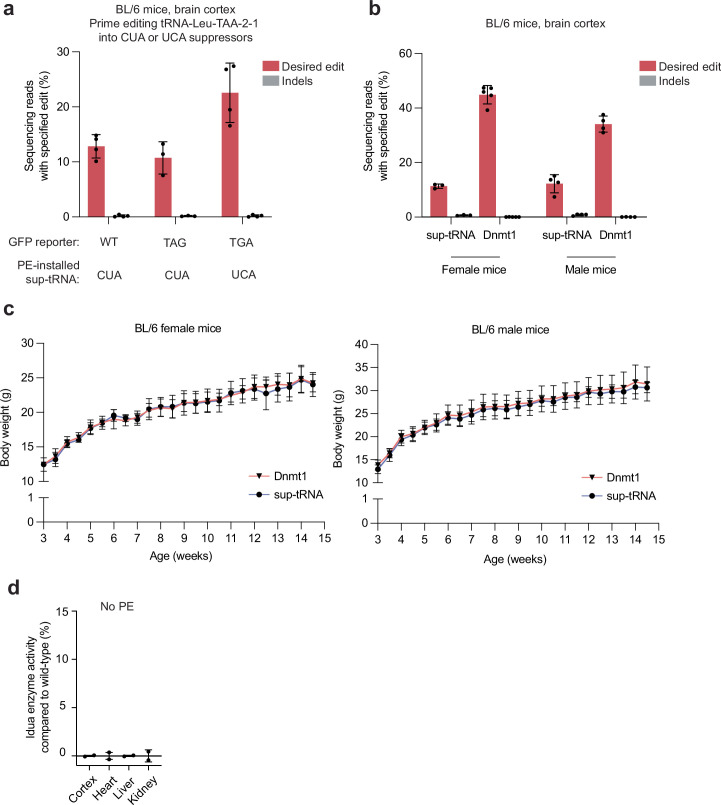
Prime editing-installed suppressor tRNAs that readthrough premature stop codons in GFP reporters in vivo. (**a**) Sequencing reads with the specified edit (%) for the brain cortex of BL/6 mice treated with prime editing agents to convert tRNA-Leu-TAA-2-1 into CUA or UCA suppressors alongside a wild-type GFP reporter (WT) or a GFP reporter containing a TAG or TGA stop codon. (**b**) Sequencing reads with the specified edit (%) for the brain cortex of BL/6 mice treated with prime ediing agents to convert tRNA-Leu-TAA-2-1 into a CUA suppressor or to introduce a benign Dnmt1 edit. (**c**) Body weight in grams (g) over time following treatment with prime editing agents, corresponding to editing shown in (b). N = 3 female sup-tRNA mice, 5 female Dnmt1 mice, 4 male sup-tRNA mice, 4 male Dnmt1 mice. (**d**) Idua enzymatic activity in Hurler syndrome mice without prime editing treatment. (**e**) Hematoxylin and eosin staining of the brain and liver. Arrows show vacuolated cells and inset is a high power magnification of a Purkinje cell. Scale bars are 20 µm and arrow heads are pointing to areas of marked vacuolization. (**f**) Tissue pathology score based on GAG storage evaluated by microscopy. Values and error bars reflect mean ± s.d.

We transfected these six cell models with the same optimized PE3 editing components to convert the endogenous genomic tRNA-Leu-TAA-1-1 locus into an ac-only sup-tRNA or engineered sup-tRNAs (tRNA-Leu-TAA-1-1+hp13gc>ta+hp12ta>cg or tRNA-Leu-TAA-1-1+hp13gc>cg+hp12ta>cg). At 3 d after transfection, mean editing efficiencies ranged from 67–80% for installation of the ac-only sup-tRNA, 56–84% for tRNA-Leu-TAA-1-1+hp13gc>ta+hp12ta>cg and 64–83% for tRNA-Leu-TAA-1-1+hp13gc>cg+hp12ta>cg (Fig. 4c, Supplementary Fig. 10a and Supplementary Fig. 12). Engineered sup-tRNA installation led to substantial (17–70%) restoration of enzyme activity in the four TPP1 and HEXA pathogenic PTC models, and partial restoration of full-length protein expression in both models of PTCs in *NPC1* (which encodes a cholesterol transporter rather than an enzyme) using the same prime editing reagents, establishing the disease-agnostic therapeutic potential of this approach (Fig. 4b–d, Extended Data Fig. 10b–d and Supplementary Fig. 13). In each case, treatment resulted in full-length protein expression that meets or exceeds the documented therapeutic threshold for each disease target^46–48^. Together, these results establish that a single prime editing agent to convert an endogenous genomic tRNA gene into a sup-tRNA can rescue a substantial fraction of enzyme activity and full-length protein production lost to nonsense mutations.

To evaluate the performance of PERT at a broad spectrum of nonsense mutations, we cloned 14,746 pathogenic PTCs in the ClinVar database flanked on either side by the 18 nucleotides present in the native mRNA sequence into a nonsense-mediated decay-sensitive reporter construct (Supplementary Fig. 11a,b, Supplementary Fig. 14 and Supplementary Table 15). Transcripts with PTCs are often destroyed by nonsense-mediated decay during the pioneer round of translation^49^; however, if the PTC is read through, the transcript will be stabilized for the remainder of its lifetime. This phenomenon allows quantification of the ability of a sup-tRNA to read through a given PTC by comparing the abundance of mRNA transcripts for each PTC-containing sequence (Methods). We transduced this library of PTC-containing sequences into a clonal ac-only sup-tRNA cell line and observed an average readthrough score of 69 ± 30% across 14,746 pathogenic PTCs, indicating stabilization of the mRNA transcript and readthrough of the PTC for most sequences. We independently confirmed readthrough of full-length *CFTR* cDNAs containing 15 distinct pathogenic PTC mutations that cause cystic fibrosis using a prime editing-installed ac-only sup-tRNA. Protein yields across the variants were moderately correlated (*R* = 0.49) with the readthrough scores measured in the pooled ClinVar screen (Supplementary Fig. 11c,d and Supplementary Table 16), with a maximum observed protein yield of 14% relative to wild-type CFTR. Together, these results demonstrate that PERT can effectively rescue diverse pathogenic genomic PTCs and can be used to generate therapeutically relevant levels of full-length protein and active enzymes in human cells.

## PERT in an in vivo reporter system

**Fig. 5:**
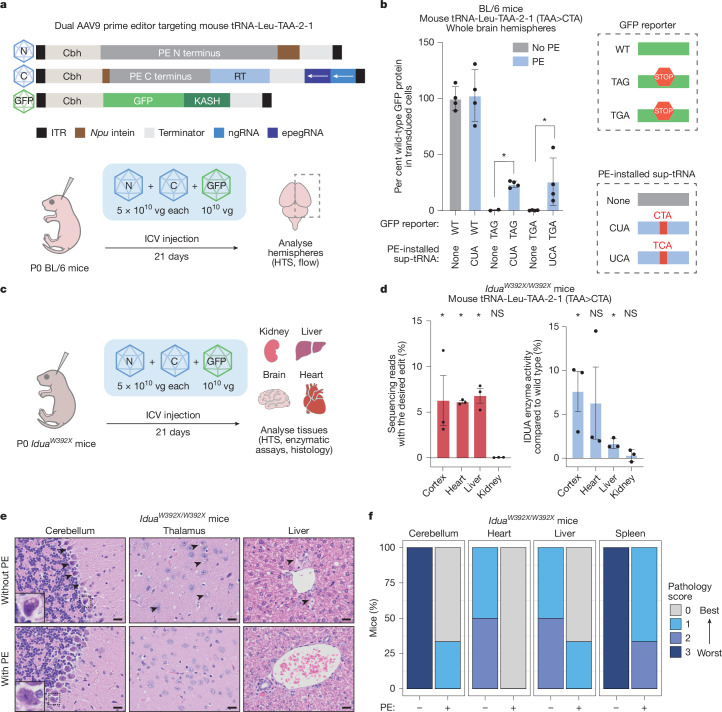
Prime editing generates functional sup-tRNAs to rescue animal models of disease. **a**, Schematic of experimental procedure for in vivo assessment of readthrough of exogenously supplied reporter constructs. HTS, high-throughput sequencing; ICV, intracerebroventricular; *Npu*, *Nostoc punctiforme*; vg, vector genomes; Cbh, chicken β-actin hybrid promoter; RT, reverse transcriptase domain; KASH, Klarsicht, ANC-1 and Syne homology domain; ITR, inverted terminal repeat. ** b**, In vivo readthrough efficacy of an exogenously supplied TAG or TGA premature stop codon reporter construct. *n* = 4 mice per condition. *P* values are calculated using a one-sided Welch’s *t*-test. **c**, Schematic of experimental procedure for in vivo treatment of the *Idua*^*W392X*^ mouse model. **d**, Left, desired editing efficiencies in treated homozygous *Idua*^*W392X*^ mice. Right, IDUA enzyme activity in treated homozygous *Idua*^*W392X*^ mice. *n* = 3 mice per condition. *P* values are calculated using a one-sided Welch’s *t*-test compared with non-prime edited mice (Extended Data Fig. 10d). *P < 0.05; NS, not significant (*P* ≥ 0.05). **e**, Hematoxylin and eosin staining of the brain and liver. Arrowheads indicate areas of considerable vacuolization; insets (bottom left corner of far left images) are high-power magnification of a Purkinje cell. Scale bars, 20 μm. **f**, Tissue pathology score based on GAG storage evaluated by microscopy. Drawings in **a**,**c** created in BioRender. Morrison, M. (2025) https://BioRender.com/zm7zlf4. Source Data

Next, we sought to evaluate the feasibility of applying PERT to rescue full-length protein production in vivo. We optimized prime editing agents to target mouse tRNA-Leu-TAA-2-1, the mouse equivalent of human tRNA-Leu-TAA-1-1, to first generate an ac-only sup-tRNA (mouse tRNA-Leu-TAA-2-1) (Supplementary Fig. 15a–f, Supplementary Discussion and Methods). We packaged prime editing agents to install the ac-only sup-tRNA using the v3em dual-AAV9 architecture^50^. We then designed an AAV9 eGFP reporter cassette containing a premature stop codon that only yields fluorescence when read through by a sup-tRNA. We administered these three AAV9 vectors, two for prime editing and one for the eGFP reporter, via intracerebroventricular injection into wild-type C57/BL6 mice on postnatal day 0 (P0) and evaluated prime editing and readthrough efficiency (Fig. 5a). Whereas all sup-tRNA engineering was done with a TAG sup-tRNA, for this experiment we additionally tested the installation of an analogous TGA sup-tRNA with a TGA-containing eGFP reporter construct.

At 3 weeks after injection, we collected the brain tissue for analysis of editing and readthrough efficiency. We observed an average editing efficiency of 11% for the TAG sup-tRNA and 23% for the TGA sup-tRNA in the bulk brain cortex of treated mice (Extended Data Fig. 10a). To measure readthrough efficacy, we dissociated the cells from whole hemisphere halves of treated mice and measured GFP expression by flow cytometry. We observed an average relative protein yield of 24% of the co-delivered TAG GFP reporter and 26% of the co-delivered TGA GFP reporter (Fig. 5b). Therefore, although editing efficiency would benefit from further optimization in mice, cells that receive prime editing agents can achieve readthrough levels that could ameliorate many diseases.

To assess the tolerability of PERT in vivo, we performed whole-proteome mass spectrometry on tissues that had been prime edited to express an ac-only sup-tRNA. Similar to our observations in human cell lines (Fig. 4a,b), we did not detect any peptides corresponding to readthrough of natural stop codons in tissues from mice treated with PERT (Supplementary Table 17). In addition, we monitored a separate cohort of mice over the course of 15 weeks and observed no significant difference in body weight in the PERT-treated mice (*n* = 7) relative to untreated controls (*n* = 9) (Extended Data Fig. 10b,c).

## PERT in an in vivo Hurler syndrome model

Finally, we investigated the in vivo therapeutic application of PERT in a mouse model of the human lysosomal storage disease mucopolysaccharidosis type I (MPS-I). MPS-1 is caused by a lack of α-L-iduronidase, leading to the pathogenic accumulation of glycosaminoglycans (GAGs). This model harbours a TAG stop codon, *Idua* p.W392X, which is orthologous to the most common pathogenic human mutation, *IDUA* p.W402X, and has been used previously to evaluate PTC readthrough in vivo^5,51^. For this experiment we further engineered the mouse sup-tRNA using mutations identified via saturation mutagenesis screening of mouse tRNA-Leu-TAA-2-1, generating an engineered sup-tRNA (mouse tRNA-Leu-TAA-2-1+hp13gc>ta+mut38a>t) (Supplementary Fig. 15 and Supplementary Discussion).

We packaged prime editing agents to install this sup-tRNA into the v3em dual-AAV9 architecture^50^ and treated homozygous and heterozygous P0 neonatal *Idua*^*W392X*^ littermates via intracerebroventricular injection (Fig. 5c). At 7 weeks after treatment, we evaluated prime editing efficiencies across tissues and observed mean desired prime editing efficiencies of 6.3%, 6.1%, 6.8% and 0.05% in the cortex, heart, liver and kidney, respectively (Fig. 5d), consistent with the known tissue tropism of AAV9 (ref. ^52^). Editing led to marked restoration of IDUA enzymatic activity compared with untreated controls, with mean enzymatic activity relative to wild-type mice of 7.6%, 6.3%, 1.7% and 0.31% in the heart, cortex, liver and kidney, respectively (Fig. 5d and Extended Data Fig. 10d). These data demonstrate the ability of in vivo PERT to restore enzyme function that has been abrogated by pathogenic endogenous PTCs.

To assess whether restoration in enzymatic activity was sufficient to rescue disease phenotypes, we performed histological analysis of brain, liver and spleen tissue from homozygous *Idua*^*W392X*^ mice. We scored phenotypic severity on the basis of MPS-I related pathology, including hepatic and splenic foam cell accumulation, heart vessel-associated foam cell accumulation, cerebellar Purkinje cell vacuolization, splenic stromal vacuolization and pathogenic GAG accumulation.

MPS-I GAG storage pathology was observed across all tissues analysed from untreated *Idua*^*W392X*^ homozygous mice, but was minimal or absent in treated counterparts. Purkinje cells of the cerebellum and thalamic neurons displayed moderate vacuolization in untreated homozygous *Idua*^*W392X*^ mice and was resolved following PERT treatment with absent or minimal vacuoles (Fig. 5e,f and Supplementary Note). We observed a similar pattern of attenuated vacuolization in the spleen, liver and heart in treated cohorts (Fig. 5e,f). Pathogenic accumulation of foam cells observed in the liver, heart and spleen in untreated mice was absent in the treated cohort (Fig. 5e,f). Finally, the liver of untreated mice displayed mild Alcian blue staining that was minimal in the treated mice, indicating depletion of GAG accumulation (Fig. 5f). These data are consistent with previous reports that restoration of only 1% or more IDUA enzyme activity is sufficient to rescue most or all observed MPS-1 disease phenotypes^5^. Together, these results illustrate the in vivo applicability of PERT for PTC readthrough and rescue of disease phenotypes in a clinically relevant mouse model.

## Discussion

Clinical genome-editing technologies, including nucleases, base editors and prime editors, have advanced the treatment of many genetic disorders. These therapeutic approaches have predominantly been disease-specific, with most efforts requiring the design of distinct gene-correction strategies for each pathogenic mutation. Given that more than 200,000 distinct mutations collectively affect hundreds of millions of individuals, developing, manufacturing and meeting regulatory requirements for thousands of individual therapies needed to treat even a modest fraction of patients with genetic disease is a monumental challenge, creating an urgent need for allele- and disease-agnostic therapeutic approaches that can address large classes of mutations with a single therapeutic composition.

As one potential disease-agnostic strategy, we developed PERT as a prime editing-mediated approach to convert redundant endogenous tRNAs into optimized sup-tRNAs. This strategy combines the durable, one-time treatment potential of genome editing with the broad applicability of sup-tRNAs. Through high-throughput evaluation of thousands of variants spanning all 418 high-confidence tRNA genes, we identified sup-tRNAs that are capable of reading through 32% of all possible nonsense mutations with precise amino acid correction (installing the wild-type amino acid in response to the PTC), including Arg and Leu sup-tRNAs that can read through TGA stop codons and Leu, Ser and Tyr sup-tRNAs that can read through TAG stop codons. Whereas precise amino acid correction is the most conservative strategy, many nonsense mutations may still be rescued with non-wild-type amino acid substitutions, although characterization of the missense protein variant in these cases is necessary^53^.

Among the sup-tRNAs tested, tRNA-Leu-TAA-1-1 exhibited the highest baseline readthrough efficiency. By swapping the anticodon of the endogenous tRNA-Leu-TAA-1-1 gene to CTA and introducing prime editing-accessible mutations in the anticodon stem, we engineered sup-tRNAs variants capable of restoring more than 35% of wild-type protein activity and/or expression from a gene containing a TAG nonsense mutation, surpassing the therapeutic threshold known for many loss-of-function genetic diseases^46–48^. In mouse models, including those for Hurler syndrome, PERT treatment demonstrated substantial protein restoration and near-complete rescue of disease pathology.

During the course of this study, we did not observe any evident toxicity from PERT in any cultured cells or mice, changes in the transcriptome of cells, including the abundance of other tRNAs, readthrough of natural stop codons in human cells or in tissues from PERT-treated mice using proteome-wide mass spectrometry, or any off-target editing using two genome-wide detection methods. Since the stoichiometry of cellular tRNAs can influence global translation outcomes^22–24^, we hypothesize that conversion of a single endogenous genomic tRNA gene into a sup-tRNA may be less perturbative to global translation outcomes than previous approaches to introduce sup-tRNAs by exogenous overexpression or delivery^4,5^.

Not all endogenous tRNA genes could be converted into effective sup-tRNAs with a simple anticodon change. In some cases, only specific isodecoders within a family were functional sup-tRNAs (for example, Tyr-GTA but not Tyr-ATA). Some sup-tRNAs favoured TAG stop codon readthrough (for example, Tyr-GTA), whereas others supported readthrough of only TGA stop codons (for example, Arg-TCG). We were unable to identify an effective prime editing-installed sup-tRNA for TAA stop codons with low-copy screening, although future efforts could explore reporter sensitivity adjustments or additional tRNA mutations in order to achieve this goal. Although certain patterns emerge in these data—such as the identification of Ser and Leu sup-tRNA backbones, which do not rely on the anticodon loop for recognition by their aminoacyl-tRNA synthetases^54,55^—it remains unclear why a functional Ala sup-tRNA, which also is thought to lack synthetase recognition of the anticodon, was not identified. Our data suggest that the determinants of sup-tRNA effectiveness, despite extensive study, remain incompletely understood.

Efficiency of sup-tRNA readthrough also depends on the sequence context of the nonsense mutation. Across the sequence contexts of 14,746 pathogenic TAG stop codons in ClinVar, more than 70% showed readthrough from PERT. However, additional factors outside of the immediate sequence context are also likely to influence sup-tRNA activity, including ribosome velocity and PTC position in the mRNA. Further improvements may come from mutations beyond the anticodon stem, such as alterations in the D- or T-loops, which may enhance aminoacylation, transcription or ribosome accommodation of sup-tRNAs. Alternative methods for integrating a sup-tRNA sequence into the genome, such as twin prime editing (twinPE)^34^, eePASSIGE^56^, CASTs^57,58^ or methods based on homology-directed repair^58^, may facilitate the incorporation of more advanced combinations of mutations. Combining PERT with readthrough-promoting small molecules may further boost functional protein restoration. We also anticipate that, although our primary goal was to optimize a sup-tRNA specifically for one-time genomic installation, the best-performing variants we identified could improve readthrough efficiency when delivered through tRNA-supplementation approaches such as AAV- or LNP-mediated delivery of sup-tRNAs.

Additional studies are needed to investigate and optimize the safety, efficacy and ability of the strategy developed in this study to be delivered to relevant tissues through viral and non-viral in vivo delivery methods. Future work could explore tissue-targeted capsid engineering, receptor-specific LNP-mediated or engineered virus-like particle-mediated^59,60^ delivery, and regulated or transient expression systems that avoid unnecessary long-term exposure to editing agents. Nevertheless, the findings of this initial study highlight the potential of PERT as a disease-agnostic therapeutic strategy in which a single composition of matter may offer a one-time treatment that benefits many different cohorts of patients with diverse genetic diseases. For example, approximately 8,000 people with cystic fibrosis^61^, 252,000 people with Stargardt disease^62^, 31,000 people with phenylketonuria^63^ and 43,500 people with Duchenne muscular dystrophy^64^ have nonsense mutations in *CFTR*, *ABCA4*, *PAH* or *DMD*, respectively, that could in principle be treated with common PERT prime editing agents after further optimization.

Although direct DNA-level correction of pathogenic mutations remains the most straightforward approach to precision genetic medicine, the large effort currently required to develop, meet regulatory requirements and manufacture many gene editing agents needed to correct many mutations underscores the importance of complementary allele-agnostic and disease-agnostic approaches such as PERT to maximize the ability of patients to benefit from advances in gene editing. By enabling a single treatment to address a broad cross-section of nonsense mutation-related diseases, PERT represents an advance toward making therapeutic genome editing accessible to a much larger patient population. We hope that PERT and the flexibility of prime editing will stimulate additional allele-agnostic and mutation-agnostic therapeutic gene editing strategies that may provide broadly applicable treatments for diverse serious disorders.

## Methods

### General methods

DNA amplification was conducted by PCR using Phusion U Green Multiplex PCR Master Mix (ThermoFisher Scientific) or Q5 Hot Start High-Fidelity 2× Master Mix (New England BioLabs) unless otherwise noted. DNA oligonucleotides were obtained from Integrated DNA Technologies. Plasmids expressing epegRNAs were constructed by Gibson assembly using a custom acceptor plasmid. Sequences of sgRNA and epegRNA constructs used in this work are listed in Supplementary Table 1. All vectors for mammalian cell experiments were purified using Plasmid Plus Midiprep kits (Qiagen) or PureYield plasmid miniprep kits (Promega), which include endotoxin removal steps. All experiments using live animals were approved by the Broad Institute Institutional and Animal Care and Use Committees. Wild-type C57BL/6 mice were obtained from Charles River (027).

### General mammalian cell culture conditions

Cell lines with homozygous PTCs in *TPP1*, *HEXA *and *NPC1*were generated using prime editing. HEK293T (ATCC CRL-3216), Neuro-2a (ATCC CCL-131) and HeLa (CCL-2) cells were purchased from ATCC and cultured and passaged in Dulbecco’s Modified Eagle’s Medium (DMEM) plus GlutaMAX (ThermoFisher Scientific), supplemented with 10% (v/v) fetal bovine serum (Gibco, qualified). All cell types were incubated, maintained, and cultured at 37 °C with 5% CO_2_. Cell lines were authenticated by their respective suppliers and tested negative for mycoplasma.

### Generation of cell lines

HEK293T cells were seeded at 100,000 cells per well on 24-well plates (Corning). 16–24 h after seeding, cells were transfected at approximately 60% confluency with 600 ng, 200 ng and 60 ng of PEmax plasmid, epegRNA plasmid and ngRNA plasmid, respectively using Lipofectamine 3000 according to manufacturer’s instructions (Thermo Fisher Scientific). 4 d after transfection, single cell clones were isolated by limiting dilution cloning and expanded over a 2-week period. The resulting colonies were further expanded and genotyped by high-throughput sequencing of the targeted locus and those found to be homozygous for the expected edit were retained for downstream experiments.

### Lentiviral production

HEK293Ts (ATCC CRL-3216) were transfected with pMD2.G (Addgene #12259) and delta8.2 (Addgene Plasmid #8455) packaging plasmids alongside the appropriate lentiviral backbone using Lipofectamine 2000. Lentiviral backbone sequences used in this work are listed in Supplementary Table 1. Medium was changed 24 h after transfection. Virus-containing supernatant was collected and filtered through a 0.45-μM filter 48 h after transfection. Virus was used immediately or stored at 4 °C for up to 1 week before cell transduction.

### General high-throughput lentiviral screening protocol

All oligonucleotides for high-throughput screening were ordered from Twist Biosciences as single-stranded oligonucleotide pools. Oligonucleotide pools were amplified using Q5 Hot Start High-Fidelity 2X Master Mix (NEB M0494L) to create double-stranded inserts for isothermal assembly. Primers used are indicated in each specific screening section of the Methods. The minimum number of PCR cycles was used to amplify (typically between 11 and 13 cycles) and double-stranded inserts were checked for size and/or inappropriate products by TapeStation (Agilent). For each 20-μl reaction cloned into the pSEP0308, pSEP0309 or pSEP0310 lentiviral backbones with a 300-bp insert, 50 ng of the appropriate lentiviral backbone was assembled with 10 ng of the appropriate double-stranded insert using NEBuilder HiFi DNA Assembly Master Mix (NEB E2621L). Reactions were scaled according to the number of elements in the library. For each 1,000 elements, an additional 20-μl reaction was set up. Reactions were incubated at 50 °C for 2 h and then pooled and purified using the QIAquick PCR purification kit (Qiagen 28104) according to manufacturer’s instructions. Reaction products were eluted in a minimum of 20 μl ddH2O, but otherwise were eluted in 2.5 μl per original 20-μl reaction.

Isothermal assembly reactions were electroporated into NEB 10-beta Electrocompetent *Escherichia coli* (NEB C3020) and plated onto LB plates aiming for at least 1,000× coverage of each library element. 14 h later, colonies were scraped and prepared using Qiagen Plasmid Plus kits (Qiagen 12945 and 12963) according to manufacturer’s instructions. Pooled plasmids were transfected into HEK293Ts alongside lentiviral packaging plasmids using Lipofectamine 2000. Medium was changed 24 h after transfection. Virus-containing supernatant was collected and filtered through a 0.45-μM filter 48 h after transfection. Cells were transduced with virus aiming for a multiplicity of infection of 0.3. Cells were transduced to ensure ≥1,000× coverage of transduced cells per element of the library. 2 d after transduction, cells were passaged with 1 μg ml^−1^ puromycin for 3–4 days to enrich for transduced cells. For screens requiring FACS isolation of GFP-positive cells, we calculated approximate coverage by multiplying the per cent of GFP-positive cells by the number of elements in the library and aiming for 1,000× coverage of that number. For example, if 10% of cells were GFP-positive for a 500 element library, we sorted at least 50,000 cells per replicate. Cells were pelleted and flash frozen on dry ice. Genomic DNA was isolated using QIAamp DNA Micro kits (Qiagen 56304) or QIAamp Mini kits (Qiagen 51304) depending on the cell number isolated.

All genomic DNA for each replicate was input into an initial set of PCR reactions using Q5 Hot Start High-Fidelity 2X Master Mix (NEB M0494L) to amplify the integrated lentiviral cassette and to add sequencing adapters, with a maximum of 5 μg genomic DNA per 50-μl reaction. Primers used are indicated in each screening section and are also listed in Supplementary Table 1. PCR reactions were purified using the QIAquick PCR purification kit (Qiagen 28104) according to manufacturer’s instructions and 2 μl of PCR product was input into a second PCR reaction to add unique sample indices and flow cell adapters to each amplicon. Final PCR reactions were bead purified with a ratio of 0.7× using SPRI beads, quality controlled using a TapeStation (Agilent) and quantified using a Qubit prior to high-throughput sequencing on an Illumina MiSeq, Illumina NextSeq, or Element Biosciences AVITI instrument. Sequencing conditions and downstream analyses are specific to each screen and are indicated in each separate screening section.

### PE2 screening

To convert each endogenous tRNA into a sup-tRNA, we identified 418 high-confidence tRNA genes and identified the two closest 20-bp spacers with an NGG protospacer-adjacent motif upstream and downstream of the anticodon. We allowed prime editing to target multiple tRNAs in the case of identical mature tRNA sequences. We systematically varied the PBS lengths from 11 bp to 14 bp, and varied the RTT homology lengths 3′ of the last edited nucleotide from 6 bp to 10 bp, where feasible. We designed three prime editing screens, each with RTTs that replaced the anticodon of each endogenous tRNA with one of three sup-tRNA anticodons (CUA, UCA or UUA). To enhance prime editing efficiency, we appended a structured RNA motif, tevopreQ1, to the 3′ end of each pegRNA, creating epegRNAs^29^. In total, we designed 17,579 epegRNAs to convert endogenous tRNAs to sup-tRNAs for each of the three possible sup-tRNA anticodons. Each pool also included 420 control epegRNAs targeting serine and arginine tRNAs, swapping their anticodons for those of other serine and arginine anticodons, which are not expected to give signal in a readthrough-based screen (Supplementary Table 2). We included a unique barcode sequence 3′ of the polyT terminator sequence for each epegRNA to improve our ability to assign each sequencing read to the correct element. The final oligonucleotides had the following design: a common 5′ end for isothermal assembly (5′-tatcttgtggaaaggacgaaacacc-3′), a unique epegRNA sequence followed by a polyT for Pol III termination, an adjustable length linker used to ensure that each element of the library was the same length, a unique 25 bp barcode, and a common 3′ end for isothermal assembly (5′-ctcgagtactaggatccattaggcg-3′). Oligonucleotides were amplified using oSEP0114 and oSEP0115 prior to isothermal assembly into a linearized pSEP0308 with a hU6 promoter for driving expression of the epegRNAs. Final libraries for next generation sequencing were sequenced on an Illumina MiSeq instrument using oSEP0213 as a custom Read1 primer with 300 cycles. For analysis, the first 25 cycles of the read were matched exactly to the barcode for each element of the screening pool, allowing for no mismatches. Reads were normalized on the basis of sequencing depth and sorted samples were compared with the plasmid pool representation of each element. Oligonucleotides and results for this screen are listed in Supplementary Table 2.

### Lentiviral sup-tRNA screen

To evaluate a wide range of tRNA promoter variants in high-throughput, we took the 418 high-confidence tRNA sequences from the human genome and swapped their anticodon to CUA, UCA or UUA to target the stop codons TAG, TGA or TAA, respectively. A unique barcode was assigned to each tRNA variant to reduce the likelihood that sequencing errors would influence assignment of library members. In addition, a pre-integrated Nextera Read1 adapter was included so that Read1 sequences on an Illumina instrument will read straight into the highly complex barcode for defining clusters. We cloned this pool using isothermal assembly into three different lentiviral backbones to evaluate the effect of different upstream elements on sup-tRNA efficacy: pSEP0308, which has a hU6 promoter, pSEP0309, which has a minU6 promoter and pSEP0310, which contains no exogenous promoter. For analysis, the first 25 cycles of the read were matched exactly to the barcode for each element of the screening pool, allowing for no mismatches. Reads were normalized on the basis of sequencing depth and sorted samples were compared with the plasmid pool representation of each element. Oligonucleotides and results for this screen are listed in Supplementary Table 3.

### Leader and terminator sequence screening

For the initial leader sequence screen, the 40 bp upstream of each endogenous high-confidence tRNA in the human genome was determined and placed upstream of the mature sequence for tRNA-Leu-TAA-3-1 with its anticodon changed to CUA followed by a polyT termination sequence. As controls, the same leader sequences were placed upstream of the mature sequence for tRNA-Leu-TAA-3-1 with its native anticodon. Libraries were sequenced and exact matching was used for both the leader sequence and the anticodon region of the read to assign to the correct library element. Oligonucleotides and results for this screen are recorded in Supplementary Table 4.

To next screen leader sequences across a wide range of sup-tRNAs, we generated a lentiviral library containing six leader sequences (two top-performing, two bottom-performing and two random sequences) paired with 418 human tRNAs with their anticodons switched to CUA, UCA or UAA. We also assessed the effect of each terminator sequence when placed downstream of sup-tRNAs paired with their endogenous leader sequences, with a total of 11,543 combinations of leader sequence, sup-tRNA and terminator. We included a unique 20-bp barcode sequence 3′ of the terminator sequence for each tRNA to improve our ability to assign each sequencing read to the correct element. For analysis, the first 25 cycles of the read were matched exactly to the barcode for each element of the screening pool, allowing for no mismatches. For analysis, alignment was confirmed between tRNA elements and the 20-bp barcode region and then subsequently the 20-bp barcode was used to assign each read to the appropriate library element. Reads were normalized on the basis of sequencing depth and sorted samples were compared with the plasmid pool representation of each element. Oligonucleotides and results for this screen are recorded in Supplementary Table 5.

### Saturation mutagenesis screening

To design variants for saturation mutagenesis screening, every sequence containing a SNV, paired substitution at all hairpin positions, and 1-bp deletion was generated computationally. Then, 25 bp ends compatible with isothermal assembly were added to either side (see Supplementary Table 1) and a unique barcode was assigned to each tRNA variant to reduce the likelihood that sequencing errors would influence assignment of library members. In addition, a pre-integrated Nextera Read1 adapter was included so that Read1 sequences on an Illumina instrument will read straight into the highly complex barcode for defining clusters. Variants were ordered as a Twist oligonucleotide pool for each individual tRNA for tRNA-Leu-TAA-4-1, tRNA-Arg-CCT-4-1, mouse tRNA-Leu-TAA-2-1 and tRNA-Tyr-GTA-2-1 (sequences in Supplementary Table 6). A pooled library of all possible variants for tRNA-Leu-TAA-1-1, tRNA-Leu-TAA-2-1, tRNA-Leu-TAA-3-1, and tRNA-Leu-TAA-4-1 was ordered as a separate Leu-TAA focused library (sequences in Supplementary Table 7).

For the tRNA-Arg-CCT-4-1, mouse tRNA-Leu-TAA-2-1, and tRNA-Tyr-GTA-2-1 screens, Twist oligonucleotides contained a leader sequence element for each tRNA and they were cloned by isothermal assembly into the pSEP0310 lentiviral backbone with no exogenous Pol III promoter. For the tRNA-Leu-TAA-4-1 and Leu-TAA focused library, Twist oligonucleotides did not contain a leader sequence element for each tRNA and they were cloned by isothermal assembly into the pSEP0308 lentiviral backbone with an exogenous hU6 promoter driving expression of the tRNAs. Libraries were sequenced on an Illumina instrument with 26 cycles for Read1 covering the barcode region and 86 cycles for Read2 covering the tRNA region. For analysis, alignment was confirmed between tRNA elements and the 25-bp barcode region and then subsequently the 25-bp barcode was used to assign each read to the appropriate library element.

### epegRNA optimization with synthetic target-site screening

To optimize epegRNA architectures, we designed a lentiviral epegRNA library of 17,280 epegRNAs to test out five spacer variants, PBS lengths from 8 to 16 nt, RTT lengths from 21 to 36 nt, and combinations of each of the 19 mutation variants of interest alongside 720 control epegRNAs with RTTs that do not encode an anticodon edit. On the same oligonucleotide, we encoded an adjacent tRNA-Leu-TAA-1-1 synthetic target site directly adjacent to the lentiviral epegRNA to read out both the epegRNA and the outcome of editing in the same sequencing read. We transfected cells transduced with this library with a panel of prime editor proteins that included PEmax and several engineered PE6 variants^40^ listed in Supplementary Data Tables 9 and 10. We performed experiments in both MMR-deficient (HEK293T) cells and MMR-proficient (HeLa) cells and included conditions in each case in which MMR was transiently inhibited by co-transfection of a dominant-negative MMR protein (MLH1dn)^38^. For experiments in HeLa cells, we collected genomic DNA 3 d after transfection. For experiments in HEK293T cells, we collected genomic DNA at 3 d and 9 d after transfection. We then performed high-throughput sequencing of the lentivirally integrated cassette. For analysis, sequencing reads were initially demultiplexed into individual fastq files by first aligning each read to a corresponding member of the epegRNA library using bowtie2 (ref. ^65^). Next, each individual fastq file was then trimmed to only include the full-length synthetic target site. Editing outcomes for each epegRNA sequence were quantified using CRISPResso2. Oligonucleotides and results for this screen are recorded in Supplementary Data Tables 9 and 10.

### RNA-seq and transcriptomic analysis

HEK293T cells were transfected with our optimized epegRNA sequence and ngRNA alongside PE6c or with an unrelated epegRNA and ngRNA pair targeting the *HEK3* locus alongside PE6c (see Supplementary Table 1). RNA was extracted 6 d after transfection using the Qiagen RNeasy kit (Qiagen) according to manufacturer’s instructions. RNA-seq libraries were generated using the SMART-Seq mRNA LP kit (Takara Bio) according to manufacturer’s instructions and sequenced 2× 75-bp on an Element Biosciences AVITI instrument. Fastq reads were trimmed of adapter sequences using Trim Galore, aligned to the human genome using STAR, and differential expression analysis was performed using DESeq2 and custom R scripts.

### Measurement of tRNA abundance

Total RNA, including small RNAs, was isolated from prime edited cells using miRNeasy kits (Qiagen) according to manufacturer’s protocols. RNA was reversed transcribed with SuperScript IV (Thermo Fisher Scientific) using a primer specific to each tRNA gene family queried^66^ (Supplementary Table 1). Next, quantitative PCR was performed using Power SYBR Green PCR master mix (Thermo Fisher Scientific) and primers specific to each tRNA gene family queried (Supplementary Table 1). For tRNA-Leu-TAA-1-1, we performed targeted tRNA sequencing. Specifically, we measured the relative abundance of the desired tRNA edit versus unedited in a polyclonal edited population in both genomic DNA and total RNA. For targeted tRNA sequencing, we performed reverse transcription using a primer specific to tRNA-Leu-TAA-1-1 and the Induro RT enzyme that is tolerant to RNA modifications^39^.

### Sequence context screening

To generate a diverse PTC sequence context reporter library, 14,746 naturally occurring premature TAG stop codons in protein-coding genes were identified in ClinVar that were annotated as pathogenic, likely pathogenic, or of uncertain significance. Each TAG stop codon was flanked on either side by the 18 nucleotides present in the native mRNA sequence. Positive controls were generated that contained the same sequence context but a TTG Leucine codon instead of a TAG premature stop codon. For negative controls, 2,800 ‘redundant stop’ control library members were generated, which were a subset of the ClinVar variants and their TTG controls but with the codon following the TAG stop codon or TTG codon changed to a TAA stop codon followed by a +1 frameshift to prevent readthrough.

The pSEP0211 lentiviral backbone was first linearized using oSEP0163 and oSEP0164 and the oligonucleotide pool was amplified using oSEP0165 and oSEP0166 (Supplementary Table 1). These sequences were cloned into the pSEP0211 lentiviral backbone between mCherry and GFP via Gibson assembly. Two days following transduction, we extracted both mRNA and genomic DNA from the transduced cell population, generated cDNA by reverse transcription, and sequenced the integrated reporter construct from both the cDNA and genomic DNA samples. For analysis, we calculated an ‘RNA score’ metric by quantifying the frequency of each element in the cDNA and dividing that value by the frequency of that element in the genomic DNA. To control for the impact each unique sequence context might have on transcript expression level independent of readthrough activity, we defined a ‘readthrough score’ for each ClinVar PTC by dividing the RNA score of each variant by the RNA score of its corresponding no-premature-stop equivalent. Oligonucleotides and results for this screen are recorded in Supplementary Table 15.

### Off-target prime editing screening

To identify candidate off-target prime editing sites, we used Cas-OFFinder^67^ to identify all human genomic sequences with up to six mismatches, or up to four mismatches combined with up to two bulges relative to our optimized epegRNA spacer sequence. To account for target-site binding and potential annealing of the full-length RTT product, we extracted the local sequence surrounding these sites to include 3 bp upstream of the putative target site and 34 bp downstream. We included 100 uniquely barcoded positive-control sequences corresponding to the endogenous tRNA-Leu-TAA-1-1 site and 694 negative-control sequences that shared no homology with the epegRNA spacer sequence. These sequences were cloned into the pSEP0310 lentiviral backbone and transduced into HEK293T cells after lentiviral production. These cells were then transfected with an epegRNA expression plasmid alone as a negative control, or with a PE6c prime editor expression plasmid alongside the epegRNA expression plasmid. 3 d after transfection, genomic DNA was isolated and the lentiviral integrated target site was amplified for high-throughput sequencing. The amplified target-site was sequenced using 2× 115 paired-end high-throughput sequencing. For analysis, high-throughput sequencing reads were initially demultiplexed into individual fastq files by first aligning each read to a corresponding member of the target-site library using bowtie2 (ref. ^65^). Next, each individual fastq file was then trimmed to only include the full-length putative off-target site. Putative off-target editing events were characterized as previously described^34^. In brief, the sequence encoded by the RTT was compared base-by-base to the nucleotide sequence 3′ of the cas9 nick site of each potential off-target site with an ‘off-target marker’ sequence being identified as the minimal deviating sequence between the two. The presence of this off-target marker sequence was quantified using output from CRISPResso2 and was used as a proxy for off-target prime editing. Oligonucleotides and results for this screen are recorded in Supplementary Table 11.

### rhAmpSeq off-target-site amplification and analysis

For initial off-target analysis, we used Cas-OFFinder to identify all human genomic sequences with up to 4 mismatches relative to our optimized epegRNA spacer sequence. A pooled sequencing primer was generated for nominated human off-target sites using the rhAmpSeq design tool (IDT). Genomic DNA was extracted from editor-treated HEK293T cells and amplified with rhAmpSeq pooled sequencing primers according to the manufacturer’s protocol. The amplified libraries were sequenced with 300-bp single-end reads with an Illumina MiSeq. Sequences for rhAmpSeq amplicons were extracted using the R Bioconductor BSGenome package (v.1.4.3) using the GRCh37/hg19 (human) reference genomes. CRISPResso2 was used to align the rhAmpSeq reads to the amplicon reference sequences and quantify the number of reads with each possible edit.

### Flow cytometry

Cells were trypsinized, resuspended in media containing 10% FBS, and the solution was filtered through a 45-μm cell strainer prior to flow cytometry or FACS isolation. Flow cytometry analysis was performed using the CytoFLEX LX Flow Cytometer (Beckman Coulter, C06779) at the Broad Institute Flow Cytometry Core, with CytExpert Acquisition and Analysis Software (v.2.4). FACS was performed on the SONY MA900 Cell Sorter (Sony Biotechnology) and cells were sorted into medium prior to being spun down.

### General cloning

Plasmid vectors for mammalian expression of epegRNAs or ngRNAs were cloned via isothermal assembly as previously described^67^. In brief, a human U6 promoter vector was linearized via polymerase chain reaction and incubated with IDT eBlocks encoding the full-length epegRNA or ngRNA sequence flanked by sequences necessary for isothermal assembly using NEBuilder HiFi DNA Assembly Master Mix (New England BioLabs). A list of epegRNA and ngRNA sequences used in this study are provided in Supplementary Table 1.

AAV vector genomes were cloned via isothermal assembly as previously described^50^. In brief, the v3em vector genome construct (Addgene, #198735) was linearized by restriction digest and new epegRNA and ngRNA sequences, encoded across two IDT eBlocks, were inserted via isothermal assembly using NEBuilder HiFi DNA Assembly Master Mix (New England BioLabs). The PE6d and PE6e v3em vectors were assembled via isothermal assembly using NEBuilder HiFi DNA Assembly Master Mix (New England BioLabs). The sequences of interest were PCR amplified out of existing vectors (Addgene, #207854 and 207855) and assembled into the corresponding v3em vector (Addgene, #198735 and #193734).

### Arrayed genome-editing experiments

For all experiments performed in HEK293T or HeLa readthrough reporter polyclonal cell lines and experiments performed in Neuro-2a cells, cells were seeded at 12,000 cells per well into 96-well plates (Corning) and transfected the following day with Lipofectamine 3000 (Thermo Fisher Scientific). A total of 200 ng, 66 ng and 22 ng of prime editor plasmid, epegRNA plasmid and ngRNA plasmid (where indicated), respectively, were transfected per well. 3 d after transfection, cells were lysed and genomic DNA was collected by incubation in a lysis buffer containing 10 mM Tris-HCl, pH 8.0, 0.05% SDS and 800 units per μl of proteinase K (New England BioLabs) at 37 °C for 1 h, followed by enzyme inactivation at 80 °C for 30 min.

For rescue experiments performed in the HEK293T disease models, cells were initially seeded at 200,000 cells per well in 12-well plates (Corning). The following day, cells were transfected with 700 ng of PE6c prime editor plasmid, 200 ng of epegRNA plasmid and 75 ng of ngRNA plasmid using Lipofectamine 3000 (Thermo Fisher Scientific). 3 d after transfection, genomic DNA was extracted as described above.

### Protein isolation and enzymatic activity assays

For assays using human cells, cells were collected by trypsinization, pelleted, washed in 1× phosphate-buffered saline and repelleted. For the tripeptidyl peptidase (TPP1) assay, pelleted cells were lysed at 4 °C for 30 min in a buffer containing 0.1% Triton X-100 (Sigma) and 10% SDS (Thermo Fisher Scientific) in 1× phosphate-buffered saline. For the hexosaminidase (HEXA) assay, pelleted cells were lysed in a RIPA homogenizing buffer supplemented with a protease inhibitor cocktail (Roche) at 4 °C for 30 min. In both cases, lysates were then centrifuged at 20,000*g* for 20 min at 4 °C. Supernatant was collected and total protein concentration was quantified using the Pierce BCA protein assay kit according to the manufacturer’s protocol (Thermo Fisher Scientific). For both HEXA and TPP1 assays, the sensitivity and accuracy of the assay was determined by a standard curve derived from measurements of known quantities of wild-type protein lysate (Supplementary Fig. 10b,c).

For the TPP1 assay, 10 μg of protein lysate was incubated at 37 °C overnight in a 0.1 M sodium acetate buffer at pH 4 containing the Ala-Ala-Phe-7-amido-4-methylcoumarin substrate (Sigma, A3401) at a final concentration of 250 μM in a reaction volume of 40 μl. Endpoint fluorescence was then measured with a Tecan Spark Multimode Microplate Reader with an excitation wavelength of 360 nm (20 nm bandwidth) and emission of wavelength of 460 nm (20 nm bandwidth). Enzymatic activity relative to wild-type was calculated by dividing the mean fluorescence values from each treatment condition by the mean fluorescence values of mock-treated wild-type HEK293T cells.

For the HEXA assay, 5 μg of protein lysate was incubated for 1 h at 37 °C in a 0.1 M citrate phosphate buffer at pH 4.5 containing either 4-methylumbelliferone-f-*N*-acetylglucosamine (MUG, Sigma, 69585) or 4-methylumbelliferone-*O*-*N*-acetylglucosamine-6-sulfate (MUGS, Sigma, 454428) substrate at a concentration of 3.2 mM. Each reaction was carried out in a total volume of 50 μl and stopped by the addition of 200 μl of a 100 mM solution of 2-amino-2-methyl-1-propanol. Endpoint fluorescence measurements were made with a Tecan Spark Multimode Microplate Reader with an excitation wavelength of 360 nm (20 nm bandwidth) and emission of wavelength of 450 nm (20 nm bandwidth). HEXA activity was normalized to HEXB activity by dividing the mean fluorescence values from the MUGS (HEXA) reaction by the corresponding mean fluorescence values from the MUG (HEXB) reaction. Enzymatic activity relative to wild-type was calculated by dividing the derived mean HEXA activity values for each treatment condition by that of the mean HEXA activity values from mock-treated wild-type HEK293T cells.

For measurements of α-L-iduronidase in mouse tissue, protein was extracted by first homogenizing the tissue using the TissueLyser II (QIAGEN) in T-PER tissue protein extraction reagent (Thermo Fisher Scientific, 78510) containing cOmplete Protease Inhibitor Cocktail (Roche). Lysates were then centrifuged at 20,000*g* for 20 min at 4 °C and supernatant was collected and total protein concentration was quantified using the Pierce BCA protein assay kit according to the manufacturer’s protocol (Thermo Fisher Scientific). Up to 40 μg of whole protein lysate was incubated overnight in a 130 mM sodium formate buffer containing 0.42 mg ml^−1^ of d-saccharic acid 1,4-lactone monohydrate (Sigma-Aldrich, S0375) and 4MU-iduronic acid (0.12 mM; Santa Cruz Biotechnology, sc-220961) at pH 3.5. Endpoint fluorescence measurements were made with a Tecan Spark Multimode Microplate Reader with an excitation wavelength of 365 nm (20 nm bandwidth) and emission of wavelength of 450 nm (20 nm bandwidth).

### Western blotting

Cells were collected by trypsinization, pelleted, washed in 1× phosphate-buffered saline then lysed in a RIPA homogenizing buffer supplemented with a protease inhibitor cocktail (Roche) at 4 °C for 30 min. Lysates were then centrifuged at 20,000*g* for 20 min at 4 °C. Supernatant was collected and total protein concentration was quantified using the Pierce BCA protein assay kit according to the manufacturer’s protocol (Thermo Fisher Scientific). Up to 10 μg of whole protein lysate was separated by SDS–PAGE using a 4–12% Bolt Bis-Tris Plus Mini Protein Gel (Invitrogen). Proteins were transferred to a nitrocellulose membrane using the iBlot 2 Dry Blotting system (Thermo Fisher Scientific) then incubated in a 5% milk solution in 1 × Tris-Buffered Saline, 0.1% Tween-20 (TBST). The blocked membrane was incubated at 4 °C overnight in NPC1 (Abcam, ab134113, 1:2,500 dilution) or GAPDH (Santa Cruz, sc-47724, 1:5,000 dilution) primary antibody. The following day, the membrane was incubated with horseradish peroxidase-conjugated goat anti-rabbit IgG (Abcam, ab6721; 1:10,000 dilution) for NPC1 and anti-mouse IgG (Abcam, ab205719; 1:10,000 dilution) for GAPDH for 1 h at room temperature. The secondary antibody was removed and the membrane was washed and signal was collected using the SuperSignal West Femto Maximum Sensitivity Substrate (Thermo Fisher Scientific) according to the manufacturer’s protocol.

### Protein isolation, trypsin digestion and TMT labelling for mass spectrometry

Protein was isolated as described for Western blotting. To prepare samples for mass spectrometry, samples were passed over S-trap micro spin columns (Profiti) according to manufacturer’s protocols with the following modifications: 10 mM DTT (final concentration) was used instead of TCEP. After adding DTT, tubes were placed on a heating block for 10 min at 95 °C. Disulfides were alkylated with 20 mM iodoacetamide (final concentration) instead of methyl methanethiosulfonate, and after adding the iodoacetamide the samples were incubated at 25 °C for 30 min in the dark. After digestion with 5 µg trypsin (Thermo Fisher 90057), samples were desalted using Pierce Peptide Desalting Spin Columns (Thermo Fisher 89852) following the manufacturer’s protocol. The desalted tryptic peptides were resuspended in 100 µl of 100 mM triethylammonium bicarbonate buffer (TEAB), vortexed, and briefly centrifuged. For labelling with tandem mass tags (TMTs), lyophilized TMTpro Label reagents (Thermo Fisher A44520) were used according to manufacturer’s protocols. At the time of labelling, aliquots were equilibrated at room temperature and 20 µl of anhydrous acetonitrile was added to each tube. The TMT reagents were vortexed, briefly centrifuged, and allowed to dissolve for 5 min at 25 °C. TMT reagents were added to each 100-µl sample, vortexed, and briefly centrifuged. The samples were incubated for 1 h at 25 °C. After 1 h, 5 µl of 5% hydroxylamine was added to each sample and incubated for 15 min to quench the reaction. Equal amounts of each labelled sample were then combined together and speed-vacuumed to dryness.

### LC–MS

The TMT-labelled tryptic peptides were separated by reverse phase HPLC (Thermo Ultimate 3000) using a Thermo PepMap RSLC C18 column (2 µm tip, 75 µm x 50 cm ES903) over a gradient before nano-electrospray using a Orbitrap Exploris 480 mass spectrometer (Thermo). Solvent A was 0.1% formic acid in water and solvent B was 0.1% formic acid in acetonitrile. The mass spectrometer was operated in a data-dependent mode. The parameters for the full scan MS were: resolution of 60,000 across 450–1,600 *m/z* and maximum IT 50 ms. The full mass spectrometry scan was followed by MS/MS for as many precursor ions in a three-second cycle with a NCE of 32, dynamic exclusion of 30 s and resolution of 45,000.

### Database search with Proteome Discoverer

Raw mass spectral data files (.raw) were searched using Sequest HT in Proteome Discoverer (Thermo). Sequest search parameters were: 10 ppm mass tolerance for precursor ions; 0.02 Da for fragment ion mass tolerance; 2 missed cleavages of trypsin. Fixed modifications were carbamidomethylation of cysteine and TMTpro modification on lysines and peptide N termini. Variable modifications were methionine oxidation, methionine loss at the N terminus of the protein, acetylation of the N terminus of the protein, and methionine loss plus acetylation of the protein N terminus.

### AAV production

AAV production was performed using HEK293T clone 17 cells (ATCC, CRL-11268) maintained in DMEM plus GlutaMAX (Thermo Fisher Scientific) with 10% heat-inactivated FBS without antibiotic in 150 mm^2^ dishes (Thermo Fisher Scientific). The day before transfection cells were plated at a density of 18 million cells per 150 mm^2^ plate. The following day, 5.7 μg of AAV genome plasmid, 11.4 μg of pHelper (Clontech) and 22.8 μg of rep-cap plasmid per plate were delivered via polyethyleneimine transfection (PEI MAX, Polysciences). Three days after transfection, cells were collected by cell scraping and pelleted at 3,000*g* for 10 min. Medium was decanted into a solution of poly(ethylene glycol) (PEG) 8000 (Sigma-Aldrich) and NaCl at a final concentration of 8% PEG and 500 mM NaC and incubated on ice for 2 h. The cell pellet was resuspended in 500 µl per plate of a hypertonic buffer containing 40 mM Tris base, 500 mM NaCl, 2 mM MgCl_2_ and 100 U ml^−1^ salt active nuclease (ArcticZymes) and incubated for one hour at 37 °C. The medium-containing solution was then pelleted by centrifugation at 3,000*g* for 30 min and the resulting pellet was resuspended in 500 µl per plate of a hypertonic lysis buffer and combined with the cell lysate. This lysate was then added to Beckman Coulter Quick-Seal tubes via 16-gauge, 5-inch needles (Air-Tite N165) in a discontinuous gradient of iodixanol in sequentially floating layers as follows: 9 ml of 15% iodixanol in 500 mM NaCl and 1× phosphate-buffered saline-MK (1× phosphate-buffered saline plus 1 mM MgCl_2_ and 2.5 mM KCl), 6 ml of 25% iodixanol in 1× phosphate-buffered saline-MK and 5 ml each of 40% and 60% iodixanol in 1× phosphate-buffered saline-MK with phenol red at a concentration of 1 μg ml^−1^ in the 15%, 25% and 60% layers to aid layer visualization This gradient was then ultracentrifuged using a fixed-angle Ti 70 rotor in an Optima XPN-100 Ultracentrifuge (Beckman Coulter) at 68,000 rpm for one hour at 18 °C. Next, 3 ml of virus-containing solution was extracted from the 40–60% iodixanol interface via an 18-gauge needle. Buffer was exchanged for cold phosphate-buffered saline with 0.001% F-68 using a PES 100 kD MWCO column (Thermo Fisher Scientific) and concentrated. The resulting AAV-containing solution was sterile filtered using a 0.22-μm filter and quantified by quantitative PCR (qPCR) (AAVpro Titration Kit version 2, Clontech). Purified virus was stored at 4 °C until use.

### Animal use

All experiments involving live animals were approved by the Broad Institute Institutional Animal Care and Use Committee (D16-00903; 0048-04-15-2). Mouse housing facilities were maintained at 20–22 °C with 30–50% humidity, on a 12 h light/12 h dark cycle with ad libitum access to standard rodent diet and water. For experiments involving *Idua*^*W392X*^ mice, we used Strain from Jackson Laboratory.

### Neonatal ventricular injections

Syringes for microinjection were prepared by pulling PCR Micropipettes (Drummond Scientific Company, 5-000-1001-X10) with the Sutter P1000 micropipette puller for a tip diameter size of 100 μm. The injection solution was prepared by mixing 5 × 10^10^ vg of each half of prime editor encoding AAV and 1 × 10^10^ vg of reporter AAV in 0.9% NaCl solution (Covetrus, 061758) in total volume of 4 μl, along with 0.1 μl of Fast Green. A total of 4 μl of injection solution was front-loaded to the micropipette syringe for injection. Neonatal mice were anaesthetized on ice. A total of 2 μl of injection solution was injected into each ventricle with successful injection being verified by the spread of Fast Green dye by transillumination of the head. Litters were randomized for injection with a given AAV composition. Both sexes were included for each experimental condition for in vivo experiments. Both sexes were assigned to each experimental group as evenly as possible. No statistics were performed to pre-determine sample size for each group.

### In vivo prime editing

In experiments demonstrating readthrough of an exogenous reporter co-administered via AAV, we evaluated both TAG and TGA stop codon readthrough. To achieve TGA stop codon readthrough, we used the epegRNA architecture optimized for the introduction of the CUA anticodon but modified the 3′ extension to introduce a UCA anticodon. The reporter construct contained an eGFP expression cassette with either a TAG or TGA PTC at codon 81 or the wild-type glutamine codon. In experiments evaluating the long-term tolerability of PERT, we delivered editing reagents in the v3em dual-AAV architecture introducing either an CUA anticodon into the mouse tRNA-Leu-TAA-2-1 gene, or a control +5 G-to-T edit in the *Dnmt1* locus. At three weeks, the cortices and livers from mice were processed for whole proteome mass spectrometry to evaluate readthrough past natural stop codons.

### Mouse tissue collection, histology and immunohistochemistry

Mice used in this study were sacrificed by CO_2_ asphyxiation, and unperfused tissues were immediately dissected. For protein and DNA analysis, dissected tissues were immediately frozen in liquid nitrogen. Protein extraction was performed as described above. Genomic DNA and RNA was extracted using the QIAGEN AllPrep DNA/RNA kit according to the manufacturer’s protocol.

For histology and immunohistochemistry, dissected tissues were placed in 10% neutral buffered formalin and then soaked in 70% ethanol prior to paraffin embedding. Following routine processing and paraffin embedding, tissue sections were stained with either haematoxylin and eosin, Alcian blue, or prepared for immunohistochemistry. For immunohistochemistry, 4-μm-thick sections were deparaffinized and rehydrated, followed by antigen retrieval using a sodium citrate buffer. After quenching endogenous peroxidase and application of a protein block (Dako), sections were incubated with either an anti-GFP antibody (Abcam, ab183734) or an anti-iduronidase (R&D systems, AF4119) antibody. Staining was detected using a species-specific detection kit, diaminobenzidine was used as the chromogen, and Mayer’s Haematoxylin (Dako) was used as the counterstain. Primary antibodies were substituted with an appropriate negative-control IgG for negative-control slides. For Alcian blue staining, 4-μm formalin-fixed, paraffin-embedded tissue sections were deparaffinized and rehydrated. Sections were stained with the Alcian Blue–1%, pH 2.5 kit (Newcomer, 9102A). Tissue histopathology was performed at the University of Minnesota Comparative Pathology Shared Resource and pathology scoring was performed by a board-certified veterinary pathologist blinded to the treatment conditions.

### Statistics and reproducibility

All screens were performed in independent biological duplicates. Sample sizes for all other experiments and analyses are defined in the corresponding figure legends. Where performed, select comparisons are indicated within the figure, and *P* values can be found in corresponding Supplementary Tables.

### Reporting summary

Further information on research design is available in the Nature Portfolio Reporting Summary linked to this article.

## Online content

Any methods, additional references, Nature Portfolio reporting summaries, source data, extended data, supplementary information, acknowledgements, peer review information; details of author contributions and competing interests; and statements of data and code availability are available at 10.1038/s41586-025-09732-2.

## Supplementary information

Supplementary Information This file contains Supplementary Discussion including additional references, Supplementary Note, Supplementary Tables and Supplementary Figs. 1–15.

Reporting Summary

Supplementary Table 1 Oligonucleotides, epegRNA sequences, ngRNA sequences, and plasmid sequences.

Supplementary Table 2 Design and results for PE2 epegRNA screens to convert endogenous tRNAs into sup-tRNAs with TAG, TGA, and TAA reporters.

Supplementary Table 3 Design and results for initial lentiviral sup-tRNA screen.

Supplementary Table 4 Design and results for sup-tRNA leader sequence screen.

Supplementary Table 5 Design and results for follow-up sup-tRNA leader and terminator screen.

Supplementary Table 6 Design and results for saturation mutagenesis screening of individual sup-tRNAs.

Supplementary Table 7 Design and results for saturation mutagenesis screening of the Leu-TAA family of sup-tRNAs.

Supplementary Table 8 Mutation combinations, tRNA sequences, and and statistics related to Figure 3d.

Supplementary Table 9 Design and results for epegRNA screening of Leu-TAA-1-1 into a sup-tRNA with an adjacent synthetic target site in HeLa cells.

Supplementary Table 10 Design and results for epegRNA screening of Leu-TAA-1-1 into a sup-tRNA with an adjacent synthetic target site in HEK293T cells.

Supplementary Table 11 Design and results for lentiviral off-target screening of the epegRNA.

Supplementary Table 12 Evaluation of first and second stop codons for each protein in the human proteome.

Supplementary Table 13 Whole proteome mass spectrometry on human cells treated with PERT.

Supplementary Table 14 Target-ion mass spectrometry on potential 3′UTR peptides for the top 69 proteins with a TAG stop codon in cells treated with PERT.

Supplementary Table 15 Design and results for sequence contexts amenable to readthrough with a CUA sup-tRNA.

Supplementary Table 16 CFTR cDNA constructs used in validation of sequence context screening results.

Supplementary Table 17 Whole proteome mass spectrometry on mouse livers and cortices treated with PERT.

## Source data

Source Data Fig. 5

## Data Availability

Amplicon next generation sequencing data have been deposited to the NCBI Sequence Read Archive database under accession PRJNA1335297. Source data are provided with this paper.

## Code availability

Bioinformatics analysis was performed using publicly available programs and parameters described in the Methods. The code for CRISPResso is available at https://github.com/pinellolab/CRISPResso2.
