# Supplementary material for: Prime editing-installed suppressor tRNAs for disease-agnostic genome editing
Source: Nature. 2025 Nov 19;648(8092):191–202. doi: 10.1038/s41586-025-09732-2 (PMC12675287; doi:10.1038/s41586-025-09732-2)
Supplement: Supplementary file 1 — This file contains Supplementary Discussion including additional references, Supplementary Note, Supplementary Tables and Supplementary Figs. 1–15. [file 41586_2025_9732_MOESM1_ESM.pdf]

nature portfolio

https://doi.org/10.1038/s41586-025-09732-2

Supplementary information

Prime editing-installed suppressor tRNAs for disease-agnostic genome editing

In the format provided by the authors and unedited

Supplementary Information

Prime editing-installed suppressor tRNAs for disease-agnostic genome editing

Sarah E. Pierce*, Steven Erwood*, Keyede Oye, Meirui An, Nicholas Krasnow, Emily Zhang, Aditya Raguram, Davis Seelig, Mark J. Osborn, David R. Liu

	Page #
**Supplementary Discussion**	2
**Supplementary Note**	10
**Supplementary Tables**	19
**Supplementary Figure 1**	20
**Supplementary Figure 2**	21
**Supplementary Figure 3**	22
**Supplementary Figure 4**	23
**Supplementary Figure 5**	24
**Supplementary Figure 6**	25
**Supplementary Figure 7**	26
**Supplementary Figure 8**	27
**Supplementary Figure 9**	28
**Supplementary Figure 10**	29
**Supplementary Figure 11**	30
**Supplementary Figure 12**	31
**Supplementary Figure 13**	32
**Supplementary Figure 14**	33
**Supplementary Figure 15**	34

Supplementary Discussion

Using exogenous promoters to express sup-tRNAs

hU6 and minU6 promoters are class III Pol III promoters, whereas endogenous tRNA promoters are class II. These promoter classes are distinguished by the involvement of a single protein component, and their transcription start sites also vary. We hypothesize that some tRNAs might be particularly sensitive to full-length transcription by a class II promoter, and processing of the extended 5' end by RNase P might be functionally important for the tRNA maturation process in general. This processing does not seem to be essential for the Leu-TAA family of tRNAs, which also have the advantage that they do not use their anticodon loop for recognition by the leucyl aminoacyl-tRNA synthetase[1].

Of note, *tRNA-Leu-TAA-4-1* was the top-performing sup-tRNA in the original exogenous promoter screen using a hU6 promoter, but this tRNA was not active when expressed with its endogenous leader sequence and its endogenous downstream sequence, which does not contain a polyT. In contrast, *tRNA-Leu-TAA-4-1* was enriched when expressed with its endogenous leader sequence and either a 4T or 5T terminator. We were unable to convert the endogenous *tRNA-Leu-TAA-4-1* gene into a functional sup-tRNA with prime editing (**Supplementary Fig. 8**) and *tRNA-Leu-TAA-4-1* is not highly expressed in HEK293T cells (**Supplementary Fig. 8**) or in any tissue we analyzed with corresponding tRNA-seq data. We hypothesize this lack of expression is due to its lack of an endogenous termination sequence. Taken together, these data support that sup-tRNAs are overall expressed best with their endogenous leader sequence but often benefit from a stronger synthetic terminator.

Saturation mutagenesis of Leu-TAA tRNAs

The original saturation mutagenesis screen was performed on the mature sequence of *tRNA-Leu-TAA-4-1*, but *tRNA-Leu-TAA-1-1* is a stronger sup-tRNA when expressed from its endogenous locus (**Supplementary Fig. 8d**). Since the mature sequence of each of the *Leu-TAA* tRNAs differs from that of *tRNA-Leu-TAA-4-1* by up to 11 nucleotides, we designed a follow-up screen in which we performed saturation mutagenesis using each of the *Leu-TAA* mature tRNA sequences as a starting point (*tRNA-Leu-TAA-1-1*, *tRNA-Leu-TAA-2-1*, *tRNA-Leu-TAA-3-1*, and *tRNA-Leu-TAA-4-1*) (**Supplementary Fig. 2, Supplementary Table 7**). We confirmed that the beneficial mutations identified in the *tRNA-Leu-TAA-4-1* screen were also helpful when mapped onto any of the four *Leu-TAA* tRNA sequences (**Extended Data Fig. 5b**). Perhaps unsurprisingly, several of the beneficial

mutations identified in one *Leu-TAA* family member are naturally present in the mature sequences of the other members.

Deletions in the variable loop were well-tolerated in *tRNA-Leu-TAA-1-1* and *tRNA-Leu-TAA-3-1* (**Supplementary Fig. 9a**). In this screen, we also included tRNA variants that switched out the entire variable loop of each sup-tRNA for the variable loop of all other Leu-tRNA isoacceptors, including from the *Leu-AAG*, *Leu-CAA*, and *Leu-TAG* isodecoder families. Most *Leu-TAA* sup-tRNAs tolerated these alternative variable loops and some switches improved the activity of the sup-tRNA; in particular, swapping out the *tRNA-Leu-TAA-1-1* variable loop for that of *tRNA-Leu-TAA-3-1* led to a >2-fold enrichment in sup-tRNA activity among GFP-positive cells (**Supplementary Fig. 9b).** Finally, we evaluated how naturally occurring mutations in *Leu-TAA* tRNAs in the human population performed in the saturation mutagenesis screen[2]. We observed that many of these naturally occurring mutations abrogate the resulting tRNA's function, further suggesting that some human tRNA genes have redundant functions and other tRNA isodecoders can compensate for their functional loss (**Extended Data Fig. 5c**).

Optimization of editing reagents to for the human Leu-TAA-1-1 tRNA gene

Editing tRNA genes presents unique challenges for epegRNA design. First, the high sequence similarity of tRNA gene families limits the number of available protospacer targets that uniquely target a given tRNA gene. Second, a key determinant of prime editing efficiency is how effectively the heteroduplex editing intermediate evades MMR, with contiguous or semi-contiguous tracts of mismatches tending to yield higher editing efficiencies than single-nucleotide mismatches[3]. When prime editing protein-coding regions, MMR evasion is typically achieved by testing combinations of translationally silent mutations alongside the desired edit. However, our saturation mutagenesis screening revealed that most mutations within a tRNA are not functionally silent. Given these limitations, we optimized editing conditions for all 19 identified mutation variants of interest simultaneously to identify edits that could be introduced with high efficiency while maintaining potent readthrough activity. We evaluated the editing capacity of PE6a, PE6b, PE6c, PE6d, PE6e, PE6f, PE6g, PEmax, and PEmaxΔRNaseH using a lentiviral library of epegRNAs paired with a synthetic target-site. This library allowed for the exhaustive testing of spacer variants, PBS lengths from 8 to 16 bp, RTT lengths from 21 to 36 bp, and combinations of each of the 19 mutation variants of interest. We performed the initial screens in HeLa cells (**Supplementary Fig. 2, Supplementary Table 9**). On average, a 21-nucleotide spacer with a non-template 5'-G yielded the highest mean editing efficiency (**Extended Data Fig. 7b**). Encouragingly, we found that each variant could be introduced with high average editing efficiency with the epegRNAs nominated by the target-matched screening data,

ranging from 18% for the anticodon-only variant and up to 42% for the hp13gc>cg+hp12ta>cg variant (**Fig. 3d,e**). The inclusion of MLH1dn increased the mean editing efficiencies for most mutation combinations evaluated, though the editing efficiency of mutation variants with combinations of three or four mutations (e.g. hp12ta>cg+hp13gc>cg+hp14gc>ta and hp12ta>cg+hp13gc>cg+hp14gc>ta+mut38a>t) did not increase, consistent with our expectation that these extensively recoded tRNA genes successfully evade MMR at the synthetic target site during prime editing because their heteroduplex intermediates do not resemble native MMR substrates, which contain small numbers of mismatches[3] (**Extended Data Fig. 7c**). To assess whether editing outcomes are dependent on cell-type, we transduced HEK293T cells with the epegRNA library and then transfected the transduced cell population with either PEmax or PE6c with or without MLH1dn (**Fig. 3c, Extended Data Fig. 7, Supplementary Table 10**). We observed that the relative editing efficiency of each tRNA variant at the synthetic target site and the impact of MMR inhibition were similar across the HeLa and HEK293T datasets (**Extended Data Fig. 7a,c**).

Readthrough efficiency across many pathogenic PTC contexts

The sequence context surrounding a PTC is a determinant of sup-tRNA readthrough efficiency. To evaluate the performance of PERT at a broad spectrum of nonsense mutations, we cloned 14,747 PTCs from the ClinVar database that were annotated as pathogenic, likely pathogenic, or of uncertain significance flanked on either side by the 18 nucleotides present in the native mRNA sequence into a nonsense-mediated decay (NMD)-sensitive reporter construct **(Supplementary Fig. 1, Supplementary Table 15)**. In addition, for each ClinVar library member, we included a 'no-premature-stop' control in which the TAG stop codon was replaced with a TTG leucine codon. We also included 2,800 'redundant stop' control library members, which were a subset of the ClinVar variants and their TGG controls but with the codon following the TAG stop codon or TGG codon changed to a TAA stop codon followed by a +1 frameshift to prevent readthrough. We evaluated the ability of a sup-tRNA to read through each PTC by evaluating stabilization of the mRNA transcript containing that nonsense mutation. We generated a lentiviral pool of this PTC sequence context library and transduced it into HEK293T cells that were prime edited to be homozygous for the ac-only sup-tRNA (*tRNA-Leu-TAA-1-1*) **(Supplementary Fig. 11a)**.

The vast majority of transduced cells were GFP-positive by FACS, indicating readthrough of a large fraction of the pathogenic PTCs. Since cells exhibiting GFP fluorescence in a pooled setting does not reveal the extent of readthrough in each individual cell, we devised an RNA-based measure of readthrough efficacy for each target TAG. We validated that the instability of the PTC-containing reporter transcript **(Supplementary Fig. 1a**) is the result of nonsense-mediated decay (NMD), and

that either a readthrough event with a sup-tRNA or the use of an NMD inhibitor stabilizes the transcript **(Supplementary Fig. 14a-b**). Consequently, if a given PTC-containing sequence was readthrough with higher frequency, we would expect transcripts encoding this sequence to be represented more frequently than those transcripts with sequences refractory to readthrough. We extracted both mRNA and genomic DNA from the transduced cell population, generated cDNA by reverse transcription, and sequenced the integrated reporter construct from both the cDNA and genomic DNA samples. By comparing the representation of each sequence at the RNA level compared to the DNA level, we calculated an 'RNA score' metric that reflects the ability of the ac-only sup-tRNA-Leu-TAA-1-1) to read through the PTC and stabilize that transcript. In addition, to control for the impact each unique sequence context might have on transcript expression level independent of readthrough activity, we defined a 'readthrough score' for each ClinVar PTC by dividing the RNA score of each variant by the RNA score of its corresponding sequence with a "TGG" leucine codon instead of a "TAG" stop codon **(Supplementary Table 15)**.

Similar to what we observed in the individual HEK293T disease models, readthrough scores varied both across and within individual genes, with a mean±SD readthrough score of 69%±30%. Consistent with our hypothesis that NMD would cause transcripts readthrough with higher efficiency to be more highly represented, controls with a TAA PTC that could not be readthrough with a TAG sup-tRNA had a lower mean RNA score than library members encoding a ClinVar PTC, whereas the TTG no-stop controls had a higher mean RNA score **(Supplementary Fig. 11b**). Representative readthrough scores using the cystic fibrosis transmembrane conductance regulator (CFTR) gene as an example are shown in Supplementary Fig. 11c. Recent reports suggest that the sequence determinants of readthrough extend beyond the short local sequence context surrounding a PTC80-83. To investigate this possibility, we sought to understand how readthrough scores correlated with the ability to readthrough a PTC in the context of a native full-length cDNA transcript using PTCs in the CFTR gene that cause cystic fibrosis. We cloned 15 pathogenic PTC CFTR variants into a lentiviral vector expressing full-length CFTR with a C-terminal GFP fusion. We transduced each of these variants into the HEK293T cell line homozygous for the ac-only sup-tRNA and into wild-type HEK293T cells. To calculate relative protein yield, we measured the mean GFP fluorescence intensity of each variant in each condition, subtracted the baseline signal observed in untreated HEK293T cells, and divided that value by the mean fluorescence intensity of a wild-type full-length CFTR control containing no PTC. Protein yields across the variants **(Supplementary Fig. 11c-d, Supplementary Table 16)** were moderately correlated (R = 0.49) with the readthrough scores measured in the pooled ClinVar screen. Therefore, while pooled PTC screens can help identify mutations that might be

especially poised for rescue with PERT, additional factors contribute to the ability of a sup-tRNA to readthrough a specific PTC.

Additional sequence variants in the mouse Leu-TAA-2-1 gene negligibly enhance readthrough

We performed saturation mutagenesis of three human tRNA genes *(tRNA-Arg-CCT-4-1*, *tRNA-Tyr-GTA-2-1* and *tRNA-Leu-TAA-1-1*) and the mouse tRNA gene *tRNA-Leu-TAA-2-1* in the context of TAG nonsense suppression (**Extended Data Fig. 4h**). While these screens yielded sequence variants for each human tRNA gene that were substantially enriched in suppression function relative to wild-type, comparable improvements were not observed for mouse *tRNA-Leu-TAA-2-1*, which appeared less amenable to functional optimization under our screening conditions (**Extended Data Fig. 4h**). This contrast is particularly noteworthy, as the mouse *tRNA-Leu-TAA-2-1* is orthologous to the human *tRNA-Leu-TAA-1-1* gene, which could be improved by up to 5-fold in suppression activity through sequence engineering under the same conditions (**Fig. 2d**). Across diverse gene and sequence contexts (**Figs. 2d, 4d,e, and Supplementary Fig. 10d**), engineered human *tRNA-Leu-TAA-1-1* variants demonstrated superior suppression capacity compared with anticodon-only edits, suggesting this finding was unlikely to be specific to the reporter sequence used in the screening efforts. Further experiments are required to clarify whether this discrepancy is specific to mouse *tRNA-Leu-TAA-2-1* or represents a more general constraint on engineering mouse tRNA genes. This uncertainty complicates pre-clinical evaluation of PERT, as it raises questions about the predictive value of mouse models relative to human systems. In practice, we used anticodon-only editing, or the introduction of more complex engineered edits, interchangeably in our in vivo work. Evaluation of therapeutic efficacy of PERT may require more emphasis on human model systems, such as primary tissue or organoids which can more accurately reflect the potency of suppression.

Optimization of editing reagents to for the orthologous mouse tRNA gene

The mouse ortholog of human *Leu-TAA-1-1* (mouse *Leu-TAA-2-1*) differs by one base pair in the sequence encoding the variable loop. This difference is part of both the spacer and PBS of our optimized epegRNA for the human sequence, necessitating re-optimization of our prime editing agents. We optimized prime editing agents to install either an ac-only sup-tRNA or the mouse equivalent of an engineered sup-tRNA (mouse tRNA-Leu-TAA-2-1+hp13gc>ta+mut38a>t). These two non-anticodon mutations were both enriched in the mouse *Leu-TAA-2-1* saturation mutagenesis screen (**Extended Data Fig. 4h**) and, when combined, performed best when expressed via lentiviral integration in the context of human *Leu-TAA-1-1* (**Fig. 2d**). We optimized epegRNA sequences through testing a panel of PBS and RTT combinations in mouse Neuro-2a cells. Given the size

constraints of AAV packaging that we anticipated using in our *in vivo* studies, we performed these experiments using the PEmax enzyme with a truncated RNAseH domain. We observed a maximum average editing efficiency of 16% for the anticodon-only edit when using an epegRNA with a PBS length of 12 and an RTT length of 19 (**Supplementary Fig. 15a**). The engineered sup-tRNA (mouse tRNA-Leu-TAA-2-1+hp13gc>ta+mut38a>t) could be encoded with a maximum average editing efficiency of 5.7% using an epegRNA with a PBS length of 15 and an RTT length of 30 (**Supplementary Fig. 15b**). These epegRNAs resulted in undesired target-site byproduct modifications (incomplete editing or indels) at 0.55% and 3% for the ac-only sup-tRNA and engineered sup-tRNA, respectively (**Supplementary Fig. 15a,b**).

Next, we investigated whether prime editing efficiencies could be further improved through the use of a PE3 or PE3b strategy. We evaluated a panel of 12 ngRNAs for each edit and found a +5 PE3b strategy led to the highest editing efficiency for both edits-of-interest. This PE3b strategy yielded an average desired editing efficiency of 20% with 1.3% average undesired editing for the ac-only sup-tRNA and an average desired efficiency of 14% with 6.7% average undesired editing for the engineered sup-tRNA (**Supplementary Fig. 15c,d**). Finally, we identified the most effective prime editor enzyme to use with the optimized PE3b strategy by comparing the editing efficiencies using each of the PE6 prime editor protein variants[4]. The PE6e prime editor variant was the most effective for the ac-only sup-tRNA, achieving a mean desired editing frequency of 32% with an undesired editing frequency of 5.9% (**Supplementary Fig. 15e**). For the engineered sup-tRNA edit, the PE6d prime editor variant led to the highest mean desired editing frequency of 30% with 13% undesired editing (**Supplementary Fig. 15f**).

MMR evasion might especially hinder prime editing at tRNA loci

We used both an MMR-proficient cell line and an MMR-deficient cell line in the process of optimizing our prime editing strategy. These experiments were performed by evaluating epegRNA editing efficiency on a synthetic target-site encoded on the lentiviral cassette and subsequently validated on the endogenous locus. We observed similar mean editing efficiencies of the synthetic target-site in both cell lines. In MMR-deficient HEK293T cells, we further observed a strong correlation between the editing efficiency achieved by a given epegRNA on the synthetic target and the endogenous locus. In MMR-proficient HeLa cells, however, the correlation was less pronounced and editing at the endogenous locus was substantially less effective than what was observed at the synthetic target-site. Of note, the only epegRNAs yielding mean editing efficiencies >10% were those encoding the most MMR-evasive edits (**Extended Data Figure 7a**). We speculate that tRNA genes, owing to their high expression and corresponding susceptibility to transcription-associated

mutagenesis (TAM)[5], could be more heavily surveilled by DNA repair machinery, such as MMR, and thus be more refractory to prime editing. Future optimizations of PERT should explore this potential difference between editing Pol III-driven genes compared to the most common use case of editing Pol II-driven genes.

Using sup-tRNAs to study endogenous tRNAs in general

Studying individual tRNAs is inherently challenging due to their high redundancy, with modifications to a single tRNA often having minimal phenotypic effects. However, creating endogenous sup-tRNAs offers a unique opportunity to examine tRNA sequences in isolation. By altering their anticodon loop, we achieved a readthrough readout directly tied to the expression of a single tRNA sequence, enabling focused analysis of tRNA-specific features such as the leader sequence and termination element. While a poor leader sequence can eliminate sup-tRNA activity, we could not identify consistent sequence features explaining why certain leader sequences perform worse than others. Furthermore, we do not yet know which positions within the 40-bp upstream of a tRNA are critical for defining the leader sequence. In contrast, the importance of a strong termination signal was universally observed across sup-tRNAs. These findings are likely just as relevant for our understanding of wild-type tRNA expression as they are for optimizing sup-tRNA function.

In-frame stop codon usage in the human genome

Notably, the frequent occurrence of redundant in-frame stop codons following NTCs greatly limits the extension of proteins beyond a suppressed stop codon. Across all human proteins, the median distance between the first in-frame stop codon and the second in-frame stop codon is 19 amino acids (**Supplementary Table 12**). For the 22% (4036/17945) of human proteins for which the first natural stop codon is TAG, 78% (3135/4036) of the second in-frame natural stop codons are TAA or TGA (**Supplementary Table 12**). Therefore, even if a TAG-specific sup-tRNA reads through an NTC, the effect would likely be limited by the presence of a second in-frame stop codon.

Supplementary Discussion References

1. Giege, R. & Eriani, G. The tRNA identity landscape for aminoacylation and beyond. *Nucleic Acids Res* **51**, 1528-1570 (2023).
2. Chan, P.P. & Lowe, T.M. GtRNAdb: a database of transfer RNA genes detected in genomic sequence. *Nucleic Acids Res* **37**, D93-97 (2009).
3. Chen, P.J. et al. Enhanced prime editing systems by manipulating cellular determinants of editing outcomes. *Cell* **184**, 5635-5652 e5629 (2021).
4. Doman, J.L. et al. Phage-assisted evolution and protein engineering yield compact, efficient prime editors. *Cell* **186**, 3983-4002 e3926 (2023).
5. Thornlow, B.P. et al. Transfer RNA genes experience exceptionally elevated mutation rates. *Proc Natl Acad Sci U S A* **115**, 8996-9001 (2018).

Supplementary Note

University of Minnesota Masonic Cancer Center
Comparative Pathology Shared Resource

CPSR Study Number:

24-125

Study Title
Histopathologic analysis of tissue from a mouse model of MPS I treated with AAV vector injection

Author:
Davis M. Seelig, DVM, PhD
Diplomate, A.C.V.P.

Comparative Pathology Shared Resource
Masonic Cancer Center
University of Minnesota
1352 Boyd Ave, St. Paul, MN 55108

Study Sponsor:
N/A

Table of Contents	
1. Pathologist's Signature	3
2. Study background and experimental objectives.	4
3. Gross necropsy findings (if applicable)	N/A
4. Clinical Pathology data (if applicable)	N/A
5. Methods (tissue trimming, sample sectioning, staining and/or immunohistochemistry and analytical approach).	4-5
6. Results – Descriptive gross and microscopic observations, representative images, and quantitative / semi-quantitative severity findings.	5-8

1. Regulatory Compliance Statement and Data Integrity

This non-GLP study was conducted for research and development purposes. Sample receipt, processing, analysis and data reporting are performed in alignment with the best practices of The Society of Toxicologic Pathology. Histopathological evaluation was done in the spirit of FDA regulations on good laboratory practices (GLP) for nonclinical laboratory studies CFR title 21 part 58. The chain of custody for tissues analyzed and slides reported was verified by the study pathologist. The undersigned signifies that they approve all pathology-related data within this report, including tables, narrative, and overall summary.

Pathologist's Signature

Study Title: Histopathologic analysis of tissue from a mouse model of MPS I treated with AAV vector injection

CPSR Study Number: 24-125

Sponsor Study Number: N/A

Study Pathologist(s)

Signature: ____________________________ Date: 11NOV24
Davis M. Seelig, DVM, PhD
Diplomate, A.C.V.P.

2. **Study background and experimental objectives (from submitter)**
    a. The goal of this project is to restore the lysosomal enzyme IDUA in a murine model of Mucopolysaccharidoses type IH (https://www.ncbi.nlm.nih.gov/pmc/articles/PMC2795040/). A gene editing approach was undertaken using intracerebroventricular AAV vector injection into newborn mice. The animals were sacrificed at 6.8 weeks post injection and are submitted for H&E assessment and glycosaminoglycan analysis by microscopy. We will seek to analyze the image meta data with the University Imaging Core for comparative analysis across animals. Please notify us before tissue disposal so we can confirm.

3. **Methods**
    a. Received tissues were paraffin-embedded, sectioned, mounted onto slides and stained with Hematoxylin and Eosin (H&E) and Alcian Blue (AB) using standard techniques. At the direction of the submitter, select tissues were subjected to anti-GFP and/or anti-IDUA immunohistochemistry. See Table 1 for a summary of the submitted samples and the performed staining.

Table 1. Submitted samples and tissue-specific staining

<table>
<tr><th>CPSR ID</th><th>Investigator Animal ID & Genotype</th><th>List of tissues</th><th>H&E</th><th>AB</th><th>GFP IHC</th><th>IDUA IHC</th></tr>
<tr><td>24-125-1</td><td>Mouse 1 Idua -/- +AAV</td><td>brain, liver, spleen, kidney, heart</td><td rowspan="10">All tissues</td><td rowspan="10">All tissues</td><td>Brain</td><td>N/A</td></tr>
<tr><td>24-125-2</td><td>Mouse 2 Idua -/- +AAV</td><td>brain, liver, spleen, kidney, heart</td><td>Brain</td><td>N/A</td></tr>
<tr><td>24-125-3</td><td>Mouse 3 WT</td><td>brain, liver, spleen, kidney, heart</td><td>All tissues</td><td>Brain</td></tr>
<tr><td>24-125-4</td><td>Mouse 4 WT</td><td>brain, liver, spleen, kidney, heart</td><td>Brain</td><td>N/A</td></tr>
<tr><td>24-125-5</td><td>Mouse 5 WT</td><td>brain, liver, spleen, kidney, heart</td><td>Brain</td><td>N/A</td></tr>
<tr><td>24-125-6</td><td>Mouse 6 WT</td><td>brain, liver, spleen, kidney, heart</td><td>Brain</td><td>N/A</td></tr>
<tr><td>24-125-7</td><td>Mouse 7 Idua -/- +AAV</td><td>brain, liver, spleen, kidney, heart</td><td>All tissues</td><td>Brain</td></tr>
<tr><td>24-125-8</td><td>Mouse 8 het no AAV</td><td>brain, liver, spleen, kidney, heart</td><td>Brain</td><td>N/A</td></tr>
<tr><td>24-125-9</td><td>Mouse 9 Idua -/- no AAV</td><td>brain, liver, spleen, kidney, heart</td><td>All tissues</td><td>Brain</td></tr>
<tr><td>24-125-10</td><td>Mouse 10 Idua -/- no AAV</td><td>brain, liver, spleen, kidney, heart</td><td>Brain</td><td>N/A</td></tr>
<tr><td>24-125-11</td><td>Fetal liver injection</td><td>brain, liver, spleen, kidney, heart</td><td>Brain</td><td>Brain</td><td>All except brain</td><td>N/A</td></tr>
<tr><td>24-125-12</td><td>Fetal liver injection</td><td>brain, liver, spleen, kidney, heart</td><td>Brain</td><td>Brain</td><td>All except brain</td><td>N/A</td></tr>
</table>

b. H&E slides were evaluated for MPS-related pathology – 1) hepatic foam cell accumulation, 2) cerebellar Purkinje cell vacuolization, and 3) splenic stromal vacuolization. Alcian Blue stained slides were evaluated for the intracellular accumulation of AB-positive glycosaminoglycan. Both anti-GFP and IDUA immunostained samples were evaluated for the distribution and intensity of staining.
c. All samples were evaluated semi-quantitatively as outlined in Table 2.

Table 2. Semi-quantitative scoring scheme for histology samples

Score	Descriptor
0	No staining or MPS-related microscopic pathology seen
1	Minimal staining or MPS-related microscopic pathology seen
2	Mild staining or MPS-related microscopic pathology seen
3	Moderate staining or MPS-related microscopic pathology seen
4	Marked staining or MPS-related microscopic pathology seen

4. Results (the study pathologist was unblinded after analysis and scoring):

a. H&E-stained sections:
   i. MPS associated H&E microscopic pathology was only identified in the tissues from the Idua -/- mice (Mice # 1, 2, 7, 9-11).
      1. Within this group, moderate cerebellar Purkinje cells vacuolization was found in the three mice not treated with intracerebroventricular AAV Vector (# 9-11). In contrast, in the three Idua -/- mice treated with intracerebroventricular AAV vector (#1, 2, and 7), this vacuolization had decreased to absent, absent, and minimal, respectively.
   ii. A similar pattern of decreasing vacuolization with AAV vector treatment was identified in the spleen, liver, and heart.
      1. In the spleen, moderate vacuolization was seen in Mice 9 and 10, but this vacuolization was of minimal and mild severity in mice 1-2, 7, and 11. In the liver, the two Idua -/- mice not treated with AAV vector (# 9 and 10), there was minimal and mild foam cell accumulation, respectively. In contrast, in the four AAV vector treated mice, the foam cell accumulation was minimal in two (# 7 and 11) and absent in two (# 1 and 2). In the hearts of the two untreated Idua -/- mice (# 9 and 10), minimal to mild vessel-associated foam cells were identified. In contrast, in the three Idua -/- mice treated with intracerebroventricular AAV vector (# 1, 2, and 7), no such cells were found. In the one mouse treated with intrahepatic AAV vector (# 11), a mild accumulation of vessel-associated foam cells was seen.

b. AB-stained sections:
   i. No AB staining was detected in the brains of any of the study animals, including mice in which cerebellar Purkinje cell vacuolization was previously identified. This lack of staining likely reflects the limited

sensitivity of AB staining and should not be considered as definitive evidence of a lack of GAG accumulation.

ii. Alcian Blue staining was detected only in the livers from Idua -/- mice (Mice #s 1, 7, 9, and 10). In the 2 mice untreated with intracerebroventricular AAV vector (Mice #s 9 and 10), this staining was of mild intensity, but was decreased to absent to minimal in the treated mice (Mice #s 1, 2, and 7) .

iii. Widespread, minimal AB staining was detected in the spleen and kidney sections. In light of the diffuse morphology of the staining and its identical severity across all mice, this staining is interpreted as background and not considered significant.

c. Anti-GFP IHC stained sections:

i. Marked and widespread anti-GFP immunostaining was detected only in the brains of the 3 Idua -/- mice treated with intracerebroventricular AAV vector (Mice #s 1, 2, and 7). In the peripheral tissues of these mice, anti-GFP immunostaining was detected only in the liver and heart of Mouse #7. In the two mice treated with intra-hepatic AAV vector (Mice #s 11 and 12), no anti-GFP immunostaining was detected in any tissues. See Table 5 for detailed anti-GFP IHC results.

d. Anti-IDUA IHC stained sections

i. No anti-IDUA immunostaining was detected in the brains from the 3 stained animals (Mice #s 3, 7, and 9). See Table 6 for detailed anti-IDUA IHC results.

Table 3. Semiquantitative scoring of MPS associated microscopic findings – H&E stain

CPSR ID	Investigator Animal ID & Genotype	Brain*	Spleen**	Liver***	Kidney	Heart***
24-125-1	Mouse 1 Idua -/- +AAV	0	1	0	0	0
24-125-2	Mouse 2 Idua -/- +AAV	0	1	0	0	0
24-125-3	Mouse 3 WT	No Cbm	0	0	0	0
24-125-4	Mouse 4 WT	No Cbm	0	0	0	0
24-125-5	Mouse 5 WT	No Cbm	0	0	0	0
24-125-6	Mouse 6 WT	0	0	0	0	0
24-125-7	Mouse 7 Idua -/- +AAV	1	2	1	0	0
24-125-8	Mouse 8 het no AAV	No Cbm	0	0	0	0
24-125-9	Mouse 9 Idua -/- no AAV	3	3	2	0	2
24-125-10	Mouse 10 Idua -/- no AAV	3	3	1	0	1
24-125-11	Fetal liver injection	3	2	1	0	2
24-125-12	Fetal liver injection	0	0	0	0	0

Cbm = Cerebellum

* Brain scoring reflects the magnitude of vacuolization in cerebellar Purkinje cells.

** Spleen scoring reflects the magnitude of rarefaction of the capsular and stromal mesenchymal cells.

*** Liver and heart scoring reflects the magnitude of foam cell accumulation in portal areas and vascular / perivascular spaces, respectively.

Table 4. Semiquantitative scoring of MPS-associated microscopic findings – AB stain

CPSR ID	Investigator Animal ID & Genotype	Brain*	Spleen	Liver**	Kidney	Heart**
24-125-1	Mouse 1 Idua -/- +AAV	0	1	1	1	1
24-125-2	Mouse 2 Idua -/- +AAV	0	1	0	1	1
24-125-3	Mouse 3 WT	No Cbm	1	0	1	1
24-125-4	Mouse 4 WT	No Cbm	1	0	1	1
24-125-5	Mouse 5 WT	No Cbm	1	0	1	1
24-125-6	Mouse 6 WT	0	1	0	1	1
24-125-7	Mouse 7 Idua -/- +AAV	0	1	1	1	1
24-125-8	Mouse 8 het no AAV	No Cbm	1	0	1	1
24-125-9	Mouse 9 Idua -/- no AAV	0	1	2	1	2
24-125-10	Mouse 10 Idua -/- no AAV	0	1	2	1	1
24-125-11	Fetal liver injection	0	1	N/A	N/A	N/A
24-125-12	Fetal liver injection	0	1	N/A	N/A	N/A

Cbm = Cerebellum

* Brain scoring reflects the magnitude of AB-positive GAG in cerebellar Purkinje cells.

** Liver and heart scoring reflects the magnitude of foam cell accumulation in portal areas and vascular / perivascular spaces, respectively.

Table 5. Semiquantitative scoring of anti-GFP immunostaining intensity

CPSR ID	Investigator Animal ID & Genotype	Brain*	Spleen	Liver**	Kidney	Heart**
24-125-1	Mouse 1 Idua -/- +AAV	4	N/A	N/A	N/A	N/A
24-125-2	Mouse 2 Idua -/- +AAV	4	N/A	N/A	N/A	N/A
24-125-3	Mouse 3 WT	0	0	0	0	0
24-125-4	Mouse 4 WT	0	N/A	N/A	N/A	N/A
24-125-5	Mouse 5 WT	0	N/A	N/A	N/A	N/A
24-125-6	Mouse 6 WT	0	N/A	N/A	N/A	N/A
24-125-7	Mouse 7 Idua -/- +AAV	4	0	1	0	2
24-125-8	Mouse 8 het no AAV	0	N/A	N/A	N/A	N/A
24-125-9	Mouse 9 Idua -/- no AAV	0	0	0	0	0
24-125-10	Mouse 10 Idua -/- no AAV	0	N/A	N/A	N/A	N/A
24-125-11	Fetal liver injection	N/A	0	0	0	0
24-125-12	Fetal liver injection	N/A	0	0	0	0

Table 6. Semiquantitative scoring of brain anti-IDUA immunostaining intensity

CPSR ID	Investigator Animal ID & Genotype	Brain*
24-125-3	Mouse 3 WT	0
24-125-7	Mouse 7 Idua -/- +AAV	0
24-125-9	Mouse 9 Idua -/- no AAV	0

5. Representative Images

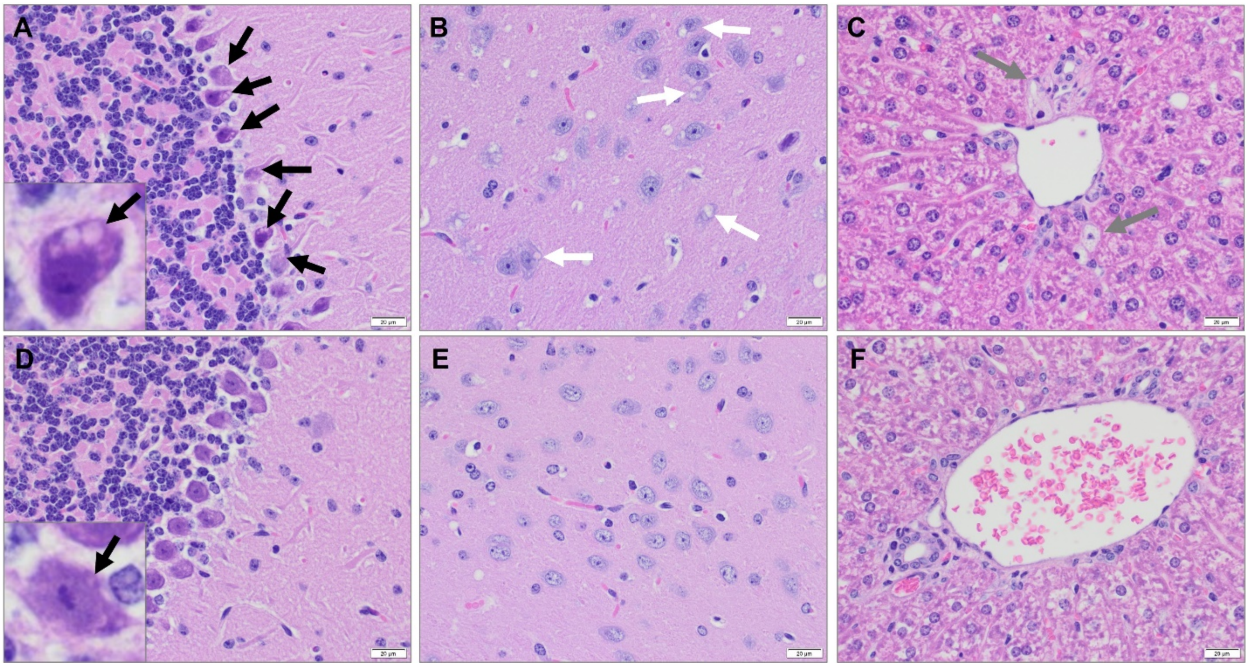

Figure 1. Brain and liver, representative images, Idua -/-, +/- ICV AAV vector, H&E. In panels A-C (untreated mouse #9), note the moderate intracellular vacuolization of cerebellar Purkinje cells (black arrows, A) and thalamic neurons (white arrows, B) and the hepatic foam cells (gray arrows, C). In contrast, in panels D-F (treated mouse #7), there is minimal cerebellar Purkinje cell vacuolization (black arrow, inset, D), a lack of thalamic neuronal vacuolization (E), and a lack of hepatic foam cells (F).

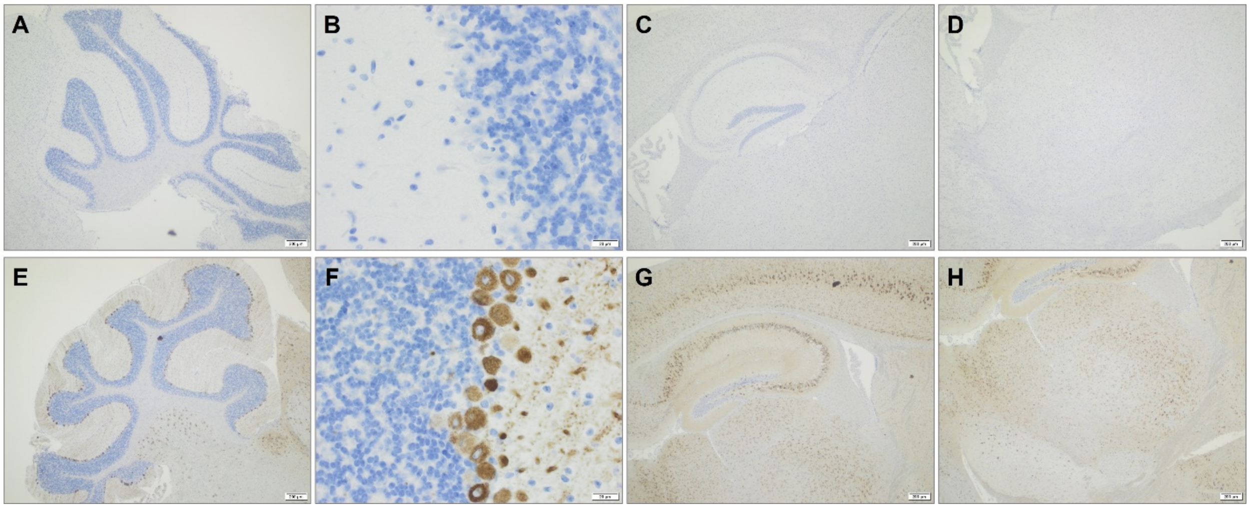

Figure 2. Brain, representative images, Idua -/-, +/- ICV AAV vector, GFP-IHC. In panels A-D (untreated mouse #9), note the complete lack of anti-GFP immunostaining, including within the cerebellum (A), Purkinje cells (B), hippocampus (C), and thalamus (D). In contrast, in the intracerebroventricular AAV vector treated mouse (panels E-H, mouse #7), there is widespread anti-GFP immunostaining in the same sites.

Supplementary Tables (provided as separate Excel files)

Supplementary Table 1. Oligonucleotides, epegRNA sequences, ngRNA sequences, and plasmid sequences.

Supplementary Table 2. Design and results for PE2 epegRNA screens to convert endogenous tRNAs into sup-tRNAs with TAG, TGA, and TAA reporters.

Supplementary Table 3. Design and results for initial lentiviral sup-tRNA screen.

Supplementary Table 4. Design and results for sup-tRNA leader sequence screen.

Supplementary Table 5. Design and results for follow-up sup-tRNA leader and terminator screen.

Supplementary Table 6. Design and results for saturation mutagenesis screening of individual sup-tRNAs.

Supplementary Table 7. Design and results for saturation mutagenesis screening of the Leu-TAA family of sup-tRNAs.

Supplementary Table 8. Mutation combinations, tRNA sequences, and and statistics related to Figure 3d.

Supplementary Table 9. Design and results for epegRNA screening of Leu-TAA-1-1 into a sup-tRNA with an adjacent synthetic target site in HeLa cells.

Supplementary Table 10. Design and results for epegRNA screening of Leu-TAA-1-1 into a sup-tRNA with an adjacent synthetic target site in HEK293T cells.

Supplementary Table 11. Design and results for lentiviral off-target screening of the epegRNA.

Supplementary Table 12. Evaluation of first and second stop codons for each protein in the human proteome.

Supplementary Table 13. Whole proteome mass spectrometry on human cells treated with PERT.

Supplementary Table 14. Target-ion mass spectrometry on potential 3'UTR peptides for the top 69 proteins with a TAG stop codon in cells treated with PERT.

Supplementary Table 15. Design and results for sequence contexts amenable to readthrough with a CUA sup-tRNA.

Supplementary Table 16. CFTR cDNA constructs used in validation of sequence context screening results.

Supplementary Table 17. Whole proteome mass spectrometry on mouse livers and cortices treated with PERT.

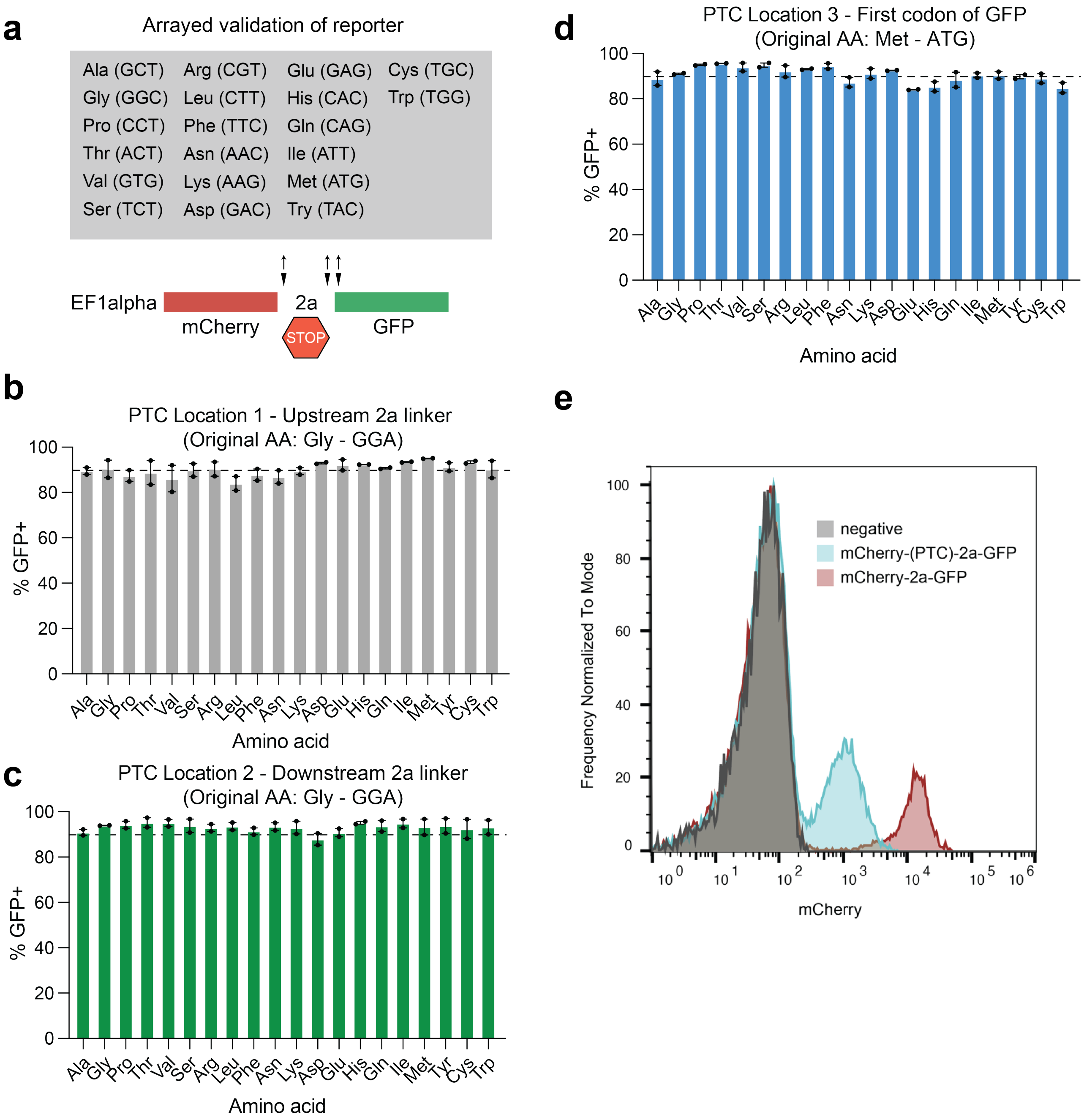

Supplementary Figure 1. Validating reporter constructs for readthrough of premature stop codons. (**a**) Schematic of reporter system with an EF1-alpha promoter driving expression of an mCherry fluorescent protein, followed by a 2a ribosomal skipping element, followed by a GFP fluorescent protein. Premature termination codons were installed at one of three positions, indicated by black arrows. Amino acids at these positions were switched to all 20 possible amino acids. (**b-d**) % GFP+ cells following transfection of plasmids with indicated positions switched to any of the 20 indicated amino acids, for PTC location 1 (b), PTC location 2 (c), or PTC location 3 (d). (**e**) Flow cytometry data representing the 10-fold loss in mCherry protein expression observed when a TAG PTC is included in the lentiviral reporter system at Location 1 (pSEP0201) compared to WT GFP with a GGA codon at the same position (pSEP0211). Frequency of events normalized to the mode indicated on the y-axis. Values and error bars reflect mean±s.d. of n=2 independent biological replicates.

Related Main Figure	Supp Table #	Screen Name	Cell Line	Lentiviral Backbone	Perturbation	Number of Unique Elements	Selection Method	Screening Output
Fig. 1	#2	PE2 - TAG	HEK293T; TAG reporter	pSEP0308	PE: epegRNAs XXX>CTA	18,000	FACS	epegRNA sequence
		PE2 - TGA	HEK293T; TGA reporter		PE: epegRNAs XXX>TCA	18,000		
		PE2 - TAA	HEK293T; TAA reporter		PE: epegRNAs XXX>TTA	18,000		
Extended Data Fig. 4	#3	Lentiviral exogenous promoter screen - TAG	HEK293T; TAG reporter	pSEP0308	sup-tRNA (hU6)	1,011	FACS	sup-tRNA sequence
				pSEP0309	sup-tRNA (min-U6)	1,011		
				pSEP0310	sup-tRNA (no exogenous promoter)	1,011		
	#4	Leader sequence	HEK293T; TAG reporter	pSEP0310	sup-tRNA (40-bp leaders)	838	FACS	leader sequence
	#5	Secondary leader/ terminator screen	HEK293T; TAG reporter	pSEP0310	sup-tRNA (40-bp leaders + terminators)	11,544	FACS	leader + terminator sequence
	#6	Saturation mutagenesis *Leu-TAA-4-1*	HEK293T; TAG reporter	pSEP0308	sup-tRNA (hU6)	1,067	FACS	sup-tRNA sequence
				pSEP0309	sup-tRNA (min-U6)	1,067		
				pSEP0310	sup-tRNA (no exogenous promoter)	1,067		
	#6	Saturation mutagenesis *Tyr-GTA-2-1*	HEK293T; TAG reporter	pSEP0310	sup-tRNA (endogenous leader)	457	FACS	sup-tRNA sequence
	#6	Saturation mutagenesis *Arg-CCT-4-1*	HEK293T; TAG reporter	pSEP0310	sup-tRNA (endogenous leader)	526	FACS	sup-tRNA sequence
	#6	Saturation mutagenesis mouse *Leu-TAA-4-1*	HEK293T; TAG reporter	pSEP0310	sup-tRNA (endogenous leader)	528	FACS	sup-tRNA sequence
Fig. 2	#7	Leu-TAA family SatMut	HEK293T; TAG reporter	pSEP0308	sup-tRNA (hU6)	2,174	FACS	sup-tRNA sequence
Fig. 3	#9	Self-targeting PE	HeLa; TAG reporter	pSEP0308	PE: epegRNAs with mutation combinations	18,000	Editing efficiency (synthetic target)	epegRNA sequence + target site
	#8		HEK293T; TAG reporter					
Supplemental Fig. 11	#10	Sequence context	HEK293T; *Leu-TAA-1-1* TAA>CTA	pSEP0211	Readthrough sequence context	32,294	RNA stability	RNA counts vs. DNA counts

Supplementary Figure 2. Overview of pooled lentiviral screens performed in this study.

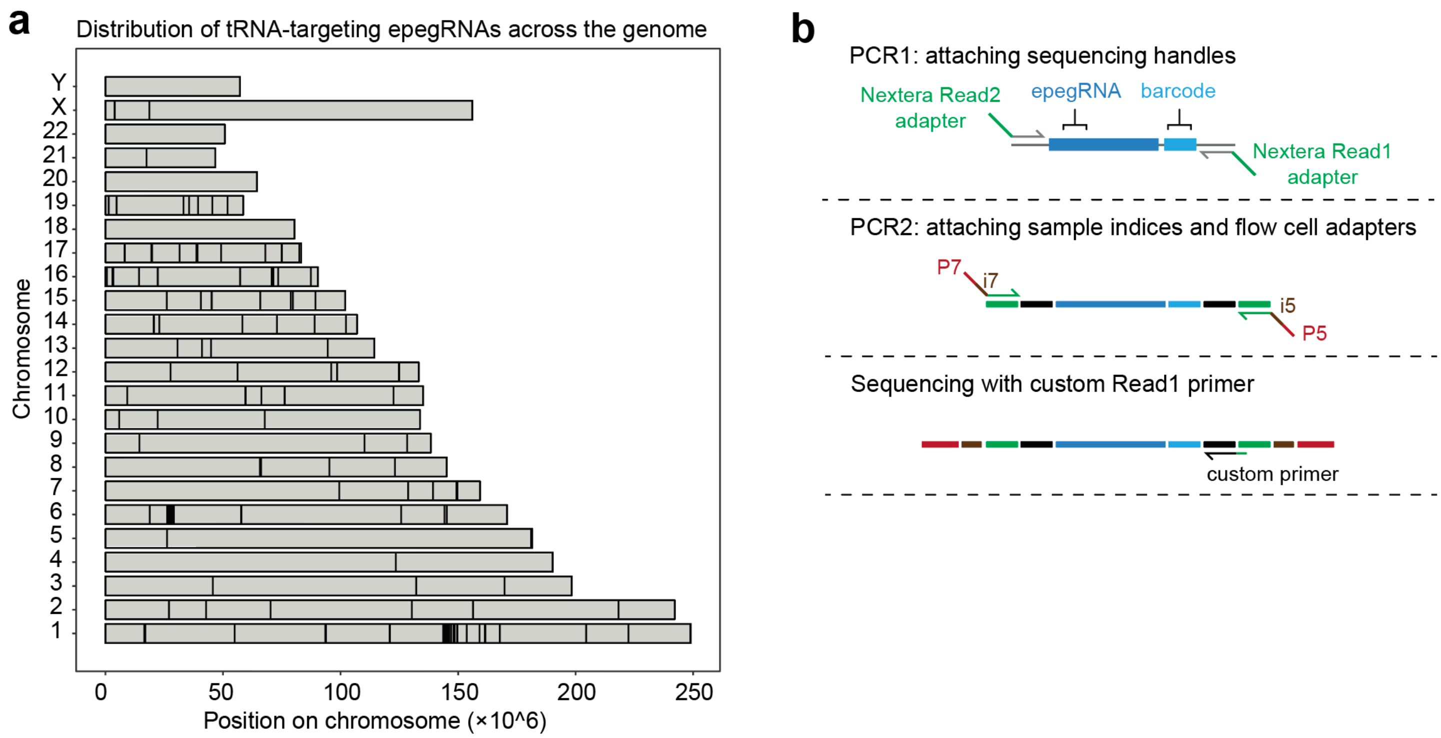

Supplementary Figure 3. epegRNA design and NGS library generation for PE2 screens. (**a**) Distribution of tRNA-targeting epegRNAs across the genome, with spacers targeted indicated by black lines across each chromosome. (**b**) PCR amplification strategy for PE2 screens. PCR1 involves attaching sequencing handles with primers that have the Nextera Read1 and Nextera Read2 sequences attached. PCR2 involves attaching sample indices and flow cell adapters for Illumina-based sequencing. Sequencing is performed with a custom primer that reads directly into a diverse barcode representation of each epegRNA.

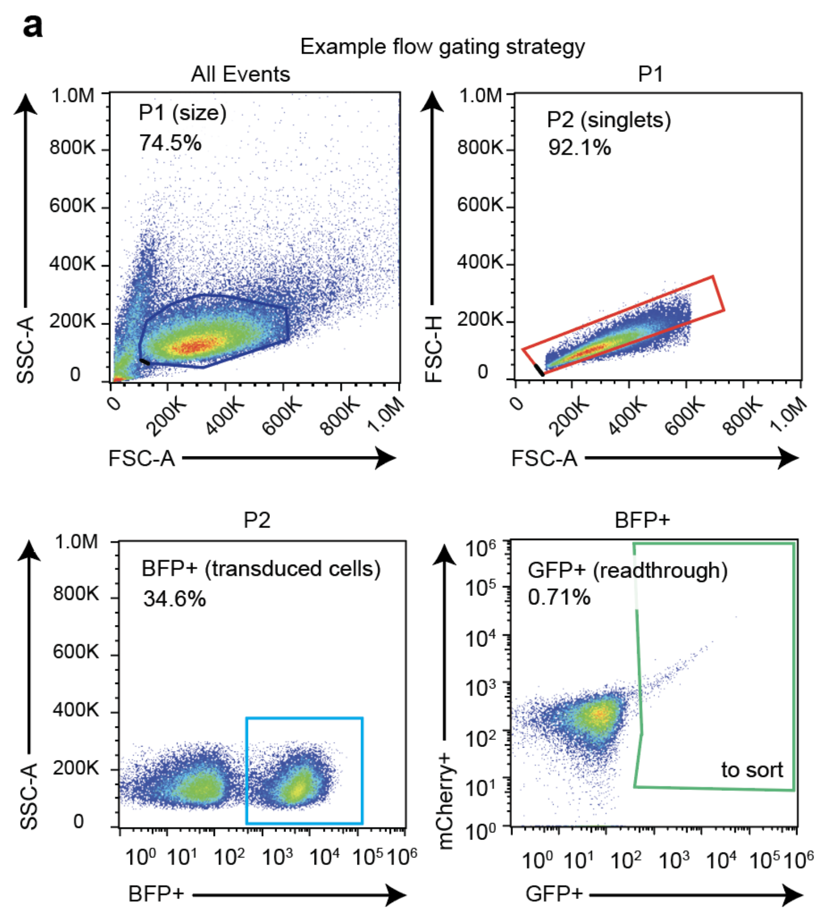

b

Example recording

	Name	Events	% Parent	% Total
■	All Events	100,000	0.00%	100.00%
■	P1	74,500	74.50%	74.50%
■	P2	68,615	92.10%	68.61%
■	BFP+	23,741	34.60%	23.74%
■	GFP+	169	0.71%	0.17%

Supplementary Figure 4. FACS gating strategy for sorting-based screens. (**a**) Example flow gating strategy for the PE2 screens. Cells are gated by size, singlets, transduced cells, and GFP+ readthrough. (**b**) Example recording of flow cytometry data for the indicated gates from (a).

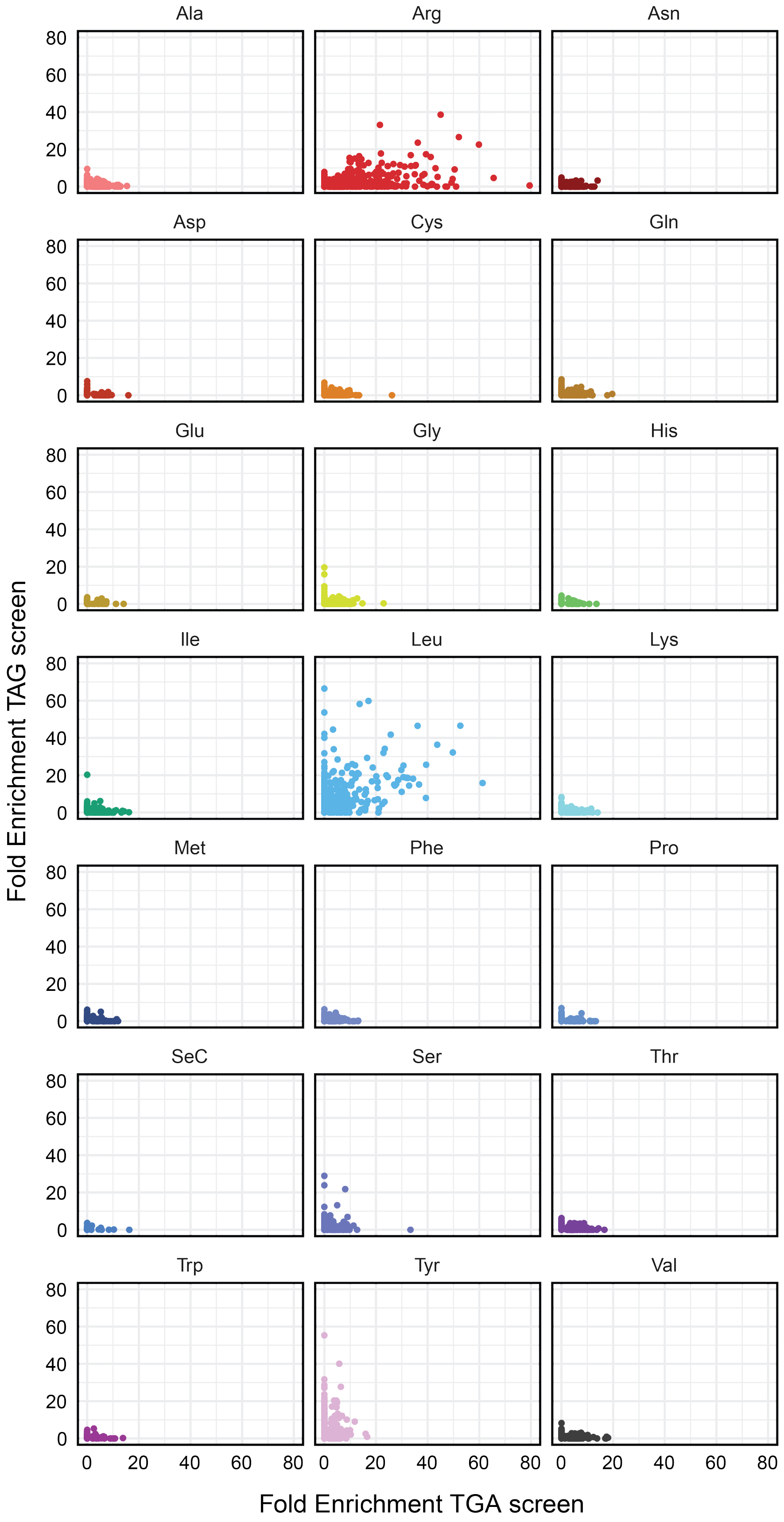

Supplementary Figure 5. Sup-tRNA backbones for reading through TAG and TGA stop codons are differentially enriched. Fold enrichment of epegRNAs in the GFP-sorted population in the TAG screen (y-axis) compared to the TGA screen (x-axis).

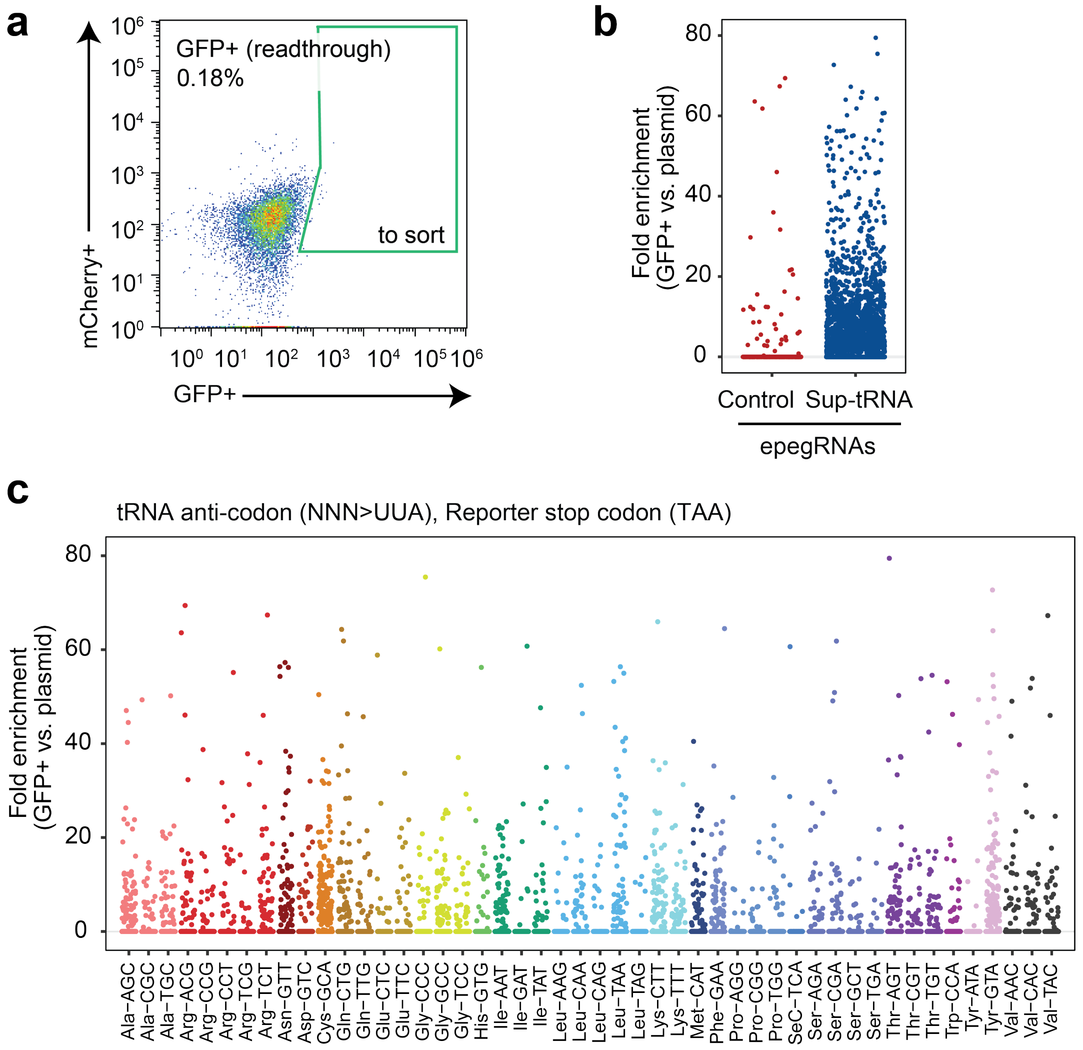

Supplementary Figure 6. Endogenous tRNA genes could not be converted to efficient TAA sup-tRNAs with prime editing. (**a**) Example flow cytometry data for TAA screen for GFP+ cells. Only 0.18% cells had readthrough and expression of GFP in these cells was low. (**b,c**) Fold enrichment in GFP+ cells compared to the plasmid pool for both control epegRNAs and sup-tRNA generating epegRNAs (b), as well as broken down by amino acid family (c). Compared to the TAG and TGA screens, the signal-to-noise ratio is much smaller.

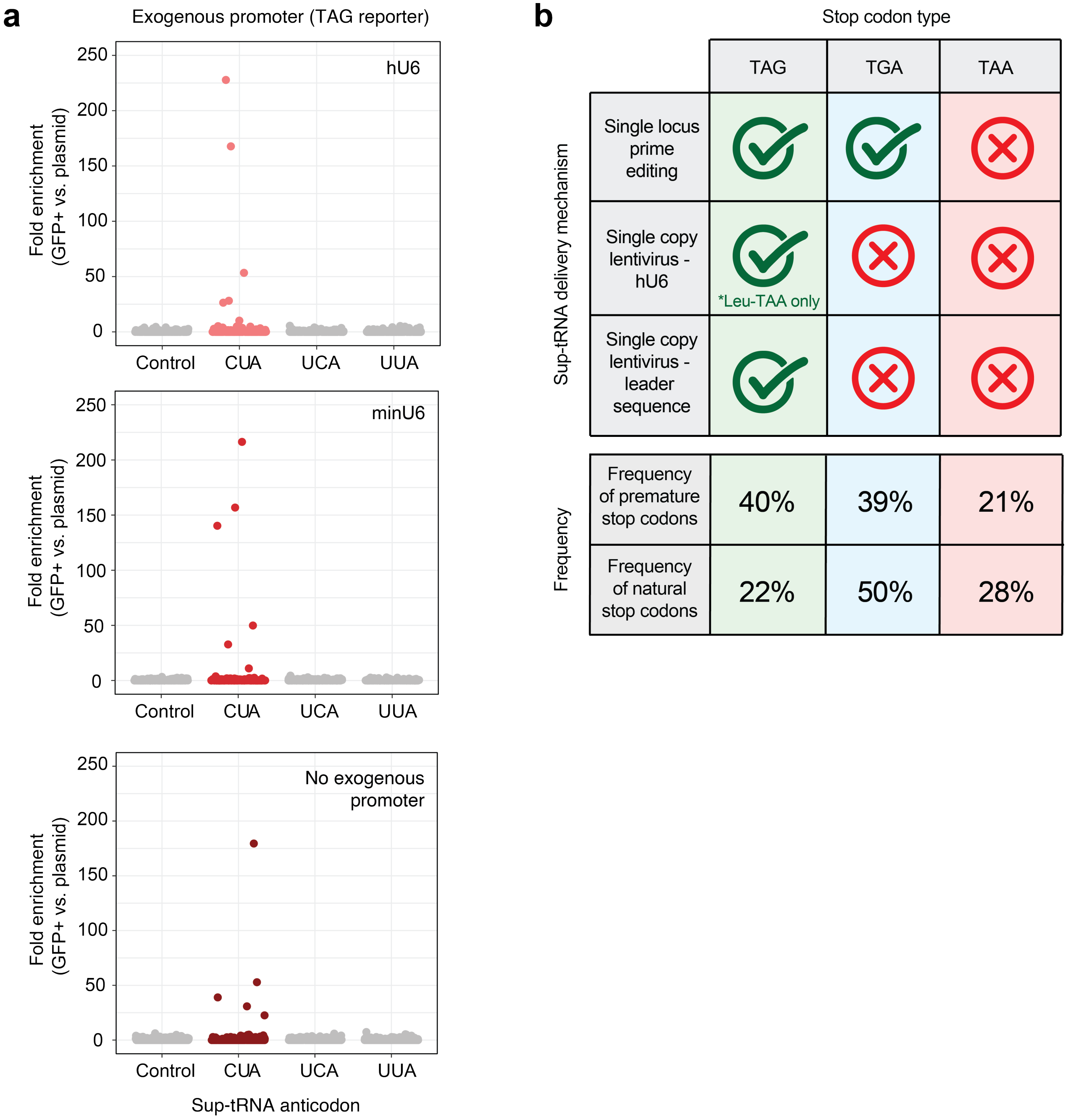

Supplementary Figure 7. Engineering the upstream sequences of sup-tRNAs. (**a**) Fold enrichment among GFP-positive cells versus the plasmid pool of sup-tRNA sequences expressed using a hU6 promoter (*top*), minU6 promoter (*middle*), or with no exogenous promoter (*bottom*). The anticodon of the sup-tRNA is indicated. (**b**) Summary of how the delivery mechanism of a sup-tRNA coupled with its surrounding sequence influences its effectiveness to read through a GFP stop codon reporter. Check marks indicate identification of an ac-only sup-tRNA capable of reading through the indicated stop codon.

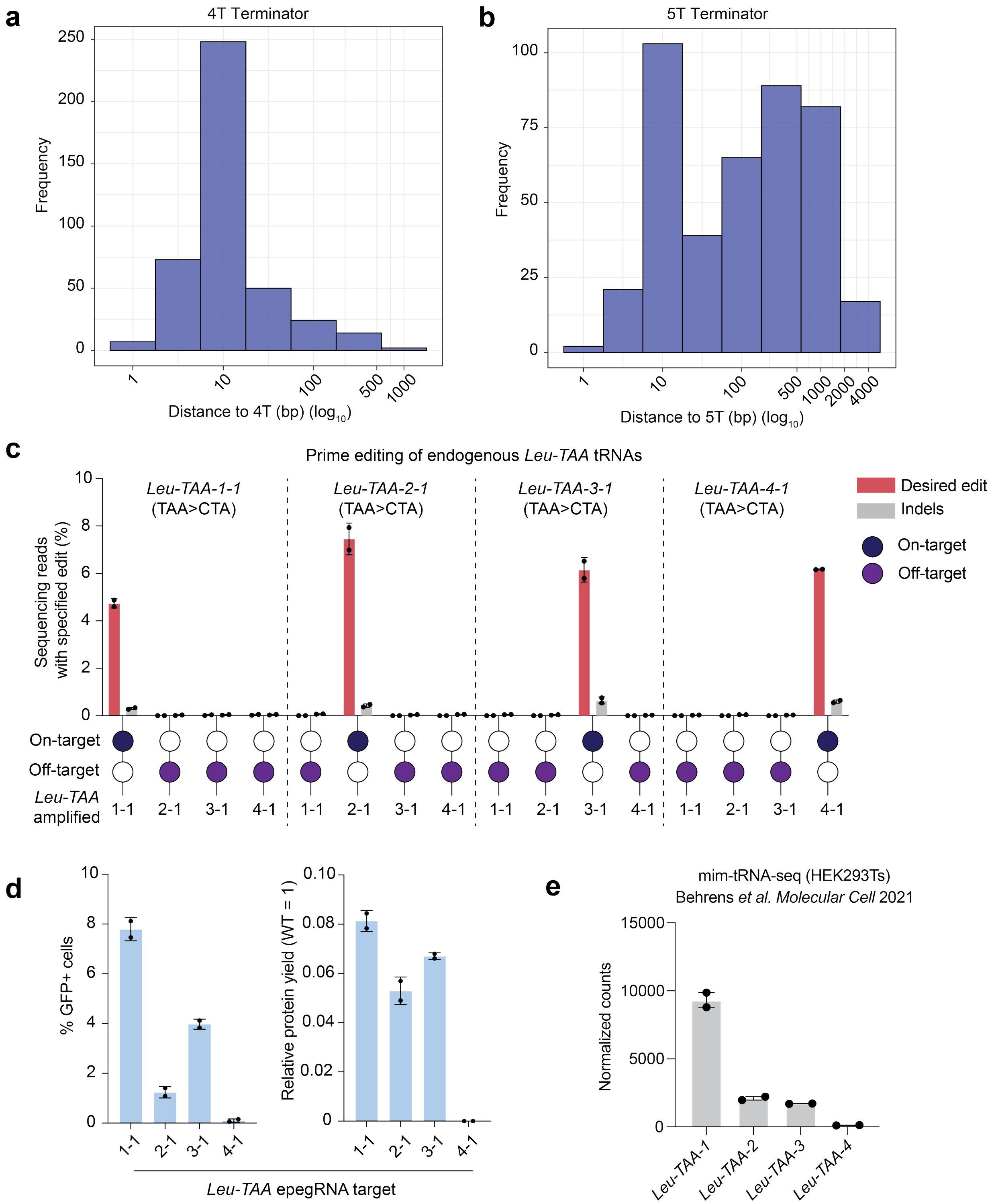

Supplementary Figure 8. Engineering the downstream sequences of sup-tRNAs. (**a**,**b**) Log_{10} distance in bp to a naturally occurring 4T (a) or 5T (b) sequence in the genome for each endogenous human tRNA gene. (**c**) Sequencing reads with the specified edit (%) following transfection with prime editing reagents designed to target each of the endogenous *Leu-TAA* tRNAs independently. (**d**) Percent GFP-positive cells (*left*) and relative protein yield relative to wild-type GFP (*right*) with a GFP stop codon readthrough reporter following prime editing of each of the four *Leu-TAA* tRNAs into sup-tRNAs. (**e**) Normalized counts of each *Leu-TAA* tRNA from a HEK293T mim-tRNA-seq dataset. Values and error bars reflect mean±s.d. of n=2 independent biological replicates.

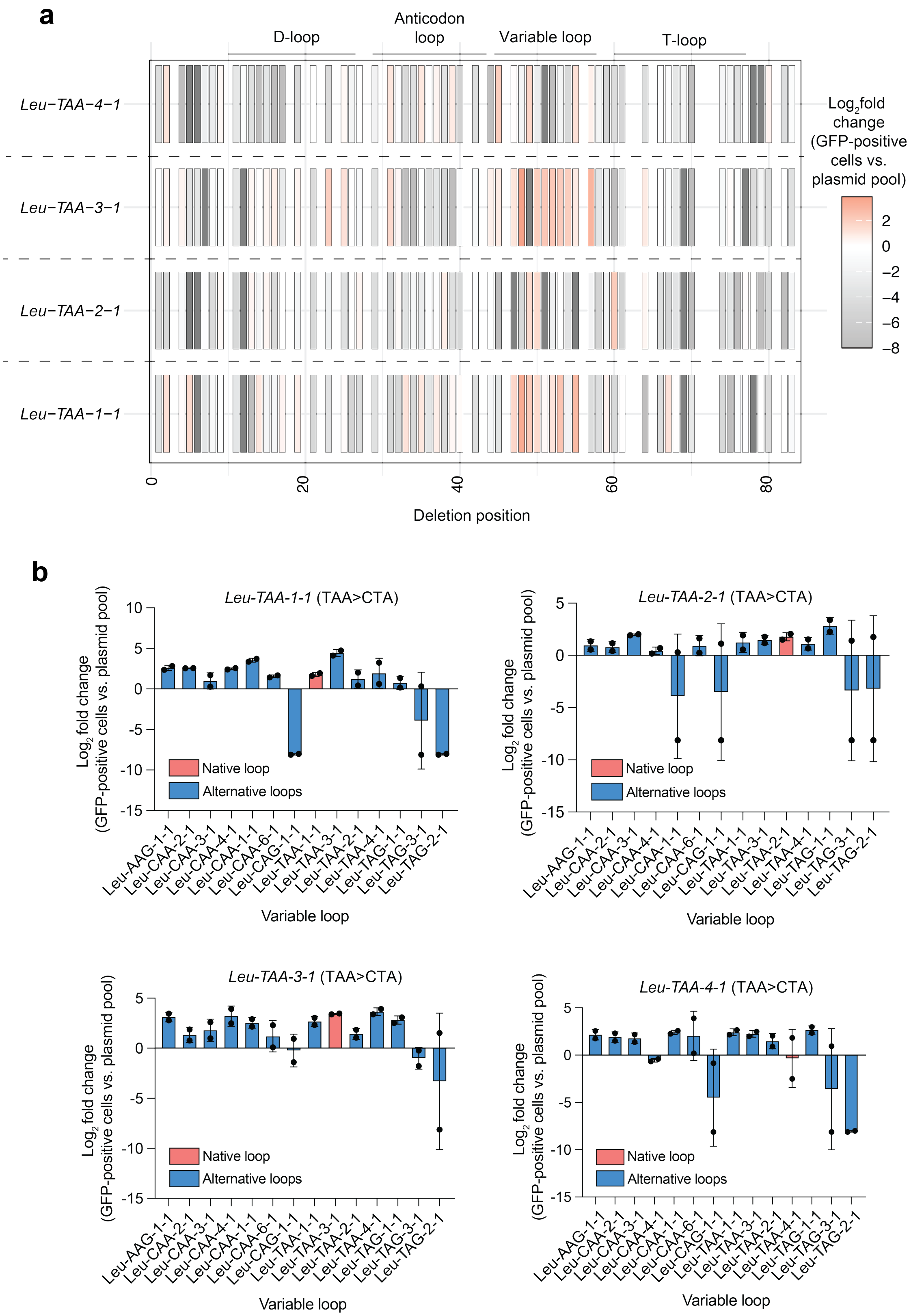

Supplementary Figure 9. Effect of deletions and variable loop replacement in Leu-TAA sup-tRNAs. (**a**) Heat map of the $\log_2$ fold change in abundance of each indicated single-nt deletion mutant among the GFP-positive cells compared to the plasmid pool for each of the four *Leu-TAA* sup-tRNAs. (**b**) Log_2 fold change in abundance of the indicated sup-tRNA among the GFP-positive cells compared to the plasmid pool when swapping each tRNA's native variable loop with the variable loop of the indicated leucine tRNAs. N=2 independent biological screening replicates.

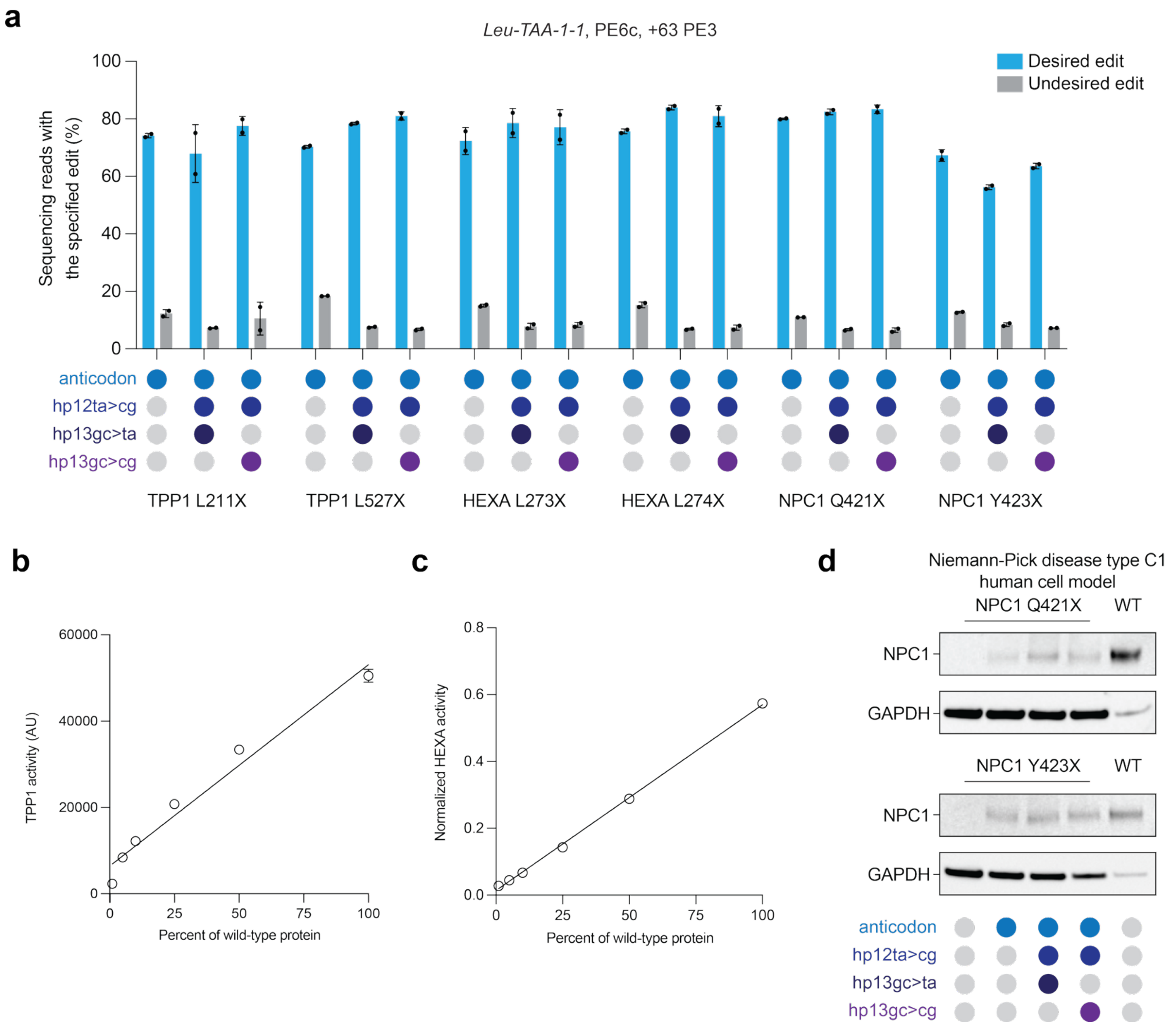

Supplementary Figure 10. Editing outcomes and validation of functional assays in human cells models of disease. (**a**) Measured editing outcomes in six HEK293T cell models of disease for each specified edit. (**b**) Standard curve showing accuracy of measured TPP1 activity across various concentrations of wild-type protein (AU = arbitrary units). (**c**) Standard curve showing accuracy of measured HEXA activity (normalized to HEXB activity) across various concentrations of wild-type protein. Values and error bars reflect mean±s.d. of n=2 independent biological replicates. (**d**) Measurement of NPC1 protein expression via Western blot in treated versus untreated human cell models of Niemann-Pick disease type C1 relative to wild-type controls. wild-type protein lysate was loaded at 1/10th the quantity of all other samples. Representative image that is reflective of n=2 independent biological replicates.

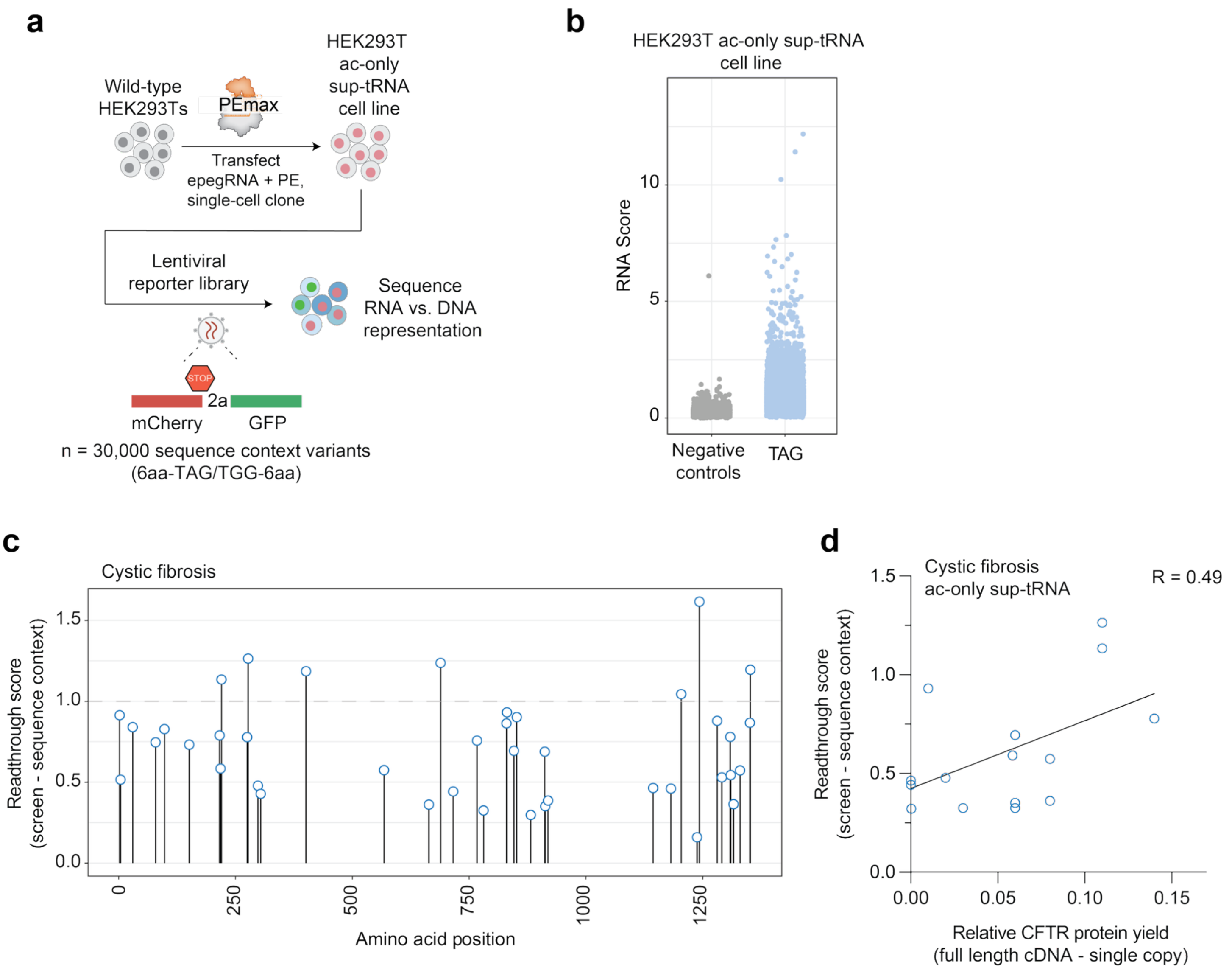

Supplementary Figure 11. PERT rescues expression of PTCs across diverse sequence contexts. (**a**) Schematic of screening strategy to identify the impact of sequence context on readthrough efficacy. (**b**) RNA score of each sequence context library subgroup. (**c**) Readthrough scores for each variant in the *CFTR* gene measured in the sequence context library screen. (**d**) Sequence context RNA score plotted against relative CFTR protein yield from arrayed expression of full-length *CFTR* mutants. Values and error bars reflect mean±s.d. of n=2 independent biological replicates.

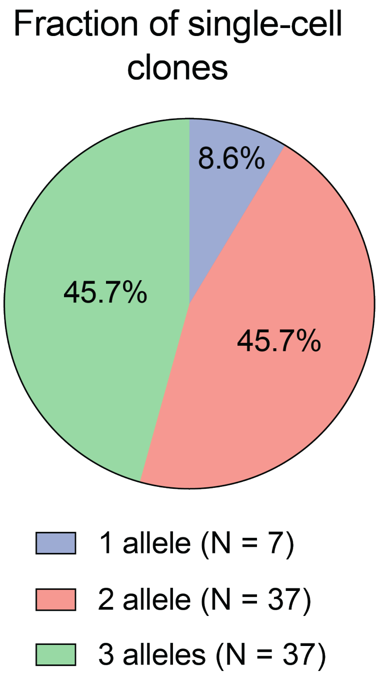

Supplementary Figure 12. Number of sup-tRNA alleles installed in HEK293T cells among edited cells. Following plasmid transfection of prime editing agents to convert the endogenous genomic tRNA-Leu-TAA-1-1 gene into the ac-only sup-tRNA in HEK293T cells, individual colonies were grown and sequenced at the edited locus, which is triploid in HEK293T cells. Among edited cells, the fraction with one, two or all three chromosomes edited is shown.

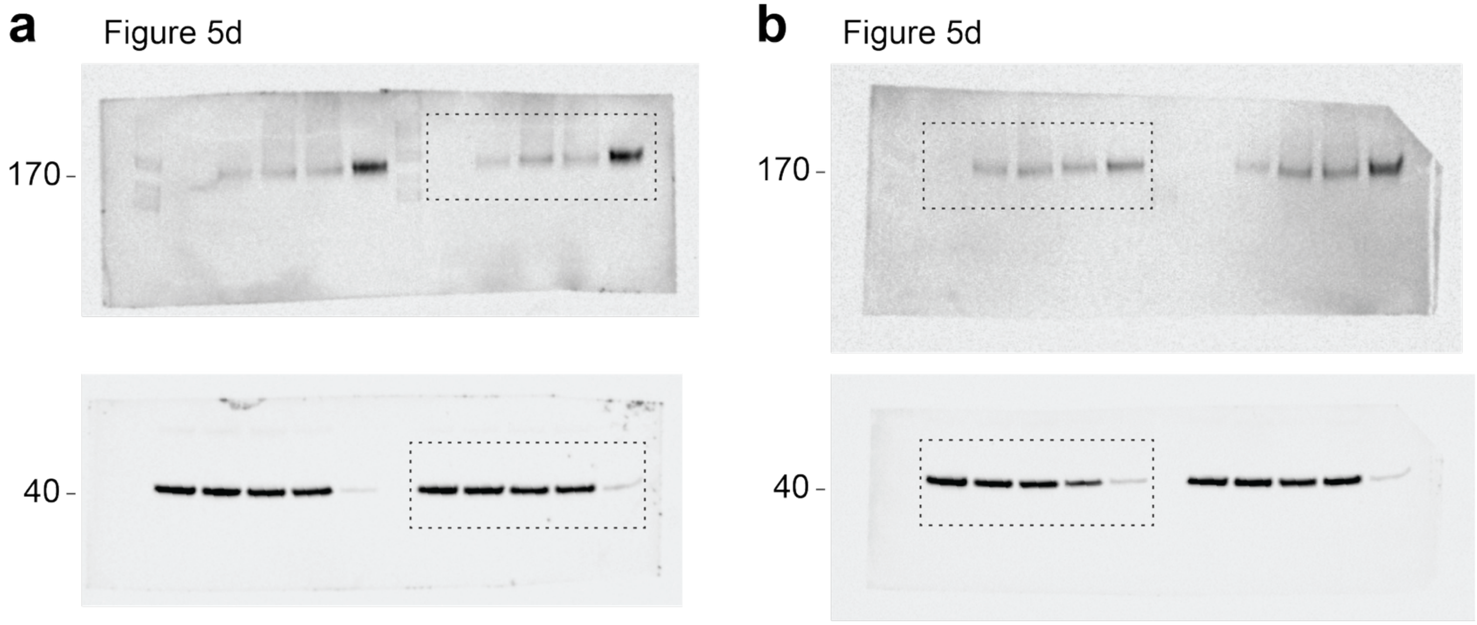

Supplementary Figure 13. Uncropped original Western blot data. (**a**,**b**) *Top:* NPC1 stained. *Bottom*: GAPDH stained. The main-text figure that includes each image is shown above each gel with a black outline to show the excerpted portion.

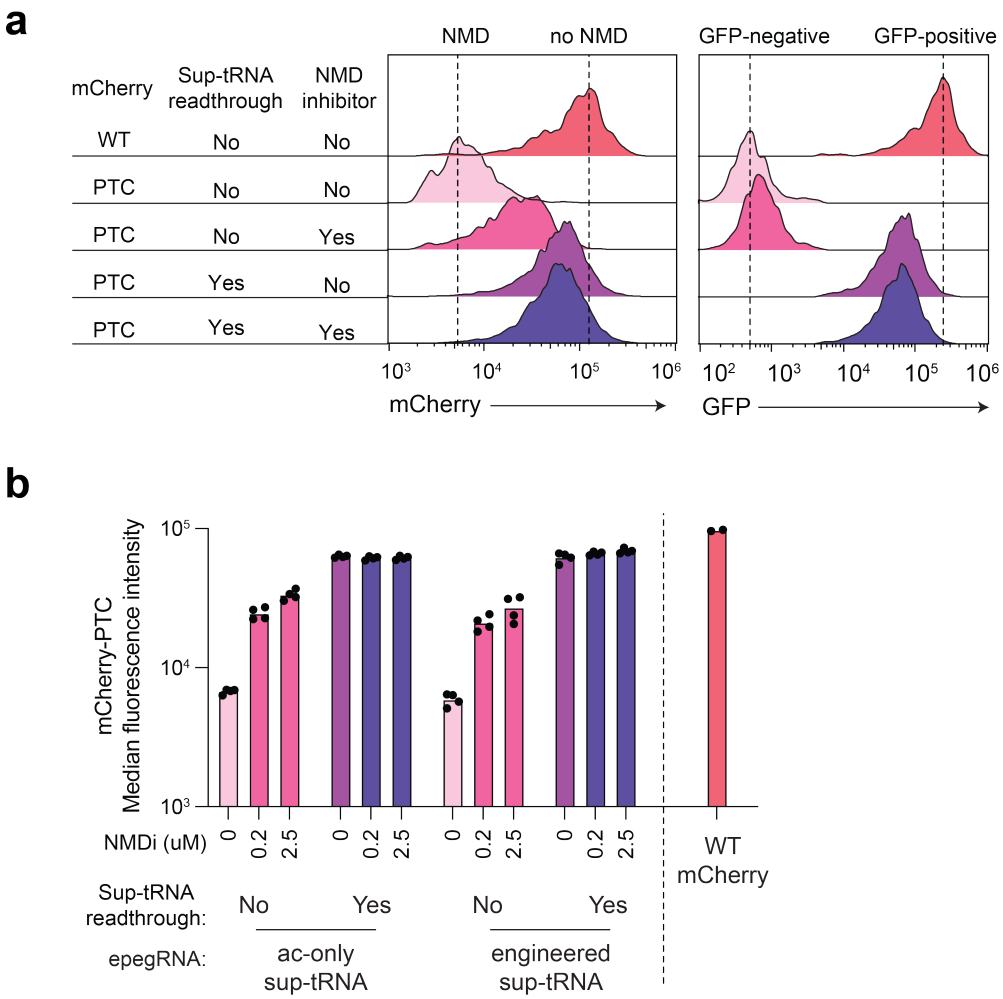

Supplementary Figure 14. Readthrough with a sup-tRNA inhibits nonsense-mediated decay. (**a**) Histogram showing mCherry and GFP fluorescence of cells expressing the pSEP0201 sup-tRNA reporter (mCherry-STOP-GFP) treated with NMD inhibitors (NMDi, KVS0001) and/or treated with prime editing agents to install a sup-tRNA. (**b**) Median fluorescence intensity of mCherry followed by a PTC for reporter cells (mCherry-STOP-GFP) treated with NMD inhibitors or treated with prime editing agents to install a sup-tRNA. N=4 independent biological replicates.

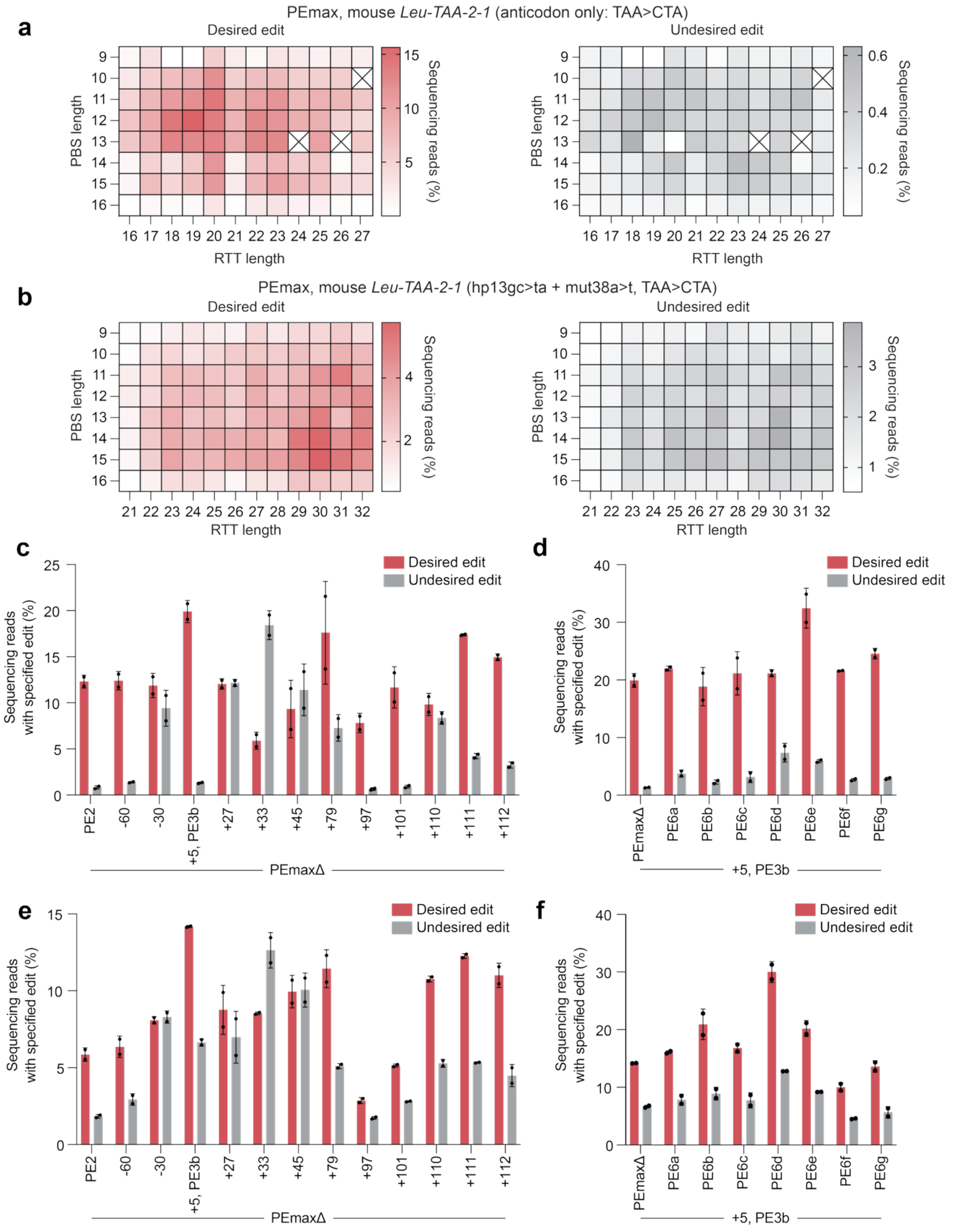

Supplementary Figure 15. Converting mouse tRNA-Leu-TAA-2-1 to a sup-tRNA with prime editing. (**a, b**) Heatmap of sequencing reads (%) with the desired edit (*left*) and undesired edit (*right*) for epegRNAs targeting mouse *tRNA-Leu-TAA-2-1* with the anticodon-only edit (a) or the edit additionally encoding the mutations hp13gc>ta and mut38a>t (b). (**c, d**) Sequencing reads with the specified edit for epegRNAs targeting mouse *tRNA-Leu-TAA-2-1* with the anticodon-only edit incorporated using the indicated ngRNAs (c) or prime editors (d). (**e,f**) Sequencing reads with the specified edit for epegRNAs targeting mouse *tRNA-Leu-TAA-2-1* with the edit additionally encoding the mutations hp13gc>ta and mut38a>t using the indicated ngRNAs (e) or prime editors (f). Values and error bars reflect mean±s.d. of n=2 independent biological replicates.
